# All the Lonely People: An Integrated Review and Research Agenda on Work and Loneliness

**DOI:** 10.1177/01492063241313320

**Published:** 2025-02-14

**Authors:** Julie M. McCarthy, Berrin Erdogan, Talya N. Bauer, Selin Kudret, Emily Campion

**Affiliations:** University of Toronto Scarborough; Portland State University; University of Exeter; Portland State University; University of Reading; University of Iowa

**Keywords:** emotions, social exchanges, well-being, work stress, health, social networks, groups, teams, networks, affect and emotions, job attitudes

## Abstract

Decades of studies spanning multiple disciplines have provided insight into the critical role of loneliness in work contexts. In spite of this extensive research, a comprehensive review of loneliness and work remains absent. To address this gap, we conducted a multidisciplinary review of relevant theory and research and identified 213 articles reporting on 233 empirical studies from management, organizational psychology, sociology, medicine, and other domains to uncover why people feel lonely, how different features of work can contribute to feelings of loneliness, and the implications of employee loneliness for organizational settings. This enabled a critical examination of the distinct conceptualizations and operationalizations of loneliness that have been advanced and the theories underpinning this scholarship. We developed a comprehensive conceptual model that integrates cognitive discrepancy theory, the affect theory of social exchange, and evolutionary theory. This model elucidates the core antecedents, mediators, outcomes, moderators, and interventions forming the nomological network of work related loneliness, including cross-level influences within teams and among leaders. Our review also identifies a number of promising areas for future inquiry to improve our understanding and measurement of loneliness, the process of experiencing and managing loneliness in the workplace, and potential interventions to reduce it. Finally, we provide tangible guidance for organizations and practitioners on how to address and mitigate employee loneliness. Ultimately, our review underscores the complex nature of loneliness and work and establishes a foundation for advancing both scholarly discourse and organizational practices in this critical domain.

“Given the significant health consequences of loneliness and isolation, we must prioritize building social connection the same way we have prioritized other critical public health issues . . .”

*—Dr. Vivek Murthy, U.S. Surgeon General*

Loneliness, which is characterized by the deprivation of vital social connections, is increasingly pervasive, affecting nearly one in four adults worldwide, or more than a billion people (Gallup, 2023). The situation has become so dire that, as shown in the opening quote, loneliness has been deemed a health epidemic in the United States by the U.S. surgeon general in a detailed report published in 2023 (U.S. Surgeon General’s Office, 2023). In some ways, this is not surprising, as the COVID-19 pandemic ushered in a new era in which loneliness became even more pronounced (Ernst et al., 2022). Unfortunately, post-pandemic shifts—such as increased remote work, growing dependence on technology, and heightened stress levels—have continued to amplify social disconnection (Caron, 2023; Wanberg, Csillag, Douglass, Zhou, & Pollard, 2020). Ultimately, the effect of increased digital connectivity and decreased face-to-face interactions, along with growing competitiveness and pressure in society, has served to isolate individuals in both their personal and work lives, contributing to the experience of loneliness.

This is deeply concerning, as humans have a fundamental need for interpersonal connections and belongingness (Baumeister, DeWall, Ciarocco, & Twenge, 2005; Baumeister & Leary, 1995). Unfortunately, many features of the modern workplace, such as remote work (Spilker & Breaugh, 2021), noninclusive work environments (Huang & Chan, 2022), the rise of technostress (Taser, Aydin, Torgaloz, & Rofcanin, 2022), and the integration of artificial intelligence (AI) (Tang et al., 2023), may serve to exacerbate feelings of loneliness. At the same time, whether an individual is employed or not is itself related to loneliness, with working adults reporting lower levels of loneliness compared to those not working (Bonsaksen et al., 2023). Given that many people spend the majority of their waking hours at work and often search for a sense of belongingness through their jobs (Gabriel, Lanaj, & Jennings, 2021), it is crucial to develop a thorough understanding of the connection between work and loneliness. This understanding is not only essential for fostering healthier work cultures and creating environments that prioritize belongingness but also for addressing the pervasive impact of loneliness on society as a whole.

Traditionally, loneliness has been associated with an individual’s personal life, with the predominance of research driven by the field of clinical psychology (Heinrich & Gullone, 2006), dating back to the 1950s (e.g., Fromm-Reichmann, 1959). By the 1980s, research began to explore how workplace experiences contribute to loneliness and, in turn, influence key behaviors and attitudes (e.g., Solomon, Mikulincer, & Hobfoll, 1986). Our systematic review uncovered a total of 213 articles that focus on loneliness in working adults. Despite this notable number of studies, a comprehensive review focusing on the connection between work and loneliness remains absent. Instead, existing reviews tend to be targeted and relatively narrow in nature. For example, there are reviews on loneliness and aging populations (Hawkley & Cacioppo, 2010), on health outcomes such as mortality (Wang et al., 2023), psychiatric disorders (West, Kellner, & Moore-West, 1986), physiological functioning (Hawkley, Masi, Berry, & Cacioppo, 2006), and on leader loneliness (Lam, Giessner, Shemla, & Werner, 2024). There are also meta-analyses of interventions that address social connection and loneliness (Hickin, Käll, Shafran, Manzotti, & Langan, 2021; Shah, Nogueras, Van Woerden, & Kiparoglou, 2019). Therefore, the need for a review of the work and loneliness literature is both timely and overdue.

Thus, our goal is to provide an up-to-date, theoretically grounded, and comprehensive integrated review of the research connecting work and loneliness, exploring its role as it relates to the workplace and its impact on individuals, teams, and organizations. Given that the literature on loneliness as it applies to work is multidisciplinary and diffuse, we sought to integrate theory and research across the fields of psychology, sociology, medicine, organizational behavior, and management in order to advance an integrated conceptual model that can serve as a foundation for future research. The existing literature on loneliness has been criticized for a lack of conceptual clarity, limited theoretical development, failure to focus on organizational implications, and insufficient intervention strategies (Firoz, Chaudhary, & Khan, 2021; Wright & Silard, 2021). Thus, our review seeks to offer guidance for researchers conducting studies in these areas, as well as a review of intervention strategies for managers, leaders, and practitioners to combat the negative effects of loneliness.

Our review is organized into five sections. First, we detail the process we followed to conduct our literature review. Second, we focus on the conceptualization and operationalization of loneliness as it pertains to work. Third, we advance an integrated model of loneliness at work that summarizes the theoretical underpinnings, as well as the core antecedents, outcomes, mechanisms, and moderators. In the fourth and fifth sections, we discuss future research directions, as well as the implications of loneliness for individuals, groups, and organizations in the work context.

## Literature Review Methodology

To conduct our review, we searched for articles relating work to loneliness using the PsycINFO and EbscoHost (Academic Search Premier and Business Source Premier) databases. Our online supplement (F1) presents a PRISMA figure outlining our process and resulting articles for review. Keywords searched were loneliness AND (work OR employee OR employment OR organization OR organisation OR workplace). A total of 4,270 abstracts were screened and marked for inclusion if the article focused on workplace loneliness, OR studied working adult populations and included a measure of general loneliness, OR studied general populations and included a variable related to work (such as employment status). Qualitative articles at the intersection of work and loneliness were also included. Finally, 26 articles were added through a Google Scholar search and through reference sections of the other articles, resulting in 213 articles focusing on work-related loneliness (i.e., studies conducted in a work context or including loneliness and a work-related construct). Thus, our focus includes all papers at the intersection of work and loneliness and, while we use the term *workplace loneliness* to explicitly denote situations where social needs *at work* are not being met (Ozcelik & Barsade, 2018), the focus of our review also extends to papers that have studied general levels of loneliness with working adults. Online supplement S1 provides a summary of the main characteristics of these papers, including sample characteristics, study design, measures used, and geographical focus. We also include a supplemental table (S2) with the specific sample, research design, journal characteristics, and primary findings of the 213 papers reviewed (which report on 233 empirical studies). We next turn our attention to loneliness as defined and examined by researchers.

## The Nature of Loneliness

### Defining, Conceptualizing, and Operationalizing Loneliness

Loneliness is a multifaceted phenomenon that has been defined, theorized about, and studied from a variety of perspectives, reflecting its inherent complexity (see Table S3 for core definitions in the field). We advance the following definition of loneliness, and in the following sections, describe how our integration of the literature informed this definition:

*Loneliness refers to a discrepancy between desired and actual levels of social connection that encompasses the following six core elements*:

(1) *Loneliness occurs when there is a perceived deficiency in social relationships*.
(2) *Loneliness is a subjective experience.*
(3) *Loneliness is unpleasant and distressing.*
(4) *Loneliness affects individuals emotionally, cognitively, and behaviorally.*
(5) *Loneliness can present as a transient affective state or an enduring traitlike disposition.*
(6) *Loneliness is a multidimensional construct containing social and emotional components.*

To begin, a careful analysis of the research on loneliness reveals widespread agreement that loneliness is rooted in the fundamental human need for social connection—specifically, the needs for belongingness and relatedness (Baumeister & Leary, 1995; Maslow, 1943; McClelland, 1961). When these essential social needs are not fulfilled, individuals are predisposed to experience loneliness (Cacioppo, Grippo, London, Goossens, & Cacioppo, 2015; Peplau & Perlman, 1982). Importantly, loneliness is characterized by a perceived discrepancy between the quality and quantity of social relationships one desires and those one actually possesses. Consequently, loneliness is inherently relational and subjective, allowing individuals to feel lonely even when surrounded by others (Cacioppo et al., 2006; Ozcelik & Barsade, 2018). Finally, loneliness reflects an unpleasant subjective experience, as depicted in Weiss’s (1973) characterization of loneliness as “gnawing distress . . . without redeeming features” (p. 15). Taken together, there are three core aspects of loneliness that are embodied in the majority of research in the field, principles that were first delineated by Peplau and Perlman (1982), which include (1) *loneliness occurs when there is a perceived deficiency in social relationships*, (2) *loneliness is a subjective experience*, and (3) *loneliness is unpleasant and distressing*. Our comprehensive review suggests that there are three additional aspects essential to the conceptualization and operationalization of loneliness: (4) *expression of loneliness*, (5) *chronicity of loneliness*, and (6) *dimensionality of loneliness*. We describe each of these in detail later and highlight their key characteristics in Table 1.

**Table 1:** Conceptualization of Loneliness

Conceptual Aspect	Description
**Expression of Loneliness** Loneliness affects individuals affectively, cognitively, and behaviorally.	**Affective**: Reflects the emotional distress triggered by perceived social disconnection. **Cognitive**: Reflects the way in which lonely individuals process their thoughts. **Behavioral**: Reflects the behavioral patterns that lonely individuals exhibit.
**Chronicity of Loneliness** Loneliness can be chronic, reflecting an enduring statelike disposition, or situational, reflecting an affective state.	**Chronic**: A recurring affective state with a stable set of defining features that endure over time. **Situational**: Feelings of loneliness arising from temporary circumstances or events.
**Dimensionality of Loneliness** Loneliness is a multidimensional construct that includes a broad, higher-order factor capturing the general experience of feeling lonely and two core underlying dimensions—social and emotional.	**Social**: The experience of loneliness due to a lack of social interactions or connections, often linked to the quantity of one’s social network. **Emotional**: The feeling of loneliness arising from the absence of deep, meaningful relationships and intimate connections
**Other Relevant Constructs** Loneliness is related to, but distinct from, a number of relevant constructs.	**Solitude**: When being alone is a pleasant state. **Depression**: A broader mental health condition where loneliness may be one contributing factor. **Hopelessness**: A state of despair about the future where loneliness may be one contributing factor. **Ostracism**: Active social rejection which can lead to loneliness but also includes feelings of rejection and worthlessness. **Isolation**: A lack of social relations and contacts that can result in loneliness.

### Expression of Loneliness

The loneliness literature suggests the deficiency of social relationships affects individuals emotionally, cognitively, and behaviorally (Heinrich & Gullone, 2006; Ozcelik & Barsade, 2018; Peplau & Perlman, 1982). This aligns with the affective prototype approach (Chaplin, John, & Goldberg, 1988), which posits that affective experiences, such as loneliness, are organized around clusters of defining features—such as emotional states, cognitive patterns, and behavioral tendencies—that form distinct prototypes. The affective feature reflects the emotional distress triggered by perceived social disconnection and is often characterized by an unpleasant or painful yearning for social connection (Ponzetti, 1990). This manifests in a wide array of negative emotional states, including sadness (Heinrich & Gullone, 2006), anxiety (Anzola, Limoges, McLean, & Kolla, 2022; Blomqvist, Virtanen, Westerlund, & Magnusson Hanson, 2023), and aggression (Twenge, Baumeister, Tice, & Stucke, 2001). In other words, loneliness is often accompanied by negative emotions.

Loneliness also affects people cognitively, shaping how they process their thoughts (Peplau & Perlman, 1982). This includes the attributions they make about themselves and others (Teneva & Lemay, 2020), their perceived control over social situations (Anderson, Miller, Riger, Dill, & Sedikides, 1994), and their capacity to concentrate and stay focused on tasks (Park et al., 2020). Importantly, these affective and cognitive features of loneliness are likely to be bidirectional, as both theoretical frameworks and empirical research suggest (Lazarus, 1991; LeDoux, 1989). Specifically, cognitive processes, such as negative self-evaluations, directly shape emotional responses like anxiety and sadness (Liu, Yang, Wu, Kong, & Cui, 2020; Mor & Winquist, 2002). In turn, these emotions further intensify maladaptive cognitive patterns, such as negative self-evaluations (Brown & Mankowski, 1993), and negative perceptions of social interactions (Pozo, Carver, Weflens, & Scheier, 1991).

The ongoing interaction between cognitions and emotions, in turn, drives behavioral effects of loneliness, including social withdrawal, inhibited sociability, and ineffective interpersonal behaviors (Heinrich & Gullone, 2006). Lonely individuals often hesitate to engage in social interactions, making it difficult to form friendships or join group activities (Cacioppo et al., 2000). They struggle with maladaptive self-disclosure, either oversharing or withholding, leaving others feeling disconnected (Solano, Batten, & Parish, 1982). Lonely individuals also tend to cope passively—by sleeping, doing nothing, or watching television (Rubenstein & Shaver, 1982)—or by reducing effort toward tasks (Cacioppo et al., 2000). These behaviors, intertwined with negative emotions and cognitions, reinforce social disconnection and perpetuate a cycle of loneliness.

### Chronicity of Loneliness

The loneliness literature emphasizes that the time frame under which individuals feel lonely is consequential. The experience of loneliness can be *transient*, with the individual experiencing brief and occasional feelings of loneliness. Alternatively, this experience can be *chronic*, with the individual persistently feeling that their relationships are deficient over extended periods of time.

This distinction is critical in understanding the implications of loneliness for emotions, cognitions, and behaviors. Cacioppo et al. (2006) suggested that as long as it is not persistent, loneliness can serve an adaptive function, such as motivating individuals to reach out to others and make an effort to reduce their loneliness. Much like physical pain that alerts the individual to take protective action, transient loneliness creates emotional pain and alerts the person that their social needs are not being met. For instance, someone who feels lonely after starting a new job may be motivated to engage with colleagues to build new relationships. In contrast, the chronic experience of loneliness is thought to be associated with dysfunctional cognitive processes (Heinrich & Gullone, 2006). Chronic loneliness can be debilitating because it becomes ingrained in an individual’s emotional and cognitive processes, creating a cycle where persistent loneliness exacerbates feelings of helplessness and social withdrawal (Cacioppo et al., 2015; Hawkley & Cacioppo, 2010). This cycle aligns with learned helplessness theory (Maier & Seligman, 1976), where continued exposure to negative social stimuli reinforces negative beliefs, making loneliness an enduring, self-perpetuating experience (Maier & Seligman, 2016).

As is typical of the loneliness literature at large, most of the research in our review conceptualized and operationalized loneliness as a chronic phenomenon or an enduring traitlike feeling. Only a small number of studies positioned loneliness as a more statelike construct that can be activated by circumstance. These studies provide insight into how loneliness fluctuates with specific situational triggers, such as a major life transition or temporary isolation. Loneliness, therefore, can manifest either as a generalized perception that applies broadly to one’s lived experiences or as a transient feeling that fluctuates over time and conditions. For example, Ozcelik and Barsade (2018) assessed loneliness as a more chronic condition by asking participants to report their overall, or general, levels of workplace loneliness, whereas Gabriel et al. (2021) examined the implications of transient loneliness by collecting data on daily loneliness levels.

Given these distinctions, it is essential for researchers to explicitly consider the time frame in order to operationalize loneliness in alignment with their specific research objectives. For example, studies examining interventions designed to alleviate chronic loneliness should employ measures that capture stable, enduring patterns of loneliness over time. In contrast, researchers examining the situational or contextual variability of loneliness should utilize measures that are sensitive to short-term fluctuations and capable of tracking loneliness across specific episodes or time periods. For instance, state-based research might investigate how loneliness fluctuates for employees working in remote or hybrid environments, particularly in response to virtual interactions with colleagues or periods of physical isolation while working from home. Our review of existing scales (see Table S4) revealed that although existing instruments were primarily developed to measure persistent, dispositional loneliness (e.g., Russell, Peplau, & Cutrona, 1980), they can be adapted to assess more momentary, situational fluctuations (Gabriel et al., 2021). Employing the appropriate measure is critical to ensuring that findings accurately reflect the type of loneliness under investigation, thus facilitating a deeper understanding of how loneliness operates across different contexts and temporal frames.

### Dimensionality of Loneliness

The majority of research on loneliness in the workplace has relied on a unidimensional, or overall, conceptualization of loneliness, predominantly measured using the UCLA Loneliness Scale (Russell et al., 1980) or its shortened versions (Hughes, Waite, Hawkley, & Cacioppo, 2004; see Table S4 for details of the measures utilized in studies we reviewed). However, there is emerging empirical evidence supporting a multidimensional perspective that distinguishes between social and emotional components (e.g., DiTommaso & Spinner, 1997; Rubenstein & Shaver, 1982). This framework aligns with Weiss’s (1973) theoretical lens, which differentiates between a social dimension—representing the presence of a broad network of reliable connections—and an emotional dimension—reflecting the presence of intimate and meaningful relationships. Several multidimensional measures of loneliness have been developed based on these components (De Jong-Gierveld & Kamphuis, 1985; De Jong-Gierveld & Van Tilburg, 2006; DiTommaso & Spinner, 1993; Wright, Burt, & Strongman, 2006). Further, research shows that the emotional and social components are uniquely associated with various psychological and behavioral outcomes (DiTommaso & Spinner, 1997; Rubenstein & Shaver, 1982). For example, research suggests that compared to social loneliness, emotional loneliness is more strongly correlated with constructs such as emotional exhaustion, depression, and lack of psychological empowerment (e.g., Jung, Jung, & Yoon, 2022; Kanbur & Kanbur, 2020; Wolters et al., 2023). In contrast, social loneliness has been found to be more strongly correlated with constructs such as introversion, social isolation, and low levels of online social network connectivity, as compared to emotional loneliness (DiTommaso & Spinner, 1993; Erevik, Vedaa, Pallesen, Hysing, & Sivertsen, 2023; Hopp et al., 2022).

At the same time, Russell, Cutrona, Rose, and Yurko (1984) demonstrate that while these dimensions are distinct, they share a common core experience. In other words, they note that the overarching experience of loneliness is supported by two interrelated dimensions: emotionality and social-cognitive aspects. In other words, loneliness is best understood as a multifaceted, higher-order (bifactor) construct. This higher-order conceptualization of loneliness has been supported across several studies (e.g., Alsubheen, Oliveira, Habash, Goldstein, & Brooks, 2023; Grygiel, Humenny, & Rębisz, 2019). This means that depending on the research objective, it may be appropriate to focus either on an overall higher-order loneliness factor or to examine its specific dimensions as subfactors.

### Differences from Other Constructs

It is crucial to distinguish loneliness from other related constructs, such as social isolation, solitude, ostracism, hopelessness, and depression. Social isolation refers to a state of having little contact with others and is a more objective reality that is observable by others (Steptoe, Shankar, Demakakos, & Wardle, 2013). This is different from loneliness, which is a perceptual assessment of the quality and quantity of one’s relationships. Solitude reflects a positive and often sought-after experience of being alone, whereas loneliness is characterized by an unpleasant sense of social disconnection and the absence of desired companionship (Long & Averill, 2003). Loneliness is also distinct from ostracism, which involves active social rejection by others (Howard, Cogswell, & Smith, 2020). While ostracism may lead to feelings of loneliness, it also encompasses a broader range of emotions, including rejection and helplessness, that go beyond the experience of loneliness itself (Williams, 2007). Additionally, loneliness should not be conflated with hopelessness, which is a state of despair and pessimism about the future. While loneliness can contribute to feelings of hopelessness, it is just one of many potential factors that can lead to such a state. Lastly, loneliness is distinct from depression, a complex and pervasive mental health condition. Although loneliness is a risk factor for depression (Erzen & Cikrikci, 2018), depression encompasses a wider array of emotional, cognitive, and physiological symptoms, extending beyond the social disconnection that characterizes loneliness (Richards, 2011).

## Conceptual Model of Loneliness and Work

### Theoretical Foundation

**Figure 1:**
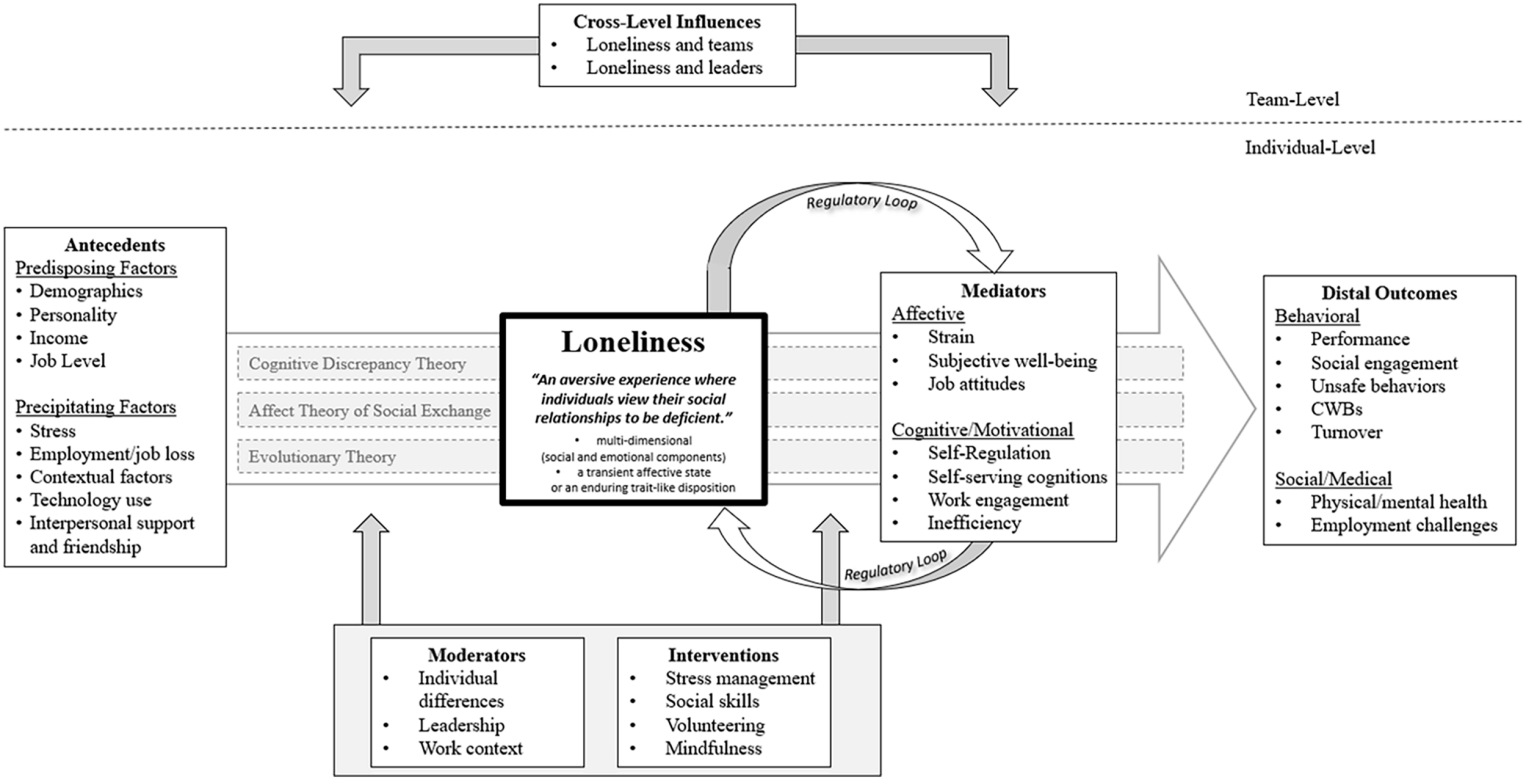
Conceptual Model of Loneliness and Work

The foundational theorizing underlying the loneliness literature are social-needs theories, or those that view loneliness as a consequence of unmet social needs. From this foundation, researchers have enriched the loneliness framework by leveraging three other theoretical streams: social exchange theories, socio-psychological theories, and evolutionary theories. Table 2 outlines the specific theories, core theoretical arguments, and sample studies for each of these perspectives. Our conceptual framework (Figure 1) integrates three theories from these streams: *cognitive discrepancy theory* (Peplau & Perlman, 1982), a socio-psychological theory; the *affect theory of social exchange* (Lawler, 2001), a social exchange theory; and *evolutionary theory* (Cacioppo & Cacioppo, 2018).

**Table 2:** Theoretical Perspectives in Current Research at the Intersection of Loneliness and Work

Broad Theoretical Perspectives	Specific Theories	Major Arguments	Studies Utilizing Theory
**Social Needs–Based Theories**			
• Highlighted the role of unmet social needs as the root cause of loneliness. • Focused on the existence of social needs but did not account for the interpersonal nature of loneliness, including the amount and quality of those interactions.	Need for social connection(Baumeister & Leary, 1995)	When individuals are not able to meet their inherent need for social connection, they experience loneliness.	• Anand and Mishra (2021) • Lam and Lau (2012) • Waytz, Chou, Magee, and Galinsky (2015)
	Self-determination theory (Deci & Ryan, 1985; Ryan, 1982)	Fulfilment of the need for relatedness is essential for psychological well-being, and loneliness can be a signal that this need is unmet.	• Huang and Chan (2022) • Tian, Pu, and Ren (2021) • Wright and Silard (2021)
	Hierarchy of needs (Maslow, 1943)	Individuals have an innate need for belongingness—they are motivated to establish relationships with people.	• Chen, Wen, Peng, and Liu (2016) • Kanbur and Kanbur (2020) • Poulsen and Ipsen (2017)
**Social Exchange Theories**			
• Expanded on social-needs theories by highlighting that it is not just the need for connection that matters but also the amount and quality of social interactions that one has .• Focuses on the consequences of loneliness, particularly how it leads to lower-quality social interactions and weaker social bonds.	Social exchange theory (Maslow, 1943; McClelland, 1961)	Lonely people struggle to establish social exchange relationships due to low trust, poor self-evaluations, and social risk aversion, leading to negative outcomes for individuals and organizations.	• Chen et al. (2016) • Lam and Lau (2012) • Peng, Chen, Xia, and Ran (2017)
	Affect theory of social exchange(Lawler, 2001)	Emotions generated through social exchange influence the formation and maintenance of social bonds: positive emotions strengthen relationships, while negative emotions lead to withdrawal.	• Gilmer, Magley, Dugan, Namazi, and Cherniack (2023) • Kloutsiniotis, Mihail, Mylonas, and Pateli (2022) • Ozcelik and Barsade (2018)
**Socio-Psychological Theories**			
• Focused on the antecedents of loneliness and the influence of both individual characteristics (personality traits, emotions) and societal influences (cultural values, social norms, urbanization) .• Individual characteristics and societal influences can interact to influence the experience of loneliness.	Characterological theory of loneliness (Weiss, 1973)	• Loneliness consists of emotional and social components that are influenced by personality traits, thought patterns, and external events. • Personal dispositions and situations interact to influence loneliness. • Feelings of loneliness impede the formation and maintenance of satisfying social relationships.	• Pinedo González, Palacios Picos, and de la Iglesia Gutiérrez (2021) • Wright and Silard (2021) • Wright et al. (2006)
	Cognitive discrepancy theory (Peplau & Perlman, 1982)	• Highlights the gap between actual and desired interpersonal connections. • Distinguishes predisposing factors (e.g., introversion) and precipitating factors (e.g., work stress) as loneliness antecedents. • Categorizes outcomes into affective, cognitive, behavioral, and social/medical.	• Luk et al. (2023) • Ong (2022) • Surkalim, Clare, Eres, Gebel, Bauman, and Ding (2023) • Wels et al. (2023) • Wickens et al. (2021)
**Evolutionary Theories**			
• Loneliness is a biological signal that motivates one to seek and maintain relations. • Loneliness is a driver of specific outcomes that can be adaptive and maladaptive.	Evolutionary theory of loneliness (Cacioppo & Cacioppo, 2018)	• Loneliness is a biological signal rooted in human evolution, which serves as a cue that prompts humans to seek and maintain relationships. • Loneliness, while unpleasant, is a normal and natural response to perceived social isolation, which can help propel the restoration or strengthening of social connections.	• Eisenberger, Lieberman, and Williams (2003) • Gabriel et al. (2021) • Tang et al. (2023)
	Regulatory loop model of loneliness (Cacioppo & Hawkley, 2009)	Loneliness increases awareness of social threats, leading to insecurity and a cycle of negative social experiences and heightened loneliness. Loneliness can drive adaptive changes, encouraging behaviors that improve cooperation and strengthen social integration.	• Andel, Shen, and Arvan (2021) • Gabriel et al., 2021 • Ozcelik and Barsade (2018)

#### Cognitive discrepancy theory

This theory has been a foundational element in a substantial number of the studies we reviewed, as it not only incorporates key elements of the social needs–based theories—by highlighting the discrepancy between individuals’ actual and desired levels of interpersonal connection—but also offers a robust framework for examining both the antecedents and outcomes of loneliness. Cognitive discrepancy theory identifies two categories of loneliness antecedents: predisposing factors, which reflect enduring individual characteristics, and precipitating factors, which involve situational influences. Cognitive discrepancy theory also provides a detailed framework for understanding the outcomes of loneliness by categorizing them into four key manifestations: affective outcomes (e.g., depression, dissatisfaction), motivational/cognitive outcomes (e.g., difficulty concentrating, self-consciousness), behavioral outcomes (e.g., social withdrawal, reduced communication), and social/medical problems (e.g., illness, exhaustion). As illustrated in Figure 1, we align our model with these categories of antecedents and outcomes while at the same time providing greater granularity by distinguishing among mediators and distal outcomes, moderators, interventions, and cross-level influences.

#### Affect theory of social exchange

While cognitive discrepancy theory (Peplau & Perlman, 1982) incorporates affective manifestations of loneliness, such as anxiety, it does not fully capture the affective social dynamics that unfold when individuals experience loneliness in social and organizational contexts. The affect theory of social exchange (Lawler, 2001) bridges this gap by illustrating how emotions and feelings shape the affiliative bonds individuals form with others, whether in dyadic relationships or larger networks, such as teams or organizations. Specifically, the theory posits that emotions become intertwined with social units—be it individuals or groups—through the cultivation of affective bonds. This emotional attachment is crucial for forging and maintaining strong social connections. When individuals feel lonely, the perceived level of attachment or affiliation to others, such as coworkers, leaders, or teams, diminishes (Lawler, Thye, & Yoon, 2008). The theory further proposes that through the process of social attributions, individuals may experience common, or shared, emotions such that individual emotions become “collective” emotions (Lawler, Thye, & Yoon, 2014). Taken together, this model predicts that debilitative social exchange relations can precipitate feelings of loneliness, which can be attributed to individuals or group members and linked to lower levels of affiliation. In turn, this reduced affiliation can lead to withdrawal behaviors. We integrate the affect theory of social exchange into our framework to not only highlight the complex role that emotions play in the experience of loneliness (as illustrated in Figure 1, loneliness is directly related to affective mediators) but also to delineate the role that one employee’s loneliness can have on a team and/or a leader (and vice versa). This cross-level approach allows us to explore the broader consequences of loneliness, demonstrating how emotional experiences at the individual level can affect teams and leaders and vice versa.

#### Evolutionary theory

Evolutionary theory suggests that loneliness can serve as a warning signal to the individual, motivating them to reconnect with others. A key element of evolutionary theory is the regulatory loop model (Cacioppo & Hawkley, 2009), which explains how loneliness functions as a self-perpetuating cycle. If efforts to reconnect are successful, the individual will feel less lonely. However, if unsuccessful, and loneliness becomes persistent over extended periods, such adaptive effects disappear. Instead, lonely individuals become hyper-vigilant to social threats, leading them to form negative social expectations. These expectations trigger behavioral confirmation processes, where lonely individuals withdraw from others or act defensively, further reinforcing their sense of isolation. This cycle creates a self-sustaining feedback loop that perpetuates loneliness. As shown in Figure 1, our framework incorporates this regulatory loop by modeling a feedback loop for the affective and cognitive consequences of loneliness, such as anxiety, rumination, and negative self-perception. Additionally, these affective and cognitive outcomes lead to downstream behavioral and social/medical consequences, such as social withdrawal, reduced communication, and even health deterioration. These individual outcomes can extend beyond the person, influencing team dynamics and organizational functioning.

Together, these three theories provide the foundation for a comprehensive, multilevel loneliness framework. Cognitive discrepancy theory captures the antecedents (*predisposing, precipitating factors*), mechanisms (*affective, cognitive/motivational*), and outcomes (*behavioral, social/medical*) of loneliness. The affect theory of social exchange explains the role of emotions and social interactions in individuals and teams (*cross-level influences*), while the evolutionary theory emphasizes the biological and behavioral responses, including the self-reinforcing *regulatory loop*.

### Antecedents: Predisposing Factors

Cognitive discrepancy theory (Peplau & Perlman, 1982) highlights predisposing factors as antecedents of loneliness, such as self-esteem and shyness. Our review uncovered four elements within this category: demographics, personality, income, and job level.

#### Demographics

Most studies we reviewed found that women tended to experience greater feelings of loneliness than men (Gunes & Bilek, 2020; Lin, 2023; Petrovski & Gleeson, 1997; Wickens et al., 2021), with additional research suggesting that women working from home report higher levels of loneliness compared to their male counterparts (Wels et al., 2023). These findings align with the understanding that women generally exhibit a higher need for affiliation than men (Drescher & Schultheiss, 2016). However, this is inconsistent with the broader literature wherein meta-analytic findings are either suggestive of no gender differences in loneliness (Maes, Qualter, Vanhalst, Van den Noortgate, & Goossens, 2019) or find that men report higher levels of loneliness than women (Barreto, Victor, Hammond, Eccles, Richins, & Qualter, 2021). Given that our focus was on research at the intersection of loneliness and work, it is plausible that the inclusion of predominantly working adult samples may have influenced these results. Specifically, men may find it easier to establish connections at work because of their tendency to occupy more central positions in interpersonal networks (Fang, Zhang, and Shaw, 2021) and to have stronger network ties (Ibarra, 1992). The affect theory of social exchange (Lawler, 2001) suggests that these social connections generate emotional rewards, potentially reducing loneliness more effectively for men.

Our review also suggests that the relationship between age and loneliness is complex. While several studies show that loneliness increases with age (Cacioppo et al., 2016; Franssen, Stijnen, Hamers, & Schneider, 2020; Smith-Appelson, Reynolds, & Grzywacz, 2021), Luhmann and Hawkley (2016) and Lasgaard, Friis, and Shevlin (2016) found the highest loneliness levels among the youngest and the oldest individuals in their samples. More specifically, Lasgaard et al. (2016) found that loneliness levels tended to peak in older-aged groups who were not working. These findings align with the broader literature, wherein age has been found to have a U-shaped curve with loneliness, such that it decreases slightly from young adulthood to mid-life and then increases substantially in older adults (Graham et al., 2024). These findings are likely due to a number of factors for older adults, including lower income levels, a higher proportion of singles, and an increased propensity to live alone (Graham et al., 2024; Luhmann & Hawkley, 2016). For adolescents, high levels of loneliness have been attributed, in part, to the stage of development that is associated with increased development of social and interpersonal skills and increased independence (Hemberg, Östman, Korzhina, Groundstroem, Nyström, & Nyman-Kurkiala, 2022).

Finally, although few studies have considered racial differences, one notable exception is a study by Wanberg et al. (2020), which found that during COVID-19, loneliness was lower among non-Hispanic Black compared to non-Hispanic White, Hispanic, and Asian/Pacific Islander individuals. Additionally, findings show that perceived discrimination, whether based on race or sexual orientation, is linked to increased loneliness. Di Napoli et al. (2021) found that Italian immigrants facing discrimination experienced higher loneliness, negatively affecting their mental and physical health. Negi, Siegel, Sharma, and Fiallos (2021) reported that perceived racism led to loneliness among immigrant day workers. Similarly, Huang and Chan (2022) found that lack of LGBT+ friendliness increased loneliness and reduced well-being among LGBT+ individuals. McFadden and Crowley-Henry (2018), through a qualitative study, highlighted that LGBT+ employees often experience workplace isolation, but support networks can help alleviate loneliness.

#### Personality

The broad literature on loneliness has also examined links between loneliness and personality dimensions, notably the Big Five (Buecker, Maes, Denissen, & Luhmann, 2020), with meta-analytic findings suggesting that extraversion, agreeableness, and conscientiousness are negatively related, neuroticism is positively related, and openness is unrelated to loneliness. These findings align with the evolutionary theory of loneliness (Cacioppo & Cacioppo, 2018), which posits that certain traits may predispose individuals to experience loneliness—particularly high levels of neuroticism and low levels of extraversion—and that these traits are shaped by genetic factors (Abdellaoui et al., 2019; Cacioppo et al., 2006). Specifically, research shows substantial genetic coheritability between loneliness and personality traits such as neuroticism and extraversion (e.g., Abdellaoui et al., 2019, Gao, Davis, & Palmer, 2017; Schermer & Martin, 2019), meaning that the genetic influences that contribute to these traits also overlap with those driving loneliness. However, the role of personality as it pertains to *work-related* loneliness has been less frequently explored. That said, research has started to expand beyond the Big Five to include relevant constructs. For example, Anand and Mishra (2021) found that nurses and call center professionals with high core self-evaluations are less likely to report loneliness, and Lee (2022) found that self-efficacy and self-control were less related to loneliness.

Other research has shown that workaholic-like tendencies (e.g., perfectionism, workaholism) are related to loneliness, likely due to the overcommitment to work at the expense of relationships (Bovornusvakool, Vodanovich, Ariyabuddhiphongs, & Ngamake, 2012). Similarly, Bayraktar and Jiménez (2022) found that obsessive passion, defined as a strong urge to do something, was positively related to loneliness among small business owners in Turkey. Finally, an individuals’ social intelligence has been explored in the context of loneliness and work. Silman and Dogan (2013) found that social information processing, social skills, and social awareness were negatively related to the emotional dimension of workplace loneliness, while social skills and social awareness were negatively related to the social dimension.

#### Income

There is a large cluster of studies examining relations between loneliness and income, with findings converging on the fact that loneliness is lower among those with a higher income and fewer financial worries (e.g., Bower et al., 2023; Luk et al., 2023; Spiro et al., 2021; Wanberg et al., 2020). For example, Allen and Oshagan (1995) found that lower income was associated with higher loneliness among adults in the United States, and Mullins, Sheppard, and Andersson (1991) found that feelings of income inadequacy were associated with higher loneliness among adults in Sweden. Mullins et al. (1991) note that this finding is likely due to the fact that income can facilitate the maintenance of a lifestyle that promotes interpersonal activities and social connections. Financial security may also play a role in buffering the stressors that contribute to feelings of loneliness.

#### Job level

Many studies have found that being in leadership roles can be lonely (e.g., Dor-Haim & Oplatka, 2021; Gabriel et al., 2021; Nichols & McBride, 2017; Ong, 2022). Nichols and McBride (2017) found that teachers recently promoted to leadership positions experienced increased loneliness due to weaker interpersonal relations with their former peers. Similarly, Ong (2022) utilized time-lagged and experimental data and found that women experience higher workplace loneliness after promotion to management compared to men. This heightened loneliness for women may stem from gender-based leadership norms, biases, and stereotypes (e.g., Eagly & Johannesen-Schmidt, 2001), which negatively influence the interpersonal support female leaders receive (Hideg & Shen, 2019). In contrast, studies by Wright (2012) and Silard and Wright (2022) found no significant difference in workplace loneliness between managers and nonmanagers, while Waytz et al. (2015) provided experimental evidence that employees with lower levels of power report being more lonely.

#### Summary and implications

The studies in this category span a vast array of countries and occupations, including work contexts as diverse as healthcare, education, and remote work, across regions such as North America, Europe, and Asia. At the same time, many rely on simple, descriptive designs that focus on correlational relationships between loneliness and singular predisposing factors. While these findings provide a foundational understanding, they lack the complexity needed to explore how these factors interact with one another, such as gender and personality, or with precipitating factors, such as the workplace environment or technology use. Further, this research remains fragmented, with findings often presented in isolation rather than being integrated into a cohesive framework. This piecemeal approach limits the ability to make strong conclusions about the processes through which these antecedents contribute to loneliness. For instance, research examining the nuanced interplay between gender, leadership roles, and interpersonal support across diverse work settings could offer critical insights into the underlying mechanisms driving workplace loneliness, paving the way for more targeted and effective interventions.

### Antecedents: Precipitating Factors

Perlman and Peplau’s (1981) model also identified precipitating factors contributing to loneliness, such as changes in social relations. Our review identified five factors that fall within this category: stress, employment/job loss, contextual factors, technology use, and interpersonal support/friendship.

#### Stress

In line with the affect theory of social exchange (Lawler, 2001), which links negative affect to loneliness, several studies suggest that stress contributes to loneliness (e.g., Cacioppo et al., 2016; Fry & Bloyce, 2017; Wright et al., 2006). These studies have tended to focus on unique populations, such as professional golfers (Fry & Bloyce, 2017), soldiers (Cacioppo et al., 2016), and physicians (Aase, Nordrehaug, & Malterud, 2008). A number of stressors have been identified, including time and role-based demands (Luck, Doucet, & Luke, 2022), transitioning between life stages (Fardghassemi & Joffe, 2022), mental health disorders (Bower et al., 2023), and workplace competition and pressure (Aase et al., 2008). These findings are consistent with research showing that stress serves as a barrier to receiving support from one’s manager (Erdogan, Kudret, Bauer, McCarthy, Campion, & Chang, 2024). While these studies are valuable, the majority are cross-sectional in nature, precluding an understanding of whether stress begets loneliness or vice-versa (three exceptions are time-lagged studies by Bower et al., 2023; Smith, Ford, & Steffen, 2019; Walz, Kensbock, De Jong, & Kunze, 2024). Further, grounded in employee well-being and social exchange theories, a study by Walz et al. (2024) predicted that remote work would create work and home interference but that job and home support would mitigate such effects. As predicted, heightened demands from work and home increased employee work-to-home and home-to-work interference, respectively, resulting in a significant positive effect on workplace loneliness. This relationship was buffered by job support.

#### Employment status, types of employment, and job loss

A number of studies have revealed that simply being employed is associated with lower levels of loneliness relative to being unemployed (e.g., Bonsaksen et al., 2023; Hagen, Lai, & Goldmann, 2022; Surkalim et al., 2023). For example, in a longitudinal study, Buecker, Denissen, and Luhmann (2021) found increases in loneliness after job loss. Surkalim et al. (2023) found that employed individuals reported the lowest levels of loneliness, retired individuals reported moderate levels, and those who were unemployed faced the highest levels. The findings indicated that retired and unemployed individuals had an 88% and 96% higher risk, respectively, of experiencing sustained loneliness. Relatedly, self-employment has been found to be related to increases in loneliness (Gevaert, Bosmans, De Moortel, & Vanroelen, 2023), as has overall job insecurity (Andel et al., 2021; Bennett, Manchaiah, Eikelboom, Badcock, & Swanapoel, 2022; Blomqvist et al., 2023). Being employed in temporary (Moens, Baert, Verhofstadt, & Van Ootegem, 2021) or part-time (Bornstein & Magnus, 2022; Creed & Reynolds, 2001) work has also been found to be positively related to loneliness compared to those working full-time. These findings are likely due to reduced opportunities for social interactions and integration into the workplace community that accompanies less stable or consistent work arrangements (e.g., Cropanzano, Keplinger, Lambert, Caza, & Ashford, 2023), highlighting the importance of regular social connections for mitigating loneliness as grounded in social needs theories (Baumeister & Leary, 1995).

#### Contextual factors

Remote work (e.g., Andel et al., 2021; Mann & Holdsworth, 2003; Mann, Varey, & Button, 2000; Taser et al., 2022)^1^ and working in isolation (Cui et al., 2020; Kagan, Fridman, Shalom, & Melnikov, 2018) has also been shown to contribute to loneliness. This is vividly illustrated by studies conducted during the COVID-19 pandemic (e.g., Becker, Belkin, Tuskey, & Conroy, 2022; Geraghty, Oliver, & Wang, 2022; Zurcher, Galliker, Jacobshagen, Lüscher Mathieu, Eller, & Elfering, 2021), which found that a sudden and unprepared shift to remote work contributed to workplace loneliness. One study estimated that 63% of individuals reported higher levels of loneliness during the pandemic (Spiro et al., 2021). Additionally, limited social connections during the pandemic were associated with higher levels of loneliness (e.g., Geraghty et al., 2022; Voermans, Den Boer, Wilthagen, & Embregts, 2023), underscoring the widespread impact of COVID-19 social isolation. These findings make intuitive sense, as remote settings have been found to be associated with positive outcomes such as increased job satisfaction (Gajendran, Ponnapalli, Wang, & Javalagi, 2024), but remote work may also present challenges for forming social ties and work collaboration (Rockmann & Pratt, 2015).

Providing employees with autonomy has also been related to lower levels of loneliness. For example, Wang et al. (2021) found that jobs with more autonomy were linked to lower levels of loneliness among remote workers in China, while Poulsen and Ipsen (2017) found that when workers are instructed on when and where to work, their loneliness was higher. Relatedly, research demonstrates that psychological empowerment is associated with workplace loneliness (Kanbur & Kanbur, 2020). These findings align with the innate human need for autonomy (Ryan & Deci, 2000). When employees have control over their work environment, they are more likely to experience lower levels of loneliness because they can control the rhythms of work and rest. This allows them more bandwidth to spend time with others making connections and strengthening relationships.

Finally, job-related changes, such as relocating to another country for work, have been linked to increased loneliness. Specifically, studies involving homecare workers in Israel (Ayalon & Shiovitz-Ezra, 2010), migrant workers in Thailand (Nilvarangkul, Rungreangkulkij, & Wongprom, 2010), and migrant nurses in Australia (Joseph, Olasoji, Moss, & Cross, 2022) highlight challenges of separation from familiar support systems, leading to heightened feelings of loneliness.

#### Technology use

Another recurring theme across the studies we reviewed was the heightened risk of loneliness that is associated with the use of technology in the workplace. This is highlighted by the findings of Taser et al. (2022) and Deutrom, Katos, and Ali (2022), who demonstrated that excessive technology use hinders social relationships and is associated with loneliness. Additionally, the phenomenon of “Zoom fatigue,” as highlighted by Elbogen et al. (2022; see also Bennett, Campion, Keeler, & Keener, 2021), demonstrates how prolonged video conferencing can contribute to loneliness due to the lack of nonverbal cues and the constant self-awareness required during virtual interactions. Studies have also found a significant positive relation between cyberloafing (unproductive and excessive online behavior) and loneliness (Hu, Chen, & Ye, 2023; Yang, Lin, Chen, & Peng, 2023; Yang, Murad, Mirza, Chaudhary, & Saeed, 2022) such that more cyberloafing was related to higher levels of loneliness. There is even evidence that loneliness levels increase as employees interact more frequently with artificial intelligence systems (Tang et al., 2023). Ultimately, the impersonal nature of technological interactions may fail to fulfill individuals’ social needs (Baumeister & Leary, 1995), leading to feelings of isolation and disconnection at work.

At the same time, research reveals the paradoxical nature of technology’s role. For example, researchers point to the potential for technology to alleviate loneliness through online support groups, virtual self-help resources, and meaningful digital interactions (Bessaha, Sabbath, Morris, Malik, Scheinfeld, & Saragossi, 2020; Lim, Eres, & Vasan, 2020; Sullivan & Bendell, 2023). For example, Sullivan and Bendell (2023) demonstrated that remote workers who engage actively in online networking sites through regular posting tend to experience greater reductions in loneliness. Further, Hirshberg et al. (2022) conducted a field experiment and found that a smartphone-based meditation app helped to alleviate loneliness among public school employees.

#### Interpersonal support and friendship

Several studies have examined the extent to which personal support networks influence worker loneliness. This research is aligned with social need theory, which posits that receiving social support is central to satisfying the need to belong (Baumeister & Leary, 1995) and evolutionary theory, which posits that social interactions contribute to survival (Cacioppo & Cacioppo, 2018). For example, studies have found that higher levels of social support are directly linked to lower levels of loneliness among workers in China, Iran, and the United States (Cui et al., 2020; Shin, Park, Amano, Kwon, & Kim, 2020; Wang et al., 2021; Zamanzadeh, Jasemi, Valizadeh, Keogh, & Taleghani, 2015). Conversely, Bismark, Scurrah, Pascoe, Willis, Jain, and Smallwood (2022) found that feelings of social neglect contributed to loneliness among workers in Australia.

Family support has been found to play a similar role. Individuals with high levels of family support, as well as those who are married, report lower levels of loneliness (Gunes & Bilek, 2020; Shin et al., 2020), whereas those who are divorced/separated, living alone, or widowed report higher levels of loneliness (Bismark et al., 2022; Buecker et al., 2020; Spiro et al., 2021; Zhang & Chen, 2022). Interestingly, an increase in loneliness has also been observed following the transition to parenthood, although this effect did not hold long-term (Buecker et al., 2020). These findings are consistent with the broader literature, in which meta-analytic findings show significant negative relations with family support (Zhang & Dong, 2022). These results are also aligned with social needs theories, as familial relationships often serve as a source of fulfillment of psychological needs (e.g., Baumeister & Leary, 1995), influencing one’s experience of loneliness.

Beyond personal and family networks, social support within the organizational context also plays a crucial role in workplace loneliness. For example, greater support from coworkers and supervisors is associated with lower levels of workplace loneliness (Solomon et al., 1986; Wright et al., 2006). Similarly, Kloutsiniotis et al. (2022) found that hotel employees experienced less workplace loneliness when their managers exhibited transformational leadership (which includes providing individual consideration and support). Organizational level factors, such as perceived organizational support, also emerge as important factors of employee loneliness (Tian, Liu, & Yang, 2023; Zhou, Li, Zhou, Tao, & Bouckenooghe, 2023), suggesting that a culture of support within the workplace can yield substantial benefits in combating loneliness across various industries and sectors.

Conversely, hostile coworker behaviors are positively related to the experience of loneliness at work. Instances of peer social undermining, coworker incivility, and workplace ostracism have been identified as significant contributors to workplace loneliness, underscoring the detrimental impact of toxic workplace cultures (Cheng, Sun, Zhong, & Li, 2023; Kuriakose, Tresita, & Bishwas, 2023; Yang et al., 2022). Moreover, studies linking workplace violence to loneliness among soldiers, sex workers, and nurses highlight the need for comprehensive interventions to address the multifaceted challenges faced by vulnerable populations within the workforce (Cacioppo et al., 2016; Ding, Li, Li, Li, Xie, & Duan, 2023; Hong, Zhang, Li, Liu, & Zhou, 2013).

Finally, there is growing evidence that friendship networks play a significant role in shaping the experience of loneliness at work. For example, Cacioppo et al. (2016) found that for active duty soldiers the quality of relationships with friends was negatively related to loneliness, while Leeflang, Klein-Hesselink, and Spruit (1992) found that the size of one’s friend network was negatively related to loneliness for men in the Netherlands. Additionally, qualitative studies by Fry and Bloyce (2017) and Cardon and Arwine (2024) emphasize the importance of friendships in reducing loneliness among professional golfers and business entrepreneurs, respectively. This pattern is consistent with broader research, which shows that larger and stronger friendship networks are associated with lower levels of loneliness (see Luhmann & Hawkley, 2016, and the meta-analysis by Pinquart & Sorensen, 2001). Further, unlike work or family networks, friendships are freely chosen, which often results in stronger quality of relations (Pinquart & Sorensen, 2001). In support of this, a study by Gunes and Bilek (2020) found that employees who had friends were less lonely than people who had relatives living nearby. It would be advantageous for future research to explore differences across work, family, and friend networks in order to determine whether the voluntary nature of friendships confers an advantage in reducing workplace loneliness.

#### Summary and implications

While these studies span several countries and job sectors, they again rely on simple correlational designs, limiting our understanding of causality. For example, it is unclear whether stress leads to loneliness or whether loneliness makes individuals more susceptible to stress. Similarly, the direction of the relationship between technology and loneliness is uncertain—does increased technology use drive loneliness, or do lonely individuals turn to technology more frequently? Without longitudinal or experimental designs, these questions remain unresolved. This research also overlooks the potential of team-level dynamics. For example, the context in which teams work—such as high-pressure environments or remote settings—could exacerbate or mitigate team-wide feelings of loneliness. Technology use within teams, especially in remote or hybrid work settings, might either hinder or facilitate social connections. Finally, the piecemeal approach of this body of research restricts our ability to understand how these relationships might be moderated by factors such as individual differences, leadership styles, or work support. For example, individual levels of self-efficacy could influence how job loss impacts loneliness, with those who have higher self-efficacy potentially experiencing less loneliness due to their greater confidence in navigating social and professional challenges. Additionally, leadership styles, particularly transformational or supportive leadership, could play a critical role in mitigating the loneliness-inducing effects of technology use. Leaders who foster open communication and create opportunities for meaningful social interaction may help reduce the feelings of isolation that often accompany remote work.

### Mediators: Affective Manifestations

Cognitive discrepancy theory (Perlman & Peplau, 1981) suggests that loneliness has affective manifestations, influencing individuals’ emotions, attitudes, and feelings. Building on this, Rubenstein and Shaver (1982) identified a range of emotions such as desperation, sadness, boredom, and shame, as potential affective responses to the experience of loneliness. Research further suggests that chronically lonely individuals experience negative feelings, moods, and attitudes (Motta, 2021). We identified strain, well-being, and job attitudes as key affective outcomes in relation to loneliness and work.

#### Strain

Loneliness, like chronic stress, strains individuals’ mental and emotional capacities. Studies have found loneliness is related to affective outcomes such as emotional exhaustion (Cubitt & Burt, 2002), somatization and alienation (Soler-Gonzalez, San-Martín, Delgado-Bolton, & Vivanco, 2017), and distress (Kagan & Greenblatt Kimron, 2021; Meese, Colón-López, Singh, Burkholder, & Rogers, 2021). There is also evidence connecting loneliness and stress biomarkers. Okamura, Tsuda, and Matsuishi (2011) showed that those experiencing loneliness had heightened cortisol awakening responses (CAR) during both weekends and weekdays, as opposed to the flattened CAR experienced in nonlonely individuals on weekends. In other words, lonely individuals might not experience relief from stress during weekends, unlike their nonlonely counterparts. Similar to general loneliness, when individuals felt lonely at work, they expressed higher burnout (Bryan, Andrews, Thompson, Qualter, Matthews, & Arseneault, 2023), work-family conflict (Becker et al., 2022; Firoz & Chaudhary, 2022), and emotional exhaustion (Anand & Mishra, 2021; Becker et al., 2022; Gilmer et al., 2023).

#### Subjective well-being

Subjective well-being consists of the experience of positive affect, negative affect, and life satisfaction (Diener, 1984). There is evidence that working adults who feel lonely experience poorer well-being. For example, loneliness during COVID-19 lockdowns was related to higher negative affect among remote educators (Jelińska & Paradowski, 2021). Similarly, individuals who reported experiencing loneliness more frequently reported lower life satisfaction (Itzick & Kagan, 2021; Winefield, O’Dwyer, & Taylor, 2016; Zoch, Bächmann, & Vicari, 2022), felt that their life was less meaningful (Itzick, Kagan, & Ben-Ezra, 2018), reported lower enjoyment of daily activities (Olds et al., 2016), and overall well-being (Huang & Chan, 2022).

#### Job attitudes

Predictably, experiencing loneliness at work correlates with decreased levels of job satisfaction and other related attitudes (Basit & Nauman, 2023; Bryan et al., 2023; D’Oliveira & Persico, 2023; Gilmer et al., 2023; Moens et al., 2021; Petrovski & Gleeson, 1997). As a social stressor, loneliness is depleting and is associated with unhappiness at work. Similar effects are found for general loneliness (Phillips, 2021; Winefield et al., 2016). During the COVID-19 pandemic, general loneliness experienced in the last week was associated with job satisfaction one week later, and lower satisfaction with remote work was the mediator (Wood et al., 2023). In contrast, one study showed a paradoxically positive relationship between loneliness and job satisfaction in a sample of migrant workers (Chan & Qiu, 2011), hinting that in some contexts employees may react to loneliness by increasing their involvement with their work.

#### Summary and implications

The research evidence related to affective outcomes suggests that both feelings of persistent general loneliness and feelings of loneliness in the work context were associated with negative affective outcomes, including greater emotional exhaustion, lower job attitudes, and a general sense of poorer well-being. A key limitation of this body of work is the cross-sectional nature of the studies, which could inflate the relationships and prevent making strong inferences regarding causality, including testing the “*regulatory loop*” to assess, for example, whether loneliness leads to negative affect or whether negative affect loops back to exacerbate loneliness.^2^ Further, studies have not yet begun to adopt a multidimensional approach to the study of loneliness, and this seems particularly problematic for affective outcomes. Emotional loneliness, or the absence of intimate relationships one can rely on during difficult times, can have different implications for affective outcomes as opposed to social loneliness, or the absence of more instrumental support. Weiss (1973) proposed that emotional loneliness could be a precursor to anxiety, whereas social loneliness could result in boredom and feelings of aimlessness. The current literature’s focus on a unidimensional conceptualization of loneliness could be supplemented with studies examining the multidimensional nature of the loneliness experience.

### Mediators: Cognitive and Motivational Manifestations

In addition to its effects on emotions and attitudes, cognitive discrepancy theory highlights possible cognitive outcomes, including difficulty of concentration, being easily distracted, and learning difficulties (Perlman & Peplau, 1981). With respect to motivation, the authors identified vigor and energy, which are components of work engagement, as possible mediators. Our review highlights self-regulatory processes, self-serving cognitions, work engagement, and inefficiency as key cognitive and motivational manifestations of loneliness in working adults.

#### Self-regulation: Rumination and coping

Loneliness is an adverse experience that is accompanied by negative feelings and has been characterized as a biological signal that serves to motivate action (Cacioppo & Cacioppo, 2018). As such, loneliness is likely to influence cognitive patterns, affecting how individuals think about, conceptualize, and interpret their experiences. Gabriel et al. (2021) posited that transient feelings of loneliness would be associated with both adaptive and maladaptive cognitive processes. Specifically, they showed that the daily experience of loneliness was associated with affect-focused rumination (dwelling on the negative emotions experienced because of loneliness) and problem-solving pondering (constructive reflection on how to deal with the challenge of loneliness). In other words, they were able to connect loneliness with cognitive patterns and found that constructive, problem-solving focus had positive effects on behavioral outcomes. In another study examining how individuals adopt different cognitive patterns in relation to loneliness, Cardon and Arwine (2024) showed that some entrepreneurs experiencing loneliness adopted an emotion-focused coping style, downplaying the importance of loneliness, whereas others reported using a more problem-solving style, including changing their mindset to solve the problem or resolving to take time off from work to spend more time with others. Together, these findings provide preliminary evidence that how individuals conceptualize their loneliness in their minds will have implications for their eventual actions.

#### Self-serving cognitions

Self-serving cognitions are patterns of biased information processing that serve to protect or enhance the self (Von Hippel, Lakin, & Shakarchi, 2005). Lonely individuals can certainly benefit from engaging with others, and being more cooperative and collaborative, to alleviate their sense of loneliness. However, the regulatory loop model of loneliness suggests that loneliness can actually create a maladaptive cycle where individuals’ belongingness needs are not met, which results in the individual engaging in dysfunctional cognitions and hypervigilance against social threats, reinforcing the cycle of loneliness (Cacioppo et al., 2006; Hawkley & Cacioppo, 2010). As a result, lonely individuals tend to be more defensive, less trusting, and less supportive in their interactions with others. In the workplace, these tendencies are likely to hamper relations with coworkers. For example, in a study of employee-coworker dyads in China, workplace loneliness resulted in more self-serving cognitions, which in turn predicted coworker reports of employee territorial behavior (Nie, Chen, & Yu, 2023). Protecting one’s resources and guarding them against infringement by others is defensive behavior on the part of individuals feeling lonely at work but could serve to further isolate them from coworkers.

#### Work engagement

Evidence also suggests that individuals who feel lonely withdraw from their jobs. There is a well-established negative relationship between workplace loneliness and work engagement (e.g., Basit & Nauman, 2023; Tian et al., 2023; Yang et al., 2022), which could explain why loneliness has downstream negative effects on distal outcomes.

#### Inefficiency

Research evidence also suggests loneliness has effects on cognitive functioning and the ability to concentrate, resulting in inefficiency at work. For example, Mokros, Świtaj, Bieńkowski, Święcicki, and Sienkiewicz-Jarosz (2022) showed in a nationally representative sample of working adults that loneliness was related to physical and mental work inefficiency. This is consistent with the broader literature, which finds that loneliness is related to impaired cognitive functioning (Park et al., 2020; Yin, Lassale, Steptoe, & Cadar, 2019).

#### Summary and implications

Research examining the cognitive processes related to loneliness is in its infancy. First, it is unclear whether and when individuals who have been persistently experiencing loneliness can actually adopt a problem-focused coping style, or whether they are prone to adopting more maladaptive approaches, including rumination and mentally deciding that they do not need to address their experience of loneliness. An important next step is to identify conditions under which loneliness is accompanied by more constructive cognitive patterns. Further, there is only one study relating workplace loneliness to self-serving cognitions (Nie et al., 2023) and none examining how workplace loneliness could affect interpretations of neutral situations, increase threat appraisal in social settings, and exaggerate hostile intents of others. Instead, studies typically examined the connection between loneliness and more distal outcomes, inferring the cognitive explanations instead of directly measuring them. Finally, a key limitation of the existing body of work is the insufficient exploration of the connection between loneliness and motivation. Work engagement has been explored in numerous studies (e.g., Jung, Song, & Yoon, 2021; Stefanowski, Mokros, Sienkiewicz-Jarosz, Baka, Bugajska, & Świtaj, 2023), but there is room to adopt different motivation theories such as self-determination theory (Deci & Ryan, 1985). Moreover, research has yet to examine the interplay between affective, cognitive, and motivational mediators of loneliness, leaving an important gap in understanding how these processes interact.

### Outcomes: Behavioral Manifestations

Cognitive discrepancy theory’s third category of outcomes consists of behavioral manifestations. In our review, we identified several behavioral outcomes, and we positioned them as distal outcomes, with at least some of the relations transmitted through affective and cognitive manifestations. In this section, we discuss research relating loneliness to individual performance, social engagement behaviors, unsafe behaviors, counterproductive work behaviors, and turnover.

#### Individual performance

Workplace loneliness has been found to be negatively related to self-reported (Tian et al., 2023; Uslu, 2021) and supervisor-rated (Ghosh, 2023) job performance. Findings also indicate that entrepreneurs experiencing loneliness-induced stress report diminished productivity and performance, as well as increased venture failure (Cardon & Arwine, 2024). This is not surprising given the connection between loneliness and the affective and cognitive/motivational mechanisms described previously (e.g., negative affect, reduced work engagement).

Several mechanisms have been identified to explain why workplace loneliness is related to lower performance, but these studies have often relied on single-source data and cross-sectional methods, necessitating stronger evidence. Ozcelik and Barsade (2018) addressed this gap with a time-lagged design and multiple data sources, showing that lonely employees were less committed and were regarded as less approachable, resulting in lower supervisory performance ratings. Additional design strengths of this work include controlling for family, social, and romantic loneliness when demonstrating the effects of workplace loneliness.

Finally, evidence shows that loneliness is negatively related to workplace creativity. Peng et al. (2017) showed that workplace loneliness was negatively related to supervisor-rated creativity, mediated by lower levels of LMX quality, which captures the presence of a trust-based relationship with one’s manager. Similarly, Firoz and Chaudhary (2022) showed a negative relationship between workplace loneliness and self-rated creativity in two studies in India.

#### Social engagement

Engaging in cooperative behaviors and acting in more social ways could potentially reduce feelings of loneliness. However, there is only limited evidence for this, and it is contained in qualitative studies with small and unique samples. For example, Fry and Bloyce (2017) reported that professional golfers recounted developing friendship networks while on tour as a way to cope with loneliness. Joseph et al. (2022) interviewed migrant workers, where some indicated deliberately initiating interactions with colleagues to help cope with loneliness.

The emerging empirical evidence suggests that loneliness has primarily negative implications for the degree to which individuals act cooperatively toward others. The regulatory loop model (Cacioppo & Hawkley, 2009) explains how the perception of social situations as threatening fosters a maladaptive cycle that reduces such behaviors. Indeed, evidence reveals a negative relationship between workplace loneliness and self-rated citizenship behaviors (Andel et al., 2021; Firoz & Chaudhary, 2022). Further, studies in China found that workplace loneliness is negatively related to self-reported knowledge sharing (Cheng et al., 2023) and positively related to self-reported knowledge hoarding (Du, Ma, & Lee, 2022). Finally, Gabriel et al. (2021) showed no main effects of loneliness on next-day helping, but there were both positive and negative indirect effects on next-day helping others via problem-solving pondering and affect-focused rumination, respectively. Similarly, Cardon and Arwine (2024) showed that individuals who embraced a problem-solving approach to coping with entrepreneurial loneliness also reported making connections and engaging in activities that will allow them to connect to others in meaningful ways, whereas those using emotion-focused coping worked harder on their venture and seeking like-minded individuals online on social media. This study suggests that examining indirect effects via cognitive manifestations of loneliness may shed more light on the relation between loneliness and interpersonal behaviors.

#### Unsafe behaviors

In addition to resulting in less efficient or effective behaviors, the harmful implications of loneliness on mental exhaustion can result in unsafe behaviors. This connection is context-specific and requires sampling of safety-sensitive occupations. For example, Alroomi and Mohamed (2022a, 2022b) showed in cross-sectional studies of oil and gas field workers in Kuwait that loneliness was negatively associated with safety compliance and participation behaviors. Consistent with our conceptual model, these authors identified mental exhaustion (Alroomi & Mohamed, 2022a) and anxiety (Alroomi & Mohamed, 2022b) as partial mediators.

#### Counterproductive work behaviors (CWB)

As explained by evolutionary theory, loneliness is associated with a diminished capacity for self-regulation (Hawkley & Cacioppo, 2010), which is concerning given the importance of self-regulation for controlling oneself and focusing on tasks. Difficulty in controlling emotions can also result in CWBs. For example, Hu et al. (2023) showed that feeling lonely at work was positively related to social cyberloafing or the use of social media during work hours. Similarly, Yang et al. (2023), in a time-lagged and multisource study, showed that workplace loneliness was related to cyberloafing, and this relationship was partially mediated by ego depletion. Deutrom et al. (2022) showed that general loneliness was related to problematic internet behaviors and indirectly and negatively related to cybersecurity behaviors. Finally, a specific form of CWB is absenteeism, and even when controlling for depression, general loneliness was related to self-reported absenteeism (Mokros et al., 2022).

#### Turnover

When individuals feel lonely in general or at work, changing one’s workplace could yield positive outcomes by providing opportunities to interact with new coworkers and supervisors. In fact, research supports the idea that workplace loneliness is negatively related to organizational commitment (Jung et al., 2021; Ozcelik & Barsade, 2018), suggesting that turnover might serve as a strategic coping mechanism. However, support for this idea is mixed. Williams, Thomas, and Liao-Troth (2017) showed in their qualitative study of long-haul truck drivers that loneliness was a key influence that led drivers to leave their company or industry. Conversely, Mokros et al. (2022) found no significant relationship between general loneliness and turnover intentions among Polish adults. Further research on the implications of workplace loneliness on turnover is needed.

#### Summary and implications

The existing body of work relates workplace loneliness to numerous workplace behaviors. Most of these studies focused on loneliness that is persistent and enduring. When individuals feel chronically lonely in a work environment, the maladaptive behavioral implications of loneliness may be more likely. Further, much of the work examining loneliness in relation to workplace behaviors has utilized self-reported outcomes as opposed to more objective metrics or other-rated outcomes (for exceptions, see Jha, 2023; Lam & Lau, 2012). The evidence in relation to turnover is particularly weak due to the lack of field studies investigating this relationship. Finally, there is a need to distinguish between distinct types of loneliness, including general versus specific and emotional versus social in examination of outcomes. Ozcelik and Barsade (2018) remain the exception among studies for controlling for different types of loneliness in their examination of the effects of workplace loneliness, which is an important direction to understand the predictive power of work loneliness above and beyond general loneliness.

### Outcomes: Social and Medical Manifestations

Perlman and Peplau’s (1981) model also identified social and medical manifestations as potential outcomes, with illness and alcoholism as examples. In our review, we uncovered physical, mental health, and employment challenges among the social and medical manifestations.

#### Physical and mental health challenges

Research supports a positive link between loneliness and poor physical and mental health outcomes, with some exceptions (cf. Smith-Appelson et al., 2021). Most studies, unless otherwise specified, were cross-sectional. Research outside the scope of our review (not exclusively working adults) supports this finding and has shown that the effects of loneliness on mental health are larger than the effects of mental health on loneliness (e.g., McDowell, Meyer, Russell, Sue Brower, Lansing, & Herring, 2021). This is not surprising, as loneliness depletes individual ability to recover, thereby affecting mental and physical health. Additionally, loneliness hampers self-regulation (Hawkley & Cacioppo, 2010), which may lead to health-compromising behaviors. For example, studies have found that loneliness among immigrants is related to increased alcohol consumption, unsafe sexual practices (Negi et al., 2021), and elevated smoking rates (Kong, Chen, & Cheng, 2023).

Researchers have paid particular attention to the effects of general loneliness on psychological and mental health among marginalized groups and socially isolated occupations, oncology nurses (Phillips, 2021), migrant workers (Ayalon & Shiovitz-Ezra, 2010; Di Napoli et al., 2021; Regmi, Aryal, van Teijlingen, Simkhada, & Adhikary, 2020), active-duty soldiers (Trachik et al., 2022), and firefighters (Stefanowski et al., 2023). For example, Gjerstad et al. (2020) showed that former peacekeepers who recalled feeling lonely during their deployment 30 years ago reported higher current post-traumatic stress symptoms, suggesting the possibility that loneliness may contribute to PTSD and make recovery more difficult. Physical health outcomes included somatic symptoms such as pain and gastrointestinal symptoms (Tam, Zhou, Qiao, Li, & Shen, 2022) and self-reported general health complaints such as headaches (Leeflang et al., 1992).

The effects of loneliness on physical and mental health (Kuriakose, Sreejesh, Wilson, & Anusree, 2019; Kuriakose et al., 2023; Yang et al., 2022) generalizes to transient forms of loneliness as well. For example, a weekly diary study conducted by Andel et al. (2021) found that workplace loneliness predicted weekly depression. Additionally, daily feelings of loneliness predicted after-hours insomnia and alcohol use (Tang et al., 2023). Not surprisingly, physical and mental health emerged as key outcomes during the COVID-19 pandemic. Loneliness was related to depressive symptoms among working adults (Blomqvist et al., 2023), remote workers (Becker et al., 2022; Elbogen et al., 2022; Şentürk, Sağaltıcı, Geniş, & Toker, 2021), and poorer mental health outcomes among health care professionals (Anzola et al., 2022; Kotera, Ozaki, Miyatake, Tsunetoshi, Nishikawa, & Tanimoto, 2021). In a national sample, Wanberg et al. (2020) showed that increases in loneliness during COVID-19 were related to increases in depressive symptoms. Further, pandemic-related loneliness was tied to receiving professional support for mental health (Lin, 2023) and suicidal ideation (Sasaki, Kuroda, Tsuno, Imamura, & Kawakami, 2021). Loneliness during lockdowns was linked to COVID-related worries and indirectly related to anxiety, depression, and insomnia (Megalakaki & Kokou-Kpolou, 2022).

#### Employment challenges

Given the health risks associated with loneliness, emerging evidence suggests it may also increase the risk of unemployment. For example, a study of more than 10,000 older adults working in 14 European countries found that loneliness was linked to depression two years later, which in turn was related to the onset of a work disability due to health problems (Morris, 2020). Morrish, Mujica-Mota, and Medina-Lara (2022) utilized data from two waves of a UK population survey and found that loneliness increased the probability of unemployment by 17.5%. This relationship was stronger among the permanently sick or disabled. Factors such as depression, poorer physical health, emotional exhaustion, or limited social networks likely contribute to this relationship. Finally, loneliness has been related to higher job insecurity among employed individuals (Zhou et al., 2023), suggesting that even among the employed, loneliness may pose challenges to employment.

#### Summary and implications

When it comes to social and medical manifestations, researchers focused on general, as opposed to workplace, loneliness. This body of work suggests that persistent feelings of being lonely in one’s life are a health risk and contribute to insecure employment. It is unclear whether workplace loneliness has similar effects, or whether any effects of workplace loneliness on health and employment outcomes are mediated by general loneliness. The effects of accumulated loneliness on health may differ from temporary feelings experienced in a single domain such as work. Therefore, explicit consideration of the chronicity and domain of loneliness seems important. Moreover, the behavioral and social/medical outcomes of loneliness could be interconnected. For example, loneliness could be harmful to one’s health, affecting their performance at work. Alternatively, loneliness could result in poor performance outcomes, resulting in involuntary turnover, which could be a precursor to poor health outcomes.

### Cross-level Influences

Integrating cognitive discrepancy theory (Perlman & Peplau, 1981) with the affect theory of social exchange (Lawler, 2001) suggests that the loneliness of one person has the ability to influence the attitudes and behaviors of another person, or that the collective levels of loneliness experienced within a group has the potential to influence individuals or groups. Thus, we discuss the effects of leader and team loneliness as higher-level manifestations of loneliness.

#### Effects of leader loneliness

Studies have examined within and cross-level effects of leader loneliness on employees, groups, and organizations (Lam et al., 2024). Replicating the studies on individual loneliness, studies on leader loneliness show that feelings of loneliness among leaders contributed to their own poor mental and physical health (Cooper & Quick, 2003) but also had implications for employees reporting to them. Specifically, when leaders are distressed because they are dissatisfied with the quality and quantity of their work relationships, they have less to offer their employees. Supporting this prediction, Chen, Peng, Lei, and Zou (2021) found that leader loneliness was related to reduced cognitive trust in one’s leader, which contributed to turnover among 133 teams in China. In a study examining both employee and leader loneliness, researchers showed that the highest levels of LMX quality was observed when leaders and members both had low levels of workplace loneliness, whereas having leaders report greater levels of loneliness relative to employees was associated with lower-quality LMX, contributing to turnover intentions (Chen et al., 2016). Finally, Gabriel et al. (2021) examined how daily loneliness among leaders could affect their leadership toward subordinates. While this was not a cross-level study, they showed that leader loneliness was related to lower follower reports of empowering leadership and leadership effectiveness, demonstrating effects on subordinates. Thus, we see value in future research examining this relationship in terms of how leader loneliness affects subordinates and vice versa.

#### Effects of team loneliness

Recently, there have also been efforts to move workplace loneliness research beyond the individual level to the team level by exploring the effects of a loneliness climate within the group. The affect theory of social exchange proposes that individuals may experience shared emotions such that individual emotions become collective emotions (Lawler et al., 2014). Operationalizing this idea, Chen et al. (2021) showed that the average level of individual loneliness in the group interacted with leader loneliness to predict employees’ trust in the leader, with leader loneliness having more harmful effects on trust in the leader when average loneliness in the team is high. Further, utilizing a referent-shift approach, Yang and Wen (2021) asked individuals to assess how lonely their team felt and showed that loneliness climate was negatively related to supervisor ratings of team performance. These preliminary findings suggest that team loneliness can have cross-level, as well as group-level, effects.

#### Summary and implications

The idea that the loneliness of one individual can have implications for others interacting with that individual is an important one, and additional work examining team-level loneliness and its associated outcomes (team-level cooperative behaviors and citizenship behaviors) is needed. Leader loneliness is one example of how their reactions to their own loneliness could affect their interactions with peers and subordinates, influencing resource distribution, the type of leadership style employees are exposed to, and broader job attitudes of employees. The idea that loneliness can be a collective phenomenon at work is also important due to the possibility of loneliness contagion (Cacioppo, Fowler, & Christakis, 2009), and we still know little about how loneliness at work is transmitted to others and how it influences individuals and teams.

### Moderators of the Effects of Loneliness

While loneliness is associated with negative outcomes such as poorer well-being, lower levels of job satisfaction, effectiveness, and coworker relationships, our review also highlights potential moderators that can buffer (alleviate) the harmful effects of workplace loneliness.

#### Individual differences

Individuals may have psychological, financial, material, or health resources that could aid in coping with the effects of loneliness. For example, Megalakaki and Kokou-Kpolou (2022) showed that during COVID-19, the harmful effects of general loneliness on mental health outcomes were stronger for those who had lost their jobs, those living alone, those with pre-existing health issues, and those who were worried about COVID-19. In a qualitative study of adults who suffered from chronic pain, participants identified having a job as a resource that helped them cope with their general loneliness (Nilsen & Anderssen, 2014).

With respect to psychological resources, research has identified personality traits that can enable individuals to cope with feeling lonely and shield them from the negative consequences. These traits buffer the negative effects of loneliness and include self-compassion (Andel et al., 2021), psychological capital (Firoz & Chaudhary, 2022), self-efficacy (Gabriel et al., 2021), and need for belongingness (Basit & Nauman, 2023). In contrast, loneliness had stronger negative effects on extraverts (Tian et al., 2023) and women (Tian et al., 2021). Personality differences may also weaken the loneliness-behavior relationship by compelling individuals to display the behavior regardless of feeling lonely. For example, Hu et al. (2023) found a positive relationship between workplace loneliness and social cyberloafing only for those low in conscientiousness.

#### Leadership

Leader behaviors mitigate some of the harmful effects of workplace loneliness. Peng et al. (2017) showed that leader compassion alleviated the indirect negative effects of workplace loneliness on supervisor-rated creativity, while Jin and Ikeda (2023) found that servant leadership reduces employee loneliness by enhancing empathic communication. Similarly, Nie et al. (2023) showed that self-sacrificial leadership weakened the positive relationship between workplace loneliness and self-serving cognitions. Yang et al. (2023) identified leader problem-focused emotion management as a behavior that neutralized the harmful effects of workplace loneliness on employee ego depletion. Interestingly and unexpectedly, Anand and Mishra (2021) found that LMX moderated workplace loneliness and emotional exhaustion relationship such that the positive relationship was stronger when LMX was higher. This relationship may be due to backlash toward in-group members, but further exploration of this relationship is warranted.

#### Work context

The work context can also play a role in determining the effects of workplace loneliness. Supportive climates seem to buffer the harmful effects of workplace loneliness, whereas climates characterized by anger and competition can strengthen the harmful effects (Du et al., 2022; Ozcelik & Barsade, 2018). High-quality relationships with coworkers also serve as protective factors mitigating the adverse outcomes associated with loneliness (Jung et al., 2021). Conversely, coworker loneliness can amplify the detrimental effects of loneliness on affective commitment (Ozcelik & Barsade, 2018). Moreover, situational strength can buffer the adverse effects of workplace loneliness on workplace outcomes, such that when the situation is strong, loneliness is a less powerful behavioral predictor. As an example, Cheng et al. (2023) found that high task interdependence, which necessitates regular knowledge sharing, weakened the negative relationship between workplace loneliness and self-reported knowledge sharing. These findings highlight the necessity of fostering supportive and structured work environments to mitigate the impacts of workplace loneliness.

#### Summary and implications

Research on moderators suggests that loneliness is not equally detrimental for everyone, and there are individual differences as well as organizational offerings such as leadership and a supportive culture that can weaken the harmful effects of loneliness. We believe that there is room to investigate moderators further, particularly focusing on the role of chronicity of loneliness in determining reactions to it. Similarly, individuals who feel lonely only in the work domain may have different reactions to loneliness as opposed to individuals who feel lonely across different domains such as friendships and family. Identification of moderators is likely to remain an important area for further research. Finally, the effects of loneliness on outcomes could be contingent on the type of loneliness being experienced (social versus emotional) and contrasting the effects of general loneliness to the effects of these subdimensions remains important.

### Loneliness Interventions

There are only a small number of studies examining interventions, but the results from these studies are informative. Our review highlights four interventions that are particularly effective in mitigating workplace loneliness: stress management, social skills, volunteering, and mindfulness.

#### Stress management

The provision of social support has also been shown to significantly reduce loneliness, particularly among specific groups predisposed to experience high loneliness. Bessaha et al. (2020) reviewed interventions primarily targeting clinical populations, such as individuals with illnesses or disabilities, caregivers, refugees, and immigrants. Their review found that support group interventions, including techniques like online support, peer mentoring, and group support, were particularly effective in reducing loneliness. Additionally, studies focusing on military personnel post-9/11 demonstrated that small group training sessions targeting personal stress-management techniques were effective (Williams, Hagerty, Yousha, Horrocks, Hoyle, & Liu, 2004).

Future research could adopt a resource-based view, wherein loneliness is framed as a lack of personal resources in the form of meaningful social connections (Torrès, Benzari, Fisch, Mukerjee, Swalhi, & Thurik, 2022). This perspective enables researchers to draw on stress scholarship to identify ways workers can regenerate resources and reduce loneliness proactively. Viewing loneliness as a resource-draining experience, researchers could leverage conservation of resources theory (Hobfoll, 1989) to discover methods through which workers can conserve their remaining resources and manage the negative personal implications of loneliness. However, focusing on personal outcomes may impact productivity, potentially leading to increased loneliness as coworkers respond adversely to diminished productivity (Cacioppo et al., 2006; Hawkley & Cacioppo, 2010).

#### Social skills

Building interpersonal skills is another critical intervention for alleviating workplace loneliness. Cacioppo et al. (2015) highlighted that small group training sessions focused on enhancing interpersonal skills significantly reduced loneliness among military personnel. The effectiveness of such interventions underscores the importance of fostering strong social connections within the workplace. Strong relationships with coworkers can serve as protective factors against loneliness, emphasizing the need for social skills development in employee training programs.

#### Volunteering

Volunteering has also been identified as a valuable strategy for reducing loneliness. Matthieu, Lawrence, and Robertson-Blackmore (2017) found that military personnel who spent 20 hours per week volunteering for a nonprofit organization reported lower levels of loneliness. This finding indicates that engaging in meaningful activities outside of work can help mitigate feelings of isolation and enhance social connectedness. Encouraging employees to participate in volunteer work can be an effective component of comprehensive loneliness intervention programs.

#### Mindfulness

There is also evidence that mindfulness shows promise in addressing loneliness among working adults. In the broader loneliness literature, researchers have found that the most effective interventions were those targeting the maladaptive cognitions arising from loneliness, such as the negative self-talk and recurring negative thoughts about others (Cacioppo et al., 2015). Mindfulness, by encouraging individuals to stay in the moment, may disrupt these cognitive patterns. Hirshberg et al. (2022) conducted an experiment with full-time employees in the education sector and found that a mindfulness app significantly reduced loneliness, with effects persisting over a three-month period indicating that technology is paradoxical in that it can both help and hurt in terms of loneliness. Similarly, Sylvia et al. (2021) implemented a stress training program for clinicians that incorporated mindfulness and cognitive-behavioral therapy, resulting in a significant reduction in loneliness. Interestingly, LeCheminant, Merrill, and Masterson (2017) found that a general wellness program didn’t affect loneliness, suggesting that interventions specifically focused on meditation and mindfulness may be more effective than broader health programs.

#### Summary and implications

The body of research on workplace loneliness interventions is still emerging, but existing studies suggest that these four interventions show strong potential to mitigate loneliness. This is further supported by findings from the broader nonwork literature (e.g., Bessaha et al., 2020; Hicken et al., 2021; Warner et al., 2024; Zhang & Dong, 2022), which further emphasizes the effectiveness of group-based interventions such as hobby groups, faith-based groups, and social clubs (Morrish, Choudhury, & Medina-Lara, 2023). Additionally, Hadley and Wright (2024) offer several suggestions for new approaches to combatting loneliness at work. However, addressing work-related loneliness is unlikely to have a “one-size-fits-all” solution, especially in settings where coworkers do not work in the same physical location.

## Future Research Directions

To fully understand the dynamics of loneliness and work, it is essential for future research to broaden its scope and deepen its exploration of this complex phenomenon. Our conceptual model offers a robust theoretical and empirical foundation to guide this work, and we have identified four major categories for future inquiry: the nature of loneliness, the nomological network of loneliness, the conceptual and social dynamics of loneliness, and loneliness interventions. Table 3 outlines these categories and further breaks them down into specific research questions and opportunities, providing a structured roadmap for examining the intricate relationships between loneliness, work environments, and individual/team experiences.

**Table 3:** Critical Observations and Future Research Questions

Focus Area	Themes, Topics, and Core Questions
The Nature of Loneliness	
**Conceptual Advances** • Work that uncovers the complexity inherent in the experience of loneliness is needed.	Social vs. Emotional Loneliness: • Are there differences in intensity between **social and emotional loneliness**? For example, **emotional loneliness** may trigger stronger physiological responses due to its connection to personal attachment. **Social loneliness** may be less intense but prolonged. • Are the antecedents (**predisposing** [e.g., personality] and **precipitating factors** [e.g., stress]) or outcomes (**affective, cognitive/motivational, behavioral, social/medical)** different for these types of loneliness? For example, neuroticism may predispose individuals to experience emotional loneliness due to difficulty forming intimate relationships, while introversion may be more relevant for social loneliness because it is related to the capacity to join social networks. Threshold/Intensity of loneliness • What is the “*threshold of deficiency*” of desired versus experienced connection that could trigger **loneliness**? How much of a gap (in terms of time, quality, or type of connection) is tolerated before loneliness sets in? • What **predisposing** or **precipitating** factors can cause shifts in the threshold over time? For example, neuroticism may lower the threshold for loneliness, whereas extroverted individuals may tolerate larger gaps before loneliness occurs.
**Chronicity of Loneliness** • The majority of studies focus on more chronic, persistent forms of loneliness. We need research on situational, statelike loneliness.	Chronic vs. Transient Loneliness • Could **chronic loneliness** be more strongly associated with *maladaptive* **self-regulation** strategies (e.g., surface acting, emotion suppression), and **transient loneliness** with *adaptive* **self-regulation** strategies (e.g., problem-focused pondering)? Chronic loneliness may lead to **emotion regulation** (suppression), while transient loneliness may prompt **work engagement**. • Could the potentially **transient** nature of **loneliness** explain mixed findings? For example, research comparing gender differences in loneliness may be resolved by uncovering how loneliness fluctuates daily, weekly, or even annually. Loneliness Profiles • Are there **loneliness** “profiles” where individuals can be grouped into distinct classes? Are there profiles based on one’s **predisposing factors**, at what rate does state-like loneliness fluctuate, and how do the profiles respond to targeted interventions?
**Methodological Considerations** • Few studies have used time-lagged, longitudinal, experimental, or multi-source designs, resulting in opportunities to increase the methodological rigor.	Advanced Methodological Designs • Adopt more sophisticated methodological designs, including studies that are longitudinal, time-lagged, experimental, adopt ESM, and incorporate data from employees, leaders, coworkers, and family members or friends. • Does **loneliness** lead to negative affect or does negative affect lead to **loneliness**? For example, testing the regulatory loop may show that both occur, and this creates a loneliness-negative affect spiral. Measurement • How might self- vs. other-reported **loneliness** differ? For example, comparisons of self- vs. other-reports of loneliness may uncover effective and ineffective self-presentational strategies adopted by those experiencing loneliness. • Under what conditions are individuals more likely to perceive other people’s loneliness? For example, assessing other people’s perceptions of a colleague may highlight currently underrecognized behavioral and linguistic indictors of loneliness. • Develop a generalizable instrument to assess **loneliness** that encompasses the six core elements we identify in this paper.
Nomological Network of Loneliness	
**Integration across Topics** • Research has been piecemeal. Research that integrates across constructs, domains, and categories is needed.	The Experience of Loneliness • What is the relation between **stress/strain** and **loneliness**? Under what conditions does **stress** as a precipitating factor lead to loneliness and to **strain** (as a mediator), and under what conditions does **strain** lead to **loneliness** and to **stress**? • To what extent is **loneliness** related to core motivational needs beyond relatedness, such as the need for autonomy or competence? A work environment with a lack of autonomy and a leader that reduces one’s perceived competence may exacerbate loneliness by limiting individuals’ control over social engagement and undermining their confidence in professional settings, respectively. Cross-Domain Effects • How might experiences of **loneliness** in one domain (work) spill over into the other domains (nonwork or vice versa)? Workplace loneliness may spill over into personal relationships, compounding feelings of disconnection, while nonwork loneliness can similarly hinder forming connections at work. • How might **loneliness** appear at the **team level**? Using a referent-shift measurement approach, we can capture the existence and effects of a workplace loneliness climate. Interaction Effects/Moderators • In what ways do **predisposing** and **precipitating factors** interact to influence **loneliness**? For example, does **personality** interact with **contextual factors** (work environment) to influence loneliness? An introvert may find it more challenging to engage in social interactions in a collaborative work setting, providing insight into how to create supportive work cultures to reduce loneliness. • Does **leadership style** moderate relations between **predisposing** and **precipitating factors** and loneliness? For example, can leadership support (LMX) serve as a substitute for **interpersonal support** from coworkers (CWX)? Leader support may buffer a lack of peer support in environments where coworker relations are weak.
**Mechanisms of Loneliness** • A number of mechanisms have been advanced to explain the relation between loneliness and outcomes but empirical research is lagging.	Mechanisms • How are the **affective, cognitive**, and **motivational** mechanisms interconnected and what is their relative influence within the regulatory loop? Specifically, is the impact of loneliness on these mechanisms greater than their reciprocal influence on loneliness? • How do lonely people utilize **emotion regulation** strategies? Understanding the regulation strategies used by lonely people could provide insights into designing interventions aimed at improving emotional well-being and reducing loneliness. Potential Biases • How do the cognitive biases advanced by regulatory loop theory, and the emotional biases advanced by affect theories, explain the experience of **loneliness**? A bias toward negative affect may exacerbate a worker’s loneliness as they attend to negative information from coworkers and leaders. Alternatively, coworkers may blame the individual for their **loneliness** rather than considering **contextual factors** (fundamental attribution error), thus influencing how they interact with a lonely colleague.
**Adaptive Loneliness** • Theory and research highlight the potential for an adaptive side of loneliness but research on this topic is lacking.	Facilitative Loneliness • Explore the “lone wolf” hypothesis, which suggests that some individuals may thrive when alone (Cardon & Arwine, 2024) to see if **loneliness** can enable employees to have higher **work engagement** and productivity. By reducing the need to engage in social dynamics, loneliness might foster heightened autonomy, allowing employees to focus on outcomes and long-term goals. Self-Regulation and Loneliness • What **self-regulatory** mechanisms could foster adaptive levels of **loneliness**? For example, cognitive reappraisal (reframing loneliness as an opportunity for reflection) and goal-setting (directing energy toward productive tasks) could be effective.
Contextual and Social Dynamics of Loneliness	
**Remote Work, Technology, and Loneliness** • Remote work and the use of advanced technology are the norm but their impact on loneliness is complex and understudied.	Remote Work • Does the relation between remote work (**contextual factor**) and **loneliness** change as a function of a worker’s preference to work remotely? Employees who prefer remote work may be less lonely, and this could be moderated by factors such as age (**individual differences**) and whether their tasks are collaborative/independent (**work context**). Younger workers with independent tasks may fare worse because they crave networking opportunities that are limited in remote work. Technology • Does the use of AI **technology** (e.g., GPT-4o) for human-computer collaborative tasks, such as team projects or brainstorming sessions, reduce **loneliness** by enhancing communication and providing a sense of virtual presence, or does it inadvertently exacerbate loneliness by replacing human interaction? • Does computer-mediated communication at work differently predict **loneliness** from face-to-face communication? Computer-mediated communication offers convenience and flexibility but lacks the emotional richness of face-to-face interactions, potentially leading to higher loneliness.
**Vulnerable Populations and Loneliness** • Research on vulnerable populations (e.g., ethnic minorities, LGBTQ+, people with disabilities) at work is largely unexplored, highlighting a gap in understanding how marginalized groups are uniquely impacted.	Equity, Diversity, and Inclusion • How does workplace social rejection for vulnerable populations (**demographics**) relate to **loneliness** and under what conditions is that relationship attenuated? Social rejection can heighten loneliness for vulnerable groups, but this may be reduced when support systems (**interpersonal support, leadership**) and inclusion (**contextual factors**) strategies are present. • Do existing **interventions** work with vulnerable populations? Vulnerable populations may experience loneliness differently and face additional barriers to support, thus interventions may not fully address the unique needs of these groups. Vulnerable populations may also have distinct **social** and **emotional** needs that require tailored approaches. Interpersonal Support and Friendship • How do we strengthen the relationship between **interpersonal support** and friendships at work and worker **loneliness** for vulnerable populations? Vulnerable populations (e.g., ethnic minorities, LGBTQ+, employees with disabilities) may experience barriers to accessing meaningful social support at work. Exploring factors such as reciprocity and trust in relation to this question is important.
Overcoming Loneliness: Interventions	
**Loneliness Interventions** • There is some work on interventions that focus on stress management, social skills, volunteering, and mindfulness but lots of room for additional research.	Combined Interventions • What combination of **interventions** could be used to provide deeper insights into creating comprehensive and tailored approaches to combat loneliness in the workplace? Identifying a mix of interventions could offer a more personalized approach to reducing loneliness, which is particularly important in diverse work environments. • Are some interventions more effective at addressing cross-domain loneliness than others? Work Design • What is the value of autonomy-enhancing **interventions** aimed at reducing loneliness among employees? Autonomy-enhancing programs may empower employees to manage their tasks and social needs more effectively. • How could work be designed in alignment with the job characteristics model (Hackman & Oldham, 1976) to increase opportunities for social interaction and direct contact with colleagues? Designing jobs to incorporate **cooperative behaviors**, **interpersonal support**, and interdependent tasks (**work context**) could help reduce loneliness.

*Note.* **Boldface** type aligns with components of our model from Figure 1.

The first category, *the Nature of Loneliness*, focuses on uncovering the complexity inherent in workplace loneliness and recognizes it as a multifaceted and dynamic phenomenon. This category emphasizes distinctions between social and emotional loneliness, thresholds that trigger loneliness, and differences between chronic and transient states of work-related loneliness. It further emphasizes the importance of advancing methodological rigor and refining measurement tools to deepen insights into how loneliness manifests. Our second category, the *Nomological Network of Loneliness*, aims to expand this understanding by integrating related constructs and exploring mechanisms, moderators, and cross-domain effects, such as the relation between stress and loneliness, the interplay between workplace loneliness and personality, and the extent to which cognitive biases may help to explain the experience of loneliness. This category also acknowledges the possibility of an adaptive side to loneliness, wherein loneliness might foster increased autonomy, focus, and goal-driven behaviors. Investigating how loneliness may positively influence work engagement or productivity offers a nuanced perspective on its potential benefits.

The third category, the *Contextual and Social Dynamics of Loneliness*, examines modern workplace influences, such as remote work, technology, and the unique vulnerabilities faced by marginalized groups, emphasizing the need for equity, diversity, and inclusion strategies. For example, we propose that remote work may exacerbate loneliness for employees who thrive on in-person interactions but reduce it for those who prefer working independently. Similarly, the use of AI tools for collaboration may enhance virtual presence and reduce loneliness for some, while limiting human interaction and deepening feelings of isolation for others. These dynamics highlight the importance of understanding individual and contextual factors to develop targeted strategies for mitigating loneliness. Finally, our category called *Loneliness Interventions*, explores actionable solutions for mitigating the potentially harmful effects of workplace loneliness, including combined intervention strategies, autonomy-enhancing work designs, and initiatives to strengthen interpersonal support. These interventions underscore the need for thoughtful, evidence-based strategies to address loneliness effectively at both individual and organizational levels.

## Guidelines for Practitioners

Given the relatively early stage of the literature on loneliness and work, it is clear that there is much to be discovered about effectively mitigating loneliness in the workplace. Our review of relevant studies highlighted four promising strategies for practitioners: implementing interventions that focus on stress management, social skills building, volunteering, and mindfulness. These interventions can be enhanced in four ways: by using technology, by offering robust social and psychological support, by fostering workplace friendships, and by cultivating a compassionate organizational culture.

First, there is growing evidence that the integration of AI into loneliness interventions is promising and might greatly benefit practitioner efforts. For example, the use of personal voice assistants (Jones, Hanus, Yan, Shade, Blaskewicz Boron, & Maschieri Bicudo, 2021) and chatbots (De Gennaro, Krumhuber, & Lucas, 2020) were shown to be related to reduced levels of loneliness. Such AI-powered solutions have the potential to analyze and respond to individual preferences, communication patterns, and work habits to provide tailored strategies to mitigate loneliness. In a similar vein, recently introduced AI-driven advanced language models, such as chatGPT, offer multimodal and human-like interactions that can amplify the efficacy of interventions in combating loneliness in the workplace. However, these interventions should be approached with caution. While they offer benefits like accessibility and cost reduction, they likely cannot replace the emotional depth of human interaction and whether they help individuals develop social skills to address their own loneliness is yet to be seen, which are key to addressing loneliness. Additionally, AI raises data privacy and security concerns that must be carefully managed.

There is also evidence that providing support to employees is negatively related to feelings of loneliness. For example, psychological support (Bennett et al., 2022), social support (Cui et al., 2020; Shin et al., 2020), and employee networks such as LGBTQ+ communities (McFadden & Crowley-Henry, 2018) contribute to the reduction of loneliness. Mentorship programs, especially relevant for employees who joined organizations during the COVID-19 pandemic, also provide crucial avenues for support (Dor-Haim & Oplatka, 2021; Saks & Gruman, 2021). Indeed, research shows that feelings of belonging are critical for success during new employee socialization (Bauer, Erdogan, Ellis, Truxillo, Brady, & Bodner, 2025). As such, practitioners are encouraged to facilitate meaningful interactions among coworkers through mentoring initiatives, diversity programs, team-building activities, and the provision of mental health support. Further, in line with past research (e.g., Lam & Lau, 2012), programs aimed at enhancing LMX and coworker exchange (CWX) are likely to be beneficial. This could be accomplished by ensuring clear communication, regular constructive feedback, individualized attention, and fair practices (Bauer & Erdogan, 2015; Sullivan & Bendell, 2023).

Fostering workplace friendships is another avenue to address loneliness, with a growing body of research showing that friendships can significantly mitigate loneliness in work contexts (e.g., Cacioppo et al., 2016; Leeflang et al., 1992). Workplace loneliness often stems from environments lacking opportunities for genuine connection, and organizational cultures that neglect these connections risk exacerbating loneliness, particularly when toxic behaviors, like exclusion and cliques, undermine social bonds (Hadley & Wright, 2024). To address this, organizations can embed social activities into the rhythm of work—whether through informal conversations, communal lunches, or team-building initiatives. Such efforts may not only alleviate loneliness but also enhance organizational outcomes by creating an inclusive culture where friendships thrive and employees feel valued and engaged. Finally, cultivating a compassionate organizational environment is likely to help alleviate loneliness as it pertains to work. Practices such as compassionate leadership (e.g., Peng et al., 2017), voluntary community involvement (Carr, Lennox Kail, Matz-Costa, & Shavit, 2018; Lee, 2022; Wilson-Forsberg & Sethi, 2015), and fostering self-compassion (e.g., Andel et al., 2021; Kotera et al., 2021) have shown positive relations with the reduction of loneliness. Organizational practitioners should consider establishing training programs focused on compassionate leadership (e.g., Paakkanen, Martela, Hakanen, Uusitalo, & Pessi, 2021) and endorsing employer-supported community involvement practices. As the field continues to evolve, it is imperative that organizations remain adaptable and proactive in implementing these strategies. By fostering a culture that prioritizes connection and empathy, organizations can enhance employee well-being, boost morale, and ultimately drive productivity and organizational success. Future research should continue to explore and refine these approaches, ensuring they are adaptable to various work settings and responsive to the diverse needs of the workforce.

## Conclusion

Exploring the intersection of loneliness and work is imperative in light of the contemporary epidemic of loneliness permeating our global landscape. Our review highlights the extensive insights that the field has gained about work and loneliness, while revealing the complexities involved in operationalizing loneliness in work contexts, how we frame distinct dimensions of loneliness, and how loneliness interacts with other variables to influence our work-related attitudes and behaviors. By synthesizing theoretical frameworks and empirical findings from hundreds of studies, we have advanced a robust conceptual model of loneliness and work that integrates cognitive discrepancy theory (Peplau & Perlman, 1982), the affect theory of social exchange (Lawler, 2001), and evolutionary theory (Cacioppo & Cacioppo, 2018). In alignment with cognitive discrepancy theory, our model identifies predisposing and precipitating factors as antecedents, such as personality traits and job-related stressors. Affective and cognitive/motivational factors mediate these relationships in a regulatory loop, in line with evolutionary theory (Cacioppo & Hawkley, 2009). Downstream, the model delineates distal outcomes encompassing both behavioral and social/medical dimensions, such as individual performance and mental health issues. Moreover, consistent with the affect theory of social exchange (Lawler, 2001), our model addresses cross-level influences within teams and among leaders. It also incorporates moderators like individual differences and work context variables and suggests interventions including stress management and mindfulness practices. Combined, our comprehensive framework synthesizes past findings and advances theoretical understanding, providing a cohesive framework for future research.

Our model also offers actionable insights for future research and practitioners. Table 3 identifies promising research opportunities, including conceptual advances, methodological considerations, interventions, and the potential positive aspects of loneliness. Our review also provides practitioners with tangible recommendations, from implementing technology-enhanced loneliness interventions to fostering supportive organizational environments. These insights are designed to inform practical strategies that address the pressing challenges of workplace loneliness. Continued exploration in this area will further unravel the complexities of work and loneliness, illuminating pathways to meaningful connections, reducing loneliness, and promoting employee well-being and resilience. Through these efforts, we can foster organizational environments that enhance social connectedness and employee health. Ultimately, by addressing loneliness in the workplace, we can cultivate more connected, resilient, and thriving organizational cultures that drive sustained success and well-being.

## Supplemental Material

sj-docx-1-jom-10.1177_01492063241313320 – Supplemental material for All the Lonely People: An Integrated Review and Research Agenda on Work and Loneliness Supplemental material, sj-docx-1-jom-10.1177_01492063241313320 for All the Lonely People: An Integrated Review and Research Agenda on Work and Loneliness by Julie M. McCarthy, Berrin Erdogan, Talya N. Bauer, Selin Kudret and Emily Campion in Journal of Management

## Notes

1. A notable exception is a cross-sectional study conducted by Zurcher et al. (2021), which found that 75% of academic counselors in Switzerland said they did not feel lonely when working from home.

2. Research that tests the “regulatory loop” with respect to cognitive and motivational mediators is also needed.
