# Supplementary material for: All the Lonely People: An Integrated Review and Research Agenda on Work and Loneliness
Source: J Manage. 2025 Feb 14;52(1):283–330. doi: 10.1177/01492063241313320 (PMC12701760; doi:10.1177/01492063241313320)
Supplement: sj-docx-1-jom-10.1177_01492063241313320 – Supplemental material for All the Lonely People: An Integrated Review and Research Agenda on Work and Loneliness [file sj-docx-1-jom-10.1177_01492063241313320.docx]

SUPPLEMENTAL MATERIALS

1. **Table S1.** Summary of the 213 papers (reporting on 236 studies, 233 which were empirical) at the Intersection of Work

and Loneliness 2

1. **Table S2.** Review of 213 Papers (reporting on 236 Studies, 233 which were empirical) at the Intersection of Work

and Loneliness 3

1. **Table S3.** Definitions of Loneliness 24
2. **Table S4.** Summary of Scales Used in Loneliness Research. 27
3. **Figure F1.** Prisma Figure of Literature Identification 30

**Table S1. Summary of the 213 papers (reporting on 236 studies, 233 which were empirical) at the Intersection of Work and Loneliness**

| **Category** | **Type** | **Number of Studies** | **Percentage (out of 236)** |
| --- | --- | --- | --- |
| Study Sample | Exclusively working adults | 182 | 77.1% |
|  | Not exclusively working adults | 51 | 21.6% |
|  | No sample (conceptual) | 3 | 1.3% |
| Study Design | Cross-Sectional | 130 | 55.0% |
|  | Qualitative | 45 | 19.1% |
|  | Time-lagged | 40 | 16.9% |
|  | Experimental | 12 | 5.1% |
|  | Conceptual/Theoretical | 3 | 1.3% |
|  | ESM | 3 | 1.3% |
|  | Meta-Analysis | 3 | 1.3% |
| Participants | Single Source | 215 | 91.1% |
|  | Multiple Source | 16 | 6.8% |
|  | No sample/Meta-Analysis | 5 | 2.1% |
| Countries/Regions/ Territories (*N*=40) | Argentina, Australia, Belgium, Canada, Chile, China, Denmark, France, Germany, Greece, Hong Kong, India, Indonesia, Ireland, Israel, Italy, Japan, South Korea, Kuwait, Laos, Macao, Malaysia, Mexico, Nepal, Netherlands, New Zealand, Nigeria, Norway, Pakistan, Philippines, Poland, Portugal, South Africa, Spain, Sweden, Switzerland, Taiwan,  Turkey, United Kingdom, United States. | | |
| Measures | UCLA scale (scales by Hughes et al. (2004), Russell et al. (1980), or adaptations of these scales) | 83 | 39.0% |
|  | Qualitative | 44 | 20.7% |
|  | Single item measures | 25 | 11.7% |
|  | Workplace loneliness (Wright et al., 2006) | 24 | 11.2% |
|  | Unclear/Other measures | 14 | 6.6% |
|  | Loneliness (De Jong e al., 1985, 2006; DiTommaso & Spinner, 1993) | 16 | 7.5% |
|  | Theoretical/Review | 4 | 1.9% |
|  | Meta-analyses | 3 | 1.4% |
| Publications | Average Journal Impact Factor (IF)= 4.2 | | **Percentage (out of 213)** |
|  | No impact factor | 15 | 7.0% |
|  | IF < 2 | 39 | 18.3% |
|  | IF ≥ 2 and <4 | 77 | 36.6% |
|  | IF ≥ 4 and <6 | 44 | 20.7% |
|  | ≥ 6 | 38 | 17.4% |

***Note**. There are a total of 213 published papers, some of which have multiple studies. In total, 236 studies were reviewed, 233 of which were empirical studies (13 papers had multiple studies; 3 were conceptual/theoretical with no data).

|  | **Study** | **Year** | **Sample** | **Country** | **Study Design Characteristics** | **Trait or State** | **Working adults (Y/N)** | **Measure of Loneliness** | **Antecedent/ Outcome/ Moderator / Intervention** | **Primary Findings** | **Journal Name** | **Journal Impact Factor (2022)** |
| --- | --- | --- | --- | --- | --- | --- | --- | --- | --- | --- | --- | --- |
| 1 | Aase, Nordrehaug, & Malterud (2008) | 2008 | Interviews with 10 physicians | Norway | Qualitative Single source | Trait-like | Yes | Qualitative | Antecedents | - Belonging to a community of fellows helped with loneliness. - Some contended that loneliness was not an issue, as they never worked alone. - Others noted the medical culture of competition, pride, and prestige, where people are expected to be tough and perfect as a source of loneliness. - They also described loneliness when facing tough situations at night, in decision-making, or not being   listened to in a team. | Journal of Medical Ethics | 4.2 |
| 2 | Abelsen, Simen, Nordbo (2023) | 2023 | 357 individuals working from home during Covid-19 | India Norway US | Cross sectional Single source | Trait-like | Yes | The social loneliness subscale from DiTommaso & Spinner (1993) | Antecedents Outcomes | - Loneliness was significantly correlated with self-rated work performance - When the technology used was a good fit for work   meetings, loneliness was reduced. | Information Technology & People | 5 |
| 3 | Abrams, Finlay, & Kobayashi (2022) | 2022 | 6264 individuals from the US Covid-19 coping study, people over 55 | US | Cross sectional Single source | Trait-like | No | 3 item short form of R-UCLA loneliness scale | Antecedents | - During Covid-19, compared to people whose work has not changed, those who lost their jobs or those who were furloughed were significantly more lonely. - No effects for those not working, or reduced hours, income, or working from home. | Journals of Gerontology Series B: Psychological Sciences & Social Sciences. | 6.2 |
| 4 | Ahmed & Al-Dhuwaihi (2020) | 2020 | 12 newly hired high school principals | Saudi Arabia | Qualitative Single source | Trait-like | Yes | Qualitative | Antecedents | - Being a new leader was associated with feelings of loneliness. | School Leadership & Management | 5.2 |
| 5 | Allen & Oshagan (1995) | 1995 | 619 adults | US | Cross sectional Single source | Trait-like, though they write about how the items are "consistent with recent work on affective mood states" such as Watson and Clark's PANA, so could be either trait-like or state-like | No | 7 item short form of R-UCLA loneliness scale | Antecedents | - No differences were found in loneliness based on employment status. - Those with lower income exhibited higher loneliness. | Personality & Individual Differences | 4.3 |
| 6 | Alroomi & Mohamed (2022a) | 2022 | 387 immigrant employees working in Kuwaiti oil fields | Kuwait | Cross sectional Single source | Trait-like | Yes | 4 item short form of R-UCLA loneliness scale | Outcomes | - Loneliness was associated with physical and mental fatigue. - Mental fatigue mediated the relation between   loneliness and safety compliance. | Safety Science | 6.1 |
| 7 | Alroomi & Mohamed (2022b) | 2022 | 387 oil workers in Kuwait | Kuwait | Cross sectional Single source | Trait-like | Yes | 4 item short form of R-UCLA loneliness scale | Outcomes | - Loneliness was negatively related to safety participation and safety compliance - Anxiety mediated loneliness - safety participation and safety compliance relationship, but depression did   not. | Journal of Loss Prevention in the Process Industries | 3.5 |
| 8 | Amarat, Akbolat, Ünal, & Güneş Karakaya (2019) | 2019 | 138 nurses in Turkey | Turkey | Cross-sectional Single source | Trait-like | Yes | The Wright et al. (2006) loneliness at work scale | Outcomes | - Workplace loneliness predicted self-reported job performance, mediated by work alienation. | Journal of Nursing Management | 5.5 |
| 9 | Anand & Mishra (2021) | 2021 | Sample 1: 340 nurses in India  Sample 2: 907 call center employees in India | India | Cross sectional Single source | Trait-like | Yes | 20 item R-UCLA loneliness scale adapted to work | Antecedent Outcome | - Core self-evaluations were negatively related to loneliness. - Loneliness was positively related to emotional exhaustion. - Loneliness was more strongly and positively related   to emotional exhaustion when LMX was high. | International Journal of Human Resource Management | 5.6 |
| 10 | Andel, Shen, & Arvan (2021) | 2021 | Eight weekly surveys on 265 US workers (Prolific) | US | Time lagged (8 waves)  Single source | State-like | Yes | 5 items from the UCLA scale adapted to work | Antecedents Outcomes Moderator | - Weekly telecommuting frequency, job insecurity, and lack of Covid-19-related informational justice were related to greater work loneliness. - Weekly work loneliness was related to more depressive symptoms and fewer OCBs during that week. - Self compassion buffered the loneliness-outcome   relationship | Journal of Occupational Health Psychology | 5.1 |
| 11 | Anzola, Limoges, McLean, & Kolla (2022) | 2022 | 240 employees from a psychiatric hospital and an acute care hospital in Canada | Canada | Cross sectional Single source | Trait-like | Yes | 20 item R-UCLA loneliness scale | Antecedents Outcomes | - Loneliness was positively correlated with depression, anxiety, and stress. It was negatively correlated with work-related quality of life and resilience. | Frontiers in Psychiatry | 4.7 |

|  | **Study** | **Year** | **Sample** | **Country** | **Study Design Characteristics** | **Trait or State** | **Working adults (Y/N)** | **Measure of Loneliness** | **Antecedent/ Outcome/ Moderator / Intervention** | **Primary Findings** | **Journal Name** | **Journal Impact Factor (2022)** |
| --- | --- | --- | --- | --- | --- | --- | --- | --- | --- | --- | --- | --- |
| 12 | Arslan, Yener, & Schermer (2020) | 2020 | 864 nurses in Turkey | Turkey | Cross sectional Single source | Trait-like | Yes | The loneliness at work scale (Wright et al., 2006) | Antecedents Moderator | - LMX social exchange negatively predicted loneliness. - Trust and meaningfulness were mediators of this relationship. - Communication with the leader positively moderated LMX social exchange – trust and meaningfulness   relationship. | Journal of Nursing Management | 5.5 |
| 13 | Ayalon & Shiovitz-Ezra (2010) | 2010 | 29 Filipino homecare workers, 7 care recipients, 23 family members of care recipients, and 31 social workers in Israel | Israel | Qualitative Multi-source | Trait-like | Yes | Qualitative | Antecedents Outcomes | - Filipino home care workers reported emotional and social loneliness. Having to leave their spouses and kids behind contributed to this. - Reunifications that are of short duration do not help because of their temporary nature. - They do not receive support from clients, because they are perceived as emotionless for having left kids behind. ho - Not knowing the language contributes to social loneliness. - Loneliness affects worker health and the quality of   care provided. | British Journal of Social Work | 1.8 |
| 14 | Bareket-Bojmel, Chernyak-Hai, & Margalit (2023) | 2023 | 349 employees from the US and UK | US and UK | Cross sectional Single source | Trait-like | Yes | 3 item short form of R-UCLA loneliness scale | Loneliness as moderator | - Loneliness moderated the relation between remote work and job engagement. When loneliness was high, remote work was negatively related to engagement. For low loneliness, no relationship was found. - Loneliness also moderated the relation between remote work and hope. There was a positive relation   only when loneliness was low. | Personality & Individual Differences | 4.3 |
| 15 | Basit & Nauman (2023) | 2023 | 274 employees in Pakistan | Pakistan | Cross sectional Single Source | Trait-like | Yes | 20-item R-UCLA loneliness scale adapted to work | Outcomes | - Workplace loneliness reduced work engagement, and indirectly affected job dissatisfaction. - Need to belong moderated this relationship such that for those with low need to belong, loneliness - work engagement relationship was stronger and more   negative. | Frontiers in Psychology | 3.8 |
| 16 | Bayar (2020) | 2020 | 12 school administrators in Turkey | Turkey | Qualitative Single source | Trait-like | Yes | Qualitative | Antecedents | - Perception of loneliness school administrators reported was due to lack of regular communication, lack of support from environment and superiors, struggles with teachers, legal issues and pressures from the top, economic troubles, other administrators, technology addiction. | Educational Research Quarterly | NONE |
| 17 | Bayraktar & Jiménez (2022) | 2022 | 474 small business owners in Turkey | Turkey | Cross sectional Single source | Trait-like | Yes | The de Jong Gierveld & van Tilburg (2006) loneliness scale | Antecedents | - Obsessive passion (an uncomfortable urge to do something) was positively related to loneliness. | Cross Cultural & Strategic Management | 2.5 |
| 18 | Bazemore, Janda, Derlega, & Paulson (2010) | 2010 | 162 foreign-born college professors in the USA | foreign-born, but in US | Cross sectional Single source | Trait-like | Yes | The de Jong Gierveld & van Tilburg (2006) loneliness scale | Antecedents | - Stigma and self-esteem did not directly relate to loneliness. - Higher levels of stigma consciousness were indirectly related to loneliness via rejection. - Rejection also mediated the self-esteem and   loneliness relation. | Journal of Diversity in Higher Education | 2.4 |
| 19 | Becker, Belkin, Tuskey, & Conroy (2022) | 2022 | 239 working adults recruited during early Covid and then 2 weeks later in the USA (all remote workers) | US | Time lagged Single source | Trait-like | Yes | 5 items from the R-UCLA scale adapted to work | Outcomes | - Work related loneliness did not interact with segmentation preferences. - Work loneliness predicted emotional exhaustion and work life balance (-). - Work loneliness had indirect effects on depression   via exhaustion, but not on CWB or insomnia. | Human Resource Management | 6.6 |
| 20 | Bennett, Manchaiah, Eikelboom, Badcock, & Swanapoel (2022) | 2022 | 239 audiologists from around the world | Global | Cross sectional Single source | Trait-like | Yes | 3 item short form of R-UCLA loneliness scale | Antecedents | - Perceived job insecurity was related to loneliness. - Availability of workplace psychological support was negatively related to loneliness | International Journal of Audiology | 2.7 |
| 21 | Bessaha et al. (2020) | 2020 | Review of intervention studies | NA | Review | Trait-like | No | Review | Antecedents Intervention | - Several interventions, particularly those involving technology and support groups, reduced loneliness. | Clinical Social Work Journal | 2.8 |

|  | **Study** | **Year** | **Sample** | **Country** | **Study Design Characteristics** | **Trait or State** | **Working adults (Y/N)** | **Measure of Loneliness** | **Antecedent/ Outcome/ Moderator / Intervention** | **Primary Findings** | **Journal Name** | **Journal Impact Factor (2022)** |
| --- | --- | --- | --- | --- | --- | --- | --- | --- | --- | --- | --- | --- |
| 22 | Bismark et al. (2022) | 2022 | Free text responses from 7795 healthcare workers in Australia | Australia | Qualitative Single source | Trait-like | Yes | Qualitative study | Antecedent | - Covid-19 contributed to loneliness among nurses. 25% of nurses who had frequent thoughts of suicide and self-harm felt lonely. Loss of camaraderie at work, increased bullying and exclusion, and being ostracized by the public were among the reasons. - Workplace support felt disingenuous and inadequate. - Knowing that others were also struggling and having someone nonjudgmental to talk to made them feel less alone. - Living alone, being separated from loved ones, isolating oneself to keep others safe, and feeling that no one cared or helped them were other risk factors for   loneliness. | BJPsych Open | 5.4 |
| 23 | Blomqvist, Virtanen, Westerlund, & Magnusson Hanson (2023) | 2023 | 1231 participants from Swedish Longitudinal Occupational Survey during the pandemic | Sweden | Cross Sectional Single source | Trait-like | No | 3 item UCLA scale before and during the pandemic | Antecedent Outcome | - Job insecurity predicted increases in loneliness. - Loneliness affected anxiety symptoms and depression | Scandinavian Journal of Public Health | 3.4 |
| 24 | Bollestad, Amland, & Olsen (2022) | 2022 | 1511 Norwegian workers during Covid restrictions | Norway | Cross sectional Single source | Trait-like | Yes | 2 items (lack of contact with other people, feelings of isolation) | Antecedents | - Remote work had positive direct effects on loneliness. - Remote work had indirect negative effects on loneliness via reduced bullying. | Frontiers in Psychology | 3.8 |
| 25 | Bonsaksen et al. (2023) | 2023 | 1649 adults in Norway, USA, UK and Australia | Norway, US, UK, and Australia | Cross sectional Single source | Trait-like | No | The six-item de Jong Gierveld & van Tilburg (2006) loneliness scale | Antecedent | - Employment was negatively related to loneliness. | Health Psychology and Behavioral Medicine | 2.7 |
| 26 | Bonsaksen, Ruffolo, Leung, Price, Thygesen, Schoultz, & Geirdal (2021) | 2021 | 3810 participants from UK, Norway, US, and Australia during Covid-19 | Norway, US, UK, and Australia | Cross sectional Single source | Trait-like | No | The six-item de Jong Gierveld & van Tilburg (2006) loneliness scale | Antecedents | - Having employment negatively predicted social and emotional loneliness across all age groups. | Social media + society | 5.2 |
| 27 | Bornstein & Magnus (2022) | 2022 | 577 participants in the US during Covid closures | US | Cross sectional Single source | Trait-like | No | Single Item: “Over the past month, which of the following has applied to you?”. Participants had the option to select the response, “I feel lonelier than usual”. | Antecedent | - Being employed part time was associated with feelings of higher loneliness, whereas full time employment was associated with similar or lower loneliness. | Psychiatry Research | 11.3 |
| 28 | Bovornusvakool, Vodanovich, Ariyabuddhiphongs, & Ngamake (2012) | 2012 | 325 undergraduate students in the USA | US | Cross sectional Single source | Trait-like | No | 20-item R-UCLA loneliness scale | Antecedents | - Perfectionism was related to workaholism, which predicted leisure boredom and loneliness. - Perfectionism and negative affect also had direct   effects on loneliness. | Psychologist-Man ager Journal | 0.6 |
| 29 | Bower et al. (2023) | 2023 | 1523 Australian adults  surveyed 3 times over 18 months during Covid | Australia | Time lagged (3 waves)  Single source | Trait-like | No | 6 item de Jong Gierveld & van Tilburg (2006) loneliness scale | Antecedent | - Changes in loneliness mirrored Covid-19 related closures - Men with mental health disorders expressed more loneliness. - Higher income was related to lower levels of loneliness. - LGBTQ status, age, income loss, current employment, and education were not related to loneliness. - Resilience and perceived control were important   predictors of loneliness. | Behaviour Change | 1.1 |
| 30 | Bryan, Andrews, Thompson, Qualter, Matthews & Arsenault (2023) | 2023 | Meta analysis of loneliness at work | Global | Trait-like State-like | Trait-like State-like | Yes | Meta-analysis | Outcomes | - Workplace loneliness was associated with lower job performance, job satisfaction, and worker-manager relations. - Workplace loneliness was associated with higher   levels of burnout. | Occupational Medicine | 2.8 |

|  | **Study** | **Year** | **Sample** | **Country** | **Study Design Characteristics** | **Trait or State** | **Working adults (Y/N)** | **Measure of Loneliness** | **Antecedent/ Outcome/ Moderator / Intervention** | **Primary Findings** | **Journal Name** | **Journal Impact Factor (2022)** |
| --- | --- | --- | --- | --- | --- | --- | --- | --- | --- | --- | --- | --- |
| 31 | Buecker, Denissen, & Luhmann (2021) | 2021 | Nationally representative sample of 13,945 individuals in the Netherlands | the Netherlands | Time lagged (18 waves) Archival Data  Single source | Trait-like | No | 6 item de Jong Gierveld & van Tilburg (2006) loneliness scale | Antecedents Trajectories | - Transitioning to paid employment did not change the trajectory of loneliness compared to the control group. - Starting employment after previous unemployment did not change the trajectory of loneliness, but for adults 10 years older than average there was a greater reduction in loneliness. - Job loss was related to a long-lasting baseline change in loneliness. - Retirement did not lead to changes in loneliness but those who were younger than average experienced   increased loneliness. | Journal of Personality and Social Psychology | 7.6 |
| 32 | Cabello, Izquierdo, & Leal (2022) | 2022 | 1421 health care professionals in Spain | Spain | Cross sectional Single source | Trait-like | yes | 3 item short form of R-UCLA loneliness scale | Outcomes | - Loneliness was related to higher mental health problems. | Health & Social Care in the community | 2.4 |
| 33 | Cacioppo et al. (2016) | 2016 | 662 soldiers | US | Cross sectional Single source | Trait-like | Yes | 9 item R-UCLA scale | Antecedents | - Age, childhood trauma, intra-platoon harassment, perceived stress, and OCB were positively related to loneliness. - Frequent phone contact, emotional health, platoon cohesion, relationship satisfaction with friends and   platoon members were negatively related to loneliness. | Journal of Social and Clinical Psychology | 1.7 |
| 34 | Cacioppo, Adler, Lester, McGurk, Thomas, Chen, & Cacioppo (2015) | 2015 | 346 soldiers who participated in a social resilience training, and 235 who participated in cultural awareness training | USA | pre-post with control Intervention Single source | State-like | Yes | 9 item R-UCLA, referencing last two weeks | Intervention | - Compared to social awareness training, social resilience training resulted in small, but greater reductions in loneliness. | Journal of Personality and Social Psychology | 7.6 |
| 35 | Cardon & Arwine (2023) | 2023 | Qualitative analysis of over 9000 reddit posts | NA | Qualitative Single source | Trait-like | Yes | Qualitative | Antecedents Outcomes | - Some entrepreneurs view loneliness as threatening and harmful whereas others see it as positive and irrelevant, which drives need to cope. - Antecedents include nature of work (high burden of responsibility, lack of coworker support, social maladaptation), lack of resources (time, have to grind), and isolation (social, cognitive, and physical). - Authors identified entrepreneurs who viewed loneliness as a positive aspect of their work, allowing them to focus on their business goals and develop resilience. The temporary isolation allows for introspection and strategic thinking, potentially leading to long-term benefits. | Personnel Psychology | 5.5 |
| 36 | Carlson, Rämgård, Bolmsjö, & Bengtsson (2014) | 2014 | 30 nurses engaged in  home-based or long-term care in nursing homes | Sweden | Qualitative Single source | Trait-like | Yes | Qualitative | Antecedents | - Nurses reported having to balance autonomy and a sense of loneliness at work. | International Journal of Nursing Studies | 8.1 |
| 37 | Carr, Lennox Kail, Matz-Costa, & Shavit (2018) | 2018 | 5882 married adults 51+ | US | Time lagged (2 waves)  Single source | Trait-like | No | 3 item short form of R-UCLA loneliness scale | Antecedents Moderator | - For those who become widowed, loneliness is significantly higher than those who stay continuously married. However, starting to volunteer 2+ hours per week attenuated this relationship. | Journals of Gerontology Series B: Psychological Sciences & Social Sciences. | 6.2 |
| 38 | Cenkci & Bircan (2023) | 2023 | 464 adult employees working in higher education during Covid-19 Pandemic | US | Cross sectional Single source | Trait-like | Yes | The Wright et al. (2006)  loneliness at work  scale | Antecedents | - Interactional justice fully mediated the relation between authentic leadership and workplace loneliness. | Management Research Review | 2.6 |

|  | **Study** | **Year** | **Sample** | **Country** | **Study Design Characteristics** | **Trait or State** | **Working adults (Y/N)** | **Measure of Loneliness** | **Antecedent/ Outcome/ Moderator / Intervention** | **Primary Findings** | **Journal Name** | **Journal Impact Factor (2022)** |
| --- | --- | --- | --- | --- | --- | --- | --- | --- | --- | --- | --- | --- |
| 39 | Chan & Qiu (2011) | 2011 | 213 migrant workers in China with at least one-year work history | China | Cross sectional Single source | Trait-like | Yes | 20 item R-UCLA loneliness scale | Antecedents Outcomes | - Loneliness did not vary by age or gender. - Single workers felt lonelier than married ones. - Lonelier workers unexpectedly reported higher job satisfaction. There was no relation to organizational commitment. - Authors found that lonelier migrant workers reported higher job satisfaction, suggesting that loneliness might drive individuals to focus more on their work to find fulfilment and purpose. This finding, while seemingly counterintuitive, can be interpreted through the evolutionary lens as an adaptive response to social isolation. By focusing on their work and achieving success, individuals may enhance their social standing, ultimately leading to increased social connection and a   reduction in loneliness. | International Journal of Human Resource Management | 5.6 |
| 40 | Chen, Cao, Qu, Kang, Xiang, & Tang (2023) | 2023 | 267 employee-peer dyads in China | China | Time lagged (3-waves)  Multi source (employees, peers) | Trait-like | Yes | 20 item R-UCLA loneliness scale adapted to work | Antecedents | - There is an inverted U-shaped relationship between promotion-oriented relational job crafting and loneliness via peer social undermining. - Prevention-oriented relational job crafting is positively related to loneliness through peer social   undermining. | Applied Psychology: An International Review | 7.2 |
| 41 | Chen, Peng, Lei, & Zou (2021) | 2021 | 133 teams from 6 companies  in China. (133 leaders, 561 members) | China | Time lagged Multi-source | Trait-like | Yes | The Wright et al (2006) workplace loneliness scale | Outcomes Moderators | - Leader workplace loneliness was positively related to team turnover intentions via the effects of team cognitive trust in leader (and not affective trust in leader) - Team workplace loneliness strengthened the negative relation between leader workplace loneliness and team cognitive trust in leader, as well as the indirect effects   on team turnover intentions. | Asian Business & Management | 3.9 |
| 42 | Chen, Wen, Peng, & Liu (2016) | 2016 | 232 dyads from 10 companies in China | China | Cross sectional Multi-source | Trait-like | Yes | The Wright et al. (2006) workplace loneliness scale | Outcomes | - Leader-member congruence in loneliness predicted LMX. - LMX is higher when the leader and member are aligned at low LMX. - When they are incongruent, leader loneliness being higher than the employee was worse for LMX. - LMX mediated leader-member loneliness   congruence and turnover intentions relation. | Journal of Managerial Psychology | 3.2 |
| 43 | Cheng, Sun, Zhong, & Li (2023) | 2023 | S1: 314 employees in China from various industries  S2: 343 employees from a large group company in China over 3 waves | China | S1: Cross-sectional, single source  S2: Time lagged (3 waves);  Single-source | Trait-like | Yes | Studies 1-2: The Wright et al. (2006) workplace loneliness scale | Antecedent Outcome Moderator | - Flexible work arrangements predicted higher loneliness, which reduced knowledge sharing. - High task interdependence weakened the relationship between flexible work arrangements and workplace loneliness, with the relation positive only for low task interdependence. - High task interdependence also weakened the relationship between workplace loneliness and self rated knowledge sharing, with stronger effects under low task   interdependence. | Behavioral Sciences | 2.6 |
| 44 | Chou & Chang (2016) | 2016 | Theoretical | NA | Theoretical | Trait-like | - | Theoretical | Antecedents | - Predicted that interpersonal helping behavior rejections received from existing team members will have a positive effect on newcomers’ workplace loneliness - This effect will be mediated by TMX and newcomer’s team identification - Task interdependence will moderate the relation between interpersonal helping behavior rejection and newcomer workplace loneliness such that the relation   will be stronger when task interdependence is high. | Team Performance Management | 1.7 |

|  | **Study** | **Year** | **Sample** | **Country** | **Study Design Characteristics** | **Trait or State** | **Working adults (Y/N)** | **Measure of Loneliness** | **Antecedent/ Outcome/ Moderator / Intervention** | **Primary Findings** | **Journal Name** | **Journal Impact Factor (2022)** |
| --- | --- | --- | --- | --- | --- | --- | --- | --- | --- | --- | --- | --- |
| 45 | Cooper & Quick (2003) | 2003 | Theoretical | NA | Theoretical | Trait-like | - | Theoretical | Antecedents Outcomes | - Leaders may feel lonely and feel disconnected from the rest of the organization. - When loneliness goes unchecked, it could lead to depression. - Loneliness may lead to being left without accurate information about the organization, further leading to loneliness. - To cope with loneliness, people need real human connection and contact with another person. Simply being with others is not enough. - Preventive measures may include executive coaching, external peer support, confidants, and   journaling. | Counselling Psychology Quarterly | 3 |
| 46 | Creed & Reynolds (2001) | 2001 | 148 youth | Australia | Cross sectional Single source | Trait-like | No | The social loneliness subscale from DiTommaso & Spinner (1993) | Antecedents | - Those who were unemployed with no paid work felt lonelier than those unemployed with regular paid work. Experiential deprivation was positively related to loneliness but economic deprivation was not. | Journal of Community & Applied Social Psychology | 2.7 |
| 47 | Cubitt & Burt (2002) | 2002 | 293 New Zealand primary school principals | New Zealand | Cross sectional Single source | Trait-like | Yes | 20 item R-UCLA loneliness scale | Antecedents Outcomes | - Loneliness was significantly correlated with stress. - Principals who were teaching reported higher loneliness compared to principals who were only leading. - There was no relation between leadership style and   loneliness. | New Zealand Journal of Educational Studies | 0.9 |
| 48 | Cui et al. (2020) | 2020 | 12 nurses who voluntarily supported Covid patients in China | China | Qualitative Single source | Trait-like | Yes | Qualitative | Antecedents | - Covid-related isolation contributed to loneliness, but support from coworkers helped alleviate it. | Psychology Research and Behavior Management | 4.3 |
| 49 | D'Oliveira & Persico (2023) | 2023 | 137 working adults | UK | Cross sectional Single source | Trait-like | Yes | Wright et al. (2006) loneliness in the workplace subscale | Outcome | - Loneliness had indirect effects on workplace wellbeing mediated by supportive behaviors. - Loneliness was also negatively related to task   interdependence. | Applied Ergonomics | 3.2 |
| 50 | Daniel, Di Domenico, & Nunan (2018) | 2018 | 23 self-employed knowledge workers | UK | Qualitative Single source | Trait-like | Yes | Qualitative | Antecedents | - Most interviewees mentioned loneliness in relation to working from home. - They invented reasons to leave the house to cope with loneliness - Balancing loneliness with solitude necessary for   creativity was a theme. | Journal of Management Studies | 10.5 |
| 51 | De Visser, de Graaf-Zijl, Anema, & Huysmans (2023) | 2023 | 10 interviews in Netherlands | the Netherlands | Qualitative Single source | Trait-like | No | Qualitative | Antecedent | - During Covid-19, job searchers were especially prone to loneliness. | BMC Public Health | 4.5 |
| 52 | Deutrom, Katos, & Ali (2022) | 2022 | 299 remote workers in the UK (prolific) | UK | Cross sectional Single source | Trait-like | Yes | 3 item short form of R-UCLA loneliness scale | Outcomes | - Loneliness predicted problematic internet use (obsessive thinking about the internet, withdrawal symptoms when not using the internet), which in turn negatively affected cyber security behaviors (e.g., creating strong passwords, securing devices). | Behaviour & Information Technology | 3.7 |
| 53 | Di Napoli et al. (2021) | 2021 | 12408 employed immigrants in Italy | Italy | Cross sectional Single source | Trait-like | Yes | Single item: “Do you feel lonely in Italy?” | Antecedents Outcomes | - Self-perceived discrimination was related to higher loneliness. - Loneliness was negatively related to mental and   physical health. | BMC Psychiatry | 4.4 |
| 54 | Ding, Li, Li, Li, Xie, & Duan (2023) | 2023 | 1774 nurses in China | China | Cross sectional Single source | Trait-like | Yes | 3 item short form of R-UCLA loneliness scale | Antecedents | - Workplace violence predicted loneliness | BMC Nursing | 3.2 |
| 55 | Dor-Haim (2023) | 2023 | 19 interviews with Israeli deputy principals | Israel | Qualitative Single source | State-like | Yes | Qualitative | Antecedents | - Loneliness was perceived as separateness, as abandonment, as carrying the burden of responsibility, and aloneness in communication to the school. | Educational Management Administration and Leadership | 3.6 |
| 56 | Dor-Haim & Oplatka (2020) | 2020 | 8 school principals in Israel | Israel | Qualitative Single source | State-like | Yes | Qualitative | Antecedents | - Loneliness was associated with two factors among school principals: 1) Being disappointed in school staff and seeing them as not committed to work. 2) overidentification with the school and the criticism of the school feels personal. | Journal of Educational Administration & History | 1.8 |

|  | **Study** | **Year** | **Sample** | **Country** | **Study Design Characteristics** | **Trait or State** | **Working adults (Y/N)** | **Measure of Loneliness** | **Antecedent/ Outcome/ Moderator / Intervention** | **Primary Findings** | **Journal Name** | **Journal Impact Factor (2022)** |
| --- | --- | --- | --- | --- | --- | --- | --- | --- | --- | --- | --- | --- |
| 57 | Dor-Haim & Oplatka (2021) | 2021 | 12 school principals in Israel | Israel | Qualitative Single source | Trait-like | Yes | Qualitative | Antecedents Moderators | - Principles reported feeling lonely at work because of a sense fo abandonment by superiors, and a sense of alienation from staff. - The strategies they report using include looking for support outside the school, an attempt to promote better   relations with staff, and initiating change in the school. | Leadership and Policy in Schools | 1.1 |
| 58 | Du, Ma, & Lee (2022) | 2022 | 639 employees from various industries in China during Covid-19 | China | Cross sectional Single source | Trait-like | Yes | Unclear (workplace loneliness) | Antecedents Outcomes Moderator | - Collaborative intra-team climate was negatively related to workplace loneliness. - Competitive intra-team climate was positively related to workplace loneliness. - These effects were moderated by need to belong such that, each relationship was weaker when need to belong was high. - Loneliness was correlated with knowledge hoarding. | Sustainability | 3.9 |
| 59 | Elbogen et al. (2022) | 2022 | 902 middle and low income adults via MTurk. Min 22 years old and experiencing shift to Zoom. | US | Cross sectional Single source | Trait-like | Yes | 4 item short form of R-UCLA loneliness scale | Antecedent Outcomes | - Loneliness was correlated with zoom fatigue and depressive symptoms. - Out of all the variables, only loneliness, being married, and number of zoom calls per week predicted   zoom fatigue. | Cyberpsychology, Behavior, and Social Networking | 6.6 |
| 60 | Fardghassemi & Joffe (2022) | 2022 | 48 working, lower SES young adults in London England | UK | Qualitative Single source | Trait-like | Yes | Qualitative | Antecedents | - Antecedents of loneliness included the feeling of being disconnected, contemporary culture, pressure, social comparison, and transition between life stages. | Plos One | 3.7 |
| 61 | Febriani, Hasanah, Roz, & Hakim (2023) | 2023 | 300 female employees | Indonesia | Cross sectional Single source | Trait-like | Yes | Unclear (workplace loneliness) | Antecedents Outcomes | - Workplace spirituality was negatively associated with loneliness. - Work-Family conflict was positively related to loneliness. - Loneliness was negatively related to intention to stay   with the company. | International Journal of Professional Business Review | NONE |
| 62 | Firoz & Chaudhary (2022) | 2022 | Sample 1: 375 employees in manufacturing in India  Sample 2: 559 service professionals in India | India | Time lagged (1 month)  Single source | Trait-like | Yes | The Wright et al. (2006) workplace loneliness scale | Outcomes Moderator | - Workplace loneliness was negatively related to creative performance and OCB (self rated, 1 month apart) - Workplace loneliness was positively related to work-family conflict - Psychological Capital weakened the relation between WL and creative performance, as well as OCB - The moderating effect of PsycCap on WL-work family conflict relation was different: The relationship was weaker for those who were lower in PsycCap. This finding suggests that loneliness neutralizes the   beneficial effects of PsyCap on work-life conflict. | Personnel Review | 3.9 |
| 63 | Fry & Bloyce (2017a) | 2017 | 20 professional migrant golfers | Global | Qualitative Single source | Trait-like | Yes | Qualitative | Antecedents | - Friendship networks are useful to golfers to deal with loneliness. (These are friendships of convenience). - Particularly stress and poor play contribute to a sense of loneliness. - Because success and failure are completely individual, it is a lonely game. - The authors discuss how golfers form friendships and support networks to combat loneliness and isolation, which could suggest that loneliness can act as a catalyst for social bonding. This can be inferred from statements on pages 151, 152, and 153 where the authors highlight the importance of social support and companionship   among golfers. | Sociology of Sport Journal | 1.7 |
| 64 | Fry & Bloyce (2017b) | 2017 | 20 professional migrant golfers | Global | Qualitative Single source | Trait-like | Yes | Qualitative | Antecedents | - Professional golfers lead nomadic lives and spend a lot of time away from home, contributing to loneliness and a sense of being cut off from the world. They do not have family or friends with them and limited opportunities to maintain contact. - To cope, they attempted to develop friendship groups on tour. | International Review for the Sociology of Sport | 2.3 |
| 65 | Fujii, Konno, Tateishi, Hino, Tsuji, Ikegami, Nagata, Yoshimura, et al. (2021) | 2021 | 27,036 Japanese workers during the Covid-19 pandemic | Japan | Cross sectional Single source | Trait-like | Yes | Single item (during the last 30 days, how often have you felt lonely) | Antecedents | - Time spent with family decreases loneliness. - Loneliness increased for those living with someone in need of care, and declined for those living with a spouse. | Frontiers in Psychiatry | 4.7 |

|  | **Study** | **Year** | **Sample** | **Country** | **Study Design Characteristics** | **Trait or State** | **Working adults (Y/N)** | **Measure of Loneliness** | **Antecedent/ Outcome/ Moderator / Intervention** | **Primary Findings** | **Journal Name** | **Journal Impact Factor (2022)** |
| --- | --- | --- | --- | --- | --- | --- | --- | --- | --- | --- | --- | --- |
| 66 | Gabriel, Lanaj, & Jennings (2021) | 2021 | Study 1: 86 leaders in a 10-day experience sampling study  Study 2: 62 leader member dyads | US | S1: Experience sampling Single source  S2: Experience sampling Multi-source | State-like | Yes | Studies 1-2: 3 item short form of  R-UCLA loneliness scale, adapted to loneliness from subordinates (e.g., I lacked companionship from one or more of my subordinates). | Outcomes | - Daily leader loneliness predicted affect focused rumination and problem-solving pondering, - Leader loneliness resulted in affect-based rumination, which reduced helping. At the same time, leader loneliness predicted problem-solving pondering, facilitating engagement, and helping. - Leader self-efficacy strengthened leader loneliness-problem solving pondering relationship. - In Study 2, on days leaders experienced loneliness from their followers, their followers reported lower leadership effectiveness and empowering leadership. - Authors found that daily leader loneliness can trigger problem-solving pondering, a form of rumination focused on finding solutions to work-related challenges. This adaptive response to loneliness can enhance work engagement and helping behaviors, ultimately reducing future feelings of loneliness and contributing to   leadership effectiveness. | Journal of Applied Psychology | 9.9 |
| 67 | George-Levi, Schmidt-Barad, Natan, & Margalit (2022) | 2022 | 104 Israeli school psychologists | Israel | Cross sectional Single source | Trait-like | Yes | 11 items, The de Jong Gierveld & van Tilburg (2006) loneliness scale | Moderator | - Loneliness moderated the negative relation between sense of coherence and burnout, with the strongest effects when loneliness was low. | Current Psychology | 2.8 |
| 68 | Geragty, Oliver, & Wang (2022) | 2022 | 91 midwifery and nursing academics in the UK | UK | Qualitative Single source | Trait-like | Yes | Qualitative | Antecedents | - Working from home during the pandemic was associated with loneliness. - Some participants felt isolated, but not lonely (introverts, those who had spouses and kids at home) - Those who felt lonely mentioned missing collegiality   of colleagues and connectedness, face to face teaching. | British Journal of Midwifery | NONE |
| 69 | Gevaert, Bosmans, De Moortel, & Vanroelen (2023) | 2023 | 19 Self Employed workers in Belgium | Belgium | Qualitative Single source | Trait-like | Yes | Qualitative | Antecedents | - Self employment and the associated independence was related to loneliness. | Businesses | NONE |
| 70 | Gilmer, Magley, Dugan, Namazi, & Cherniack (2023) | 2023 | Sample 1 = 681 working adults in China, via Qualtrics  Sample 2 = 165 state corrections supervisors | China | Study 1 and 2: Cross sectional  Single source | Trait-like | Yes | Sample 1 = 20 item R-UCLA adapted to work  Sample 2 = general loneliness, R-UCLA 3 items | Outcomes | - Both workplace loneliness and general loneliness explained substantial variance in outcomes such as emotional exhaustion, depression symptoms, and turnover intentions when controlling for variance explained by workplace civility. - When controlling for work stress, general loneliness is more important than incivility in explaining variance in emotional exhaustion, job satisfaction, and depression symptoms. | Occupational Health Science | 3.1 |
| 71 | Gjerstad et al. (2020) | 2020 | 1627 Norwegian individuals who were deployed 30 years ago | Norway | Cross sectional Single source | Trait-like | No | Single item: “I felt lonely” | Outcomes | - Loneliness during deployment (30 years ago) predicted PTSD symptoms experienced during the pandemic. | Frontiers in Psychology | 3.8 |
| 72 | Ghosh (2023) | 2023 | 537 IT employees in a large multinational firm in India | India | Qualitative Multi source | Trait-like | Yes | The UCLA  loneliness scale (Russell, 1996) | Outcomes | - Significant negative relationship between loneliness and work performance as rated by supervisors. | European Management Journal | 7.5 |
| 73 | Gong (2023) | 2023 | 583 employees in China | China | Qualitative Single source | Trait-like | Yes | 3 item short form of R-UCLA loneliness scale | Antecedents Outcomes | - Loneliness was negatively correlated with age, employee wages, and life satisfaction. - Loneliness was positively correlated with depression. | European Review of Applied Psychology | 1.3 |
| 74 | Griffiths, Gray, Kyle, Song, & Davies (2022). | 2022 | 615 working adults in Wales, UK (population survey) | UK | Cross sectional Single source | Trait-like | Yes | Those  stating that they were able to work from home (N = 299) were asked how working from home had affected 7 aspects of their health and  well-being (“feelings of loneliness,”) | Antecedents | - 45% of those working from home reported that they felt lonelier compared to prior to the pandemic. - Those in their 30s were three times more likely to report increased loneliness compared to those in 40s. | Journal of Occupational & Environmental Medicine | 3.2 |

|  | **Study** | **Year** | **Sample** | **Country** | **Study Design Characteristics** | **Trait or State** | **Working adults (Y/N)** | **Measure of Loneliness** | **Antecedent/ Outcome/ Moderator / Intervention** | **Primary Findings** | **Journal Name** | **Journal Impact Factor (2022)** |
| --- | --- | --- | --- | --- | --- | --- | --- | --- | --- | --- | --- | --- |
| 75 | Gunes & Bilek (2020) | 2020 | 316 public servants in Turkey | Turkey | Cross sectional Single source | Trait-like | Yes | 20-item R-UCLA loneliness scale | Antecedents Outcomes | - Women, unhappiness with the city social life, and lack of participation in social activities were positively related to loneliness. - Low income was positively related to loneliness - Married people were less lonely. - Those with relatives in the city were more lonely compared to those who had friends. - Job satisfaction was negatively related to loneliness | Digest of Middle East Studies | 0.4 |
| 76 | Hagen, Lai, & Goldmann (2022) | 2022 | Gallup poll of 80,000 nationally representative adults in the USA | US | Cross sectional Single source | State-like | No | Single item: Did you experience loneliness during a lot of the prior day? | Antecedent | - Unemployment was related to feelings of loneliness. | Preventive Medicine | 5.1 |
| 77 | Hardman, Lee, Llewellyn, & Walker (2022) | 2022 | Study 1: N = 759 health care professionals in 2020  Study 2: 925 health care professionals from around the  world, in 2021 | Global | Studies 1 and 2: Cross Sectional Single source | Trait-like | Yes | Studies 1 and 2: Single Item: I have felt lonely and isolated | Antecedents | - 28% vs 45% felt lonely (early vs later covid) - In both studies, more home workers, as opposed to office workers, felt lonely. | Medical Writing | NONE |
| 78 | Hart, Allen, Aubyn, & Mason (2023) | 2023 | 56 sex workers from various countries. | Australia | Cross sectional Single source | Trait-like | Yes | 20 item R-UCLA loneliness scale | Antecedent | - Internalized stigma of the job (HIV stigma) was correlated with loneliness. | Sexuality & Culture | 1.5 |
| 79 | Hirshberg et al. (2022) | 2022 | 662 school system employees | US | Experiment (experimental and control groups) Single source | Trait-like | Yes | NIH loneliness questionnaire (Cyranowski et al., 2006). | Intervention | - Assignment to the smartphone mediation app intervention resulted in greater reductions in loneliness and the effects persisted over the 3-month follow up. | Journal of Educational Psychology | 4.9 |
| 80 | Hofman, Overberg, Schoenmakers, & Adriaanse (2022) | 2022 | 7885 participants in the Netherlands | the Netherlands | Cross sectional Single source | Trait-like | No | 11 items, Social and emotional loneliness, De Jong Gierveld & Kamphuis (1985) (e.g. I miss having a really close friend) | Antecedents | - Having a paid job was related to lower emotional loneliness | Psychiatry research | 11.3 |
| 81 | Hong, Zhang, Li, Liu, & Zhou (2013) | 2013 | 1022 female sex workers in China | China | Cross sectional Single source | Trait-like | Yes | 20 item R-UCLA loneliness scale | Antecedents | - Experienced violence from clients was positively related to loneliness. | Plos One | 3.7 |
| 82 | Hu, Chen, & Ye (2023) | 2023 | 589 employees from China | China | Cross-sectional Single source | Trait-like | Yes | 4 item short form of R-UCLA loneliness scale adapted to work | Antecedents Outcomes Moderator | - Workplace loneliness mediated the relation between workplace ostracism and social cyberloafing. - Conscientiousness weakened the relationship. | Current Psychology | 2.8 |
| 83 | Huang & Chan (2022) | 2022 | 636 sexual minority individuals in Hong Kong | Hong Kong | Time lagged (2 waves; 1-year apart);  Single source | Trait-like | No | 6 item de Jong Gierveld & van Tilburg (2006) loneliness scale | Antecedent Outcome Moderator | - Sexual identity concealment negatively predicted well-being via loneliness. - Perceived LGBT friendliness was negatively correlated with loneliness. - Perceived LGBT friendliness in the workplace was not a moderator. - Loneliness was associated with lower levels of well   being. | Journal of Counseling Psychology | 3.9 |
| 84 | Ikeda, Hori, Sasaki, Komase, Doki et al. (2022) | 2022 | 1200 employees in Japan | Japan | Cross sectional Single source | Trait-like | Yes | 3 items, dichotomized. | Antecedents | - Cyberbullying at work was a risk factor for experienced loneliness. | BMC Public Health | 4.5 |
| 85 | Itzick & Kagan (2021) | 2021 | 394 social workers in Israel | Israel | Cross sectional Single source | Trait-like | Yes | 3 item short form of R-UCLA loneliness scale | Outcomes | - Loneliness was negatively related to life satisfaction. | International Social Work | 2.2 |
| 86 | Itzick, Kagan, & Ben-Ezra (2018) | 2018 | 501 social workers in Israel | Israel | Cross sectional Single source | Trait-like | Yes | 3 item short form of R-UCLA loneliness scale | Outcomes | - Loneliness was negatively related to the perceived meaning of life. | Journal of Social Work | 1.6 |
| 87 | Iwu, Okeke-Uzodike, Anwana, Iwu, & Esambe (2022) | 2022 | 18 academics in South Africa | South Africa | Qualitative Single source | Trait-like | Yes | Qualitative | Antecedents | - Academics during the pandemic missed interacting with students, and had only virtual interactions, leading to loneliness. | Challenges | NONE |
| 88 | Jelińska & Paradowski (2021) | 2021 | 804 higher education instructors from 92 countries | Global | Cross sectional Single source | Trait-like | Yes | 3 items to capture situational loneliness (e.g., I feel lonely) | Outcomes | - Loneliness during Covid-19 was related to negative affect. | Frontiers in Psychology | 3.8 |

|  | **Study** | **Year** | **Sample** | **Country** | **Study Design Characteristics** | **Trait or State** | **Working adults (Y/N)** | **Measure of Loneliness** | **Antecedent/ Outcome/ Moderator / Intervention** | **Primary Findings** | **Journal Name** | **Journal Impact Factor (2022)** |
| --- | --- | --- | --- | --- | --- | --- | --- | --- | --- | --- | --- | --- |
| 89 | Jha (2023) | 2023 | 382 employees and 97 supervisors in Indian organizations | India | Time lagged (2 waves)  Multi source (employees and  supervisors) | Trait-like | Yes | 20 item R-UCLA loneliness scale adapted to work | Outcome | - Workplace loneliness negatively predicted employee well being and psychological safety. - There were also indirect effects on performance via well being and psychological safety. | Industrial and Commercial Training | 1.4 |
| 90 | Jin & Ikeda (2023) | 2023 | 267 employees in China | China | Cross sectional Single source | Trait-like | Yes | The Wright et al. (2006) loneliness at work scale | Antecedents | - Servant leadership has an indirect effect on loneliness by having a positive relation with leader/colleague empathic communication, which in turn reduced feelings of workplace loneliness. | Behavioral Sciences | 2.6 |
| 91 | Joseph, Olasoji, Moss, & Cross (2022) | 2022 | 16 registered nurses trained in India and working in Australia | Indian workers in Australia | Qualitative Single source | Trait-like | Yes | Qualitative | Antecedents | - Having migrated to Australia was associated with feelings of loneliness in the initial days. They were separated from family and did not feel confident enough to interact with colleagues. Some overcame this by deliberately initiating interactions. They lacked commonality with colleagues. - Some relied on existing relationships back home to cope. - The authors found that migrant nurses experiencing loneliness and isolation adopted coping strategies such as engaging in hobbies and making new friends to   combat feelings of loneliness. | Journal of Transcultural Nursing | 2.1 |
| 92 | Jung, Song, & Yoon (2021) | 2021 | 292 employees working in hotels in S. Korea | South Korea | Cross sectional Single source | Trait-like | Yes | The Wright et al. (2006) loneliness at work scale | Outcomes Moderators | - Workplace loneliness reduced work engagement, which indirectly affected organizational commitment. - This relationship was moderated by coworker exchange such that the relation was weaker for those with more positive coworker exchanges. LMX did not   act as a moderator. | Sustainability | 3.9 |
| 93 | Kagan & GreenblattKimron (2021) | 2021 | 726 social workers in Israel | Israel | Cross sectional Single source | Trait-like | Yes | 3 item short form of R-UCLA loneliness scale | Outcomes | - Loneliness was positively related to psychological distress | Journal of Social Work | 1.6 |
| 94 | Kagan, Fridman, Shalom, & Melnikov (2018) | 2018 | 87 nurses | Israel | Cross sectional Single source | Trait-like | Yes | 20 item R-UCLA scale adapted to work. During the last month | Antecedents | - Working in isolation room contributed to loneliness. | Journal of Nursing Management | 5.5 |
| 95 | Kanbur & Kanbur (2020) | 2020 | 219 public sector employees in Turkey | Turkey | Cross sectional Single source | Trait-like | Yes | The Wright et al. (2006) loneliness at work scale | Antecedents | - Psychological empowerment and perceived internal status (e.g., feeling part of the organization) were related to loneliness. - Perceived internal status mediated the effects of   empowerment. | Business & Economics Research Journal | NONE |
| 96 | Katz and Katz (2002) | 2002 | 82 individuals with learning disabilities | Israel | Cross sectional Single source | Trait-like | Yes | The Chadsey-Rusch et al. (1992) worker loneliness questionnaire | Antecedents | - Work did not seem to affect loneliness – those who expressed loneliness and those who did not were similar in their occupations, were employed in similar jobs. - Those who reported loneliness also were not more handicapped. - Vocational integration did not seem to be related to   loneliness. | British Journal of Developmental Disabilities | 1.8 |
| 97 | Kazman, Bonner, Kegel, Nelson, & Deuster (2023) | 2023 | 743,057 U.S. Army personnel 3,478 U.S. Special Operation Forces | US | Single source | Trait-like | Yes | Loneliness scale developed for the US Army Global Assessment Tool | Outcomes | - Loneliness was significantly related to impairments in physical health and psychiatric problems. | Military Psychology | 1.1 |
| 98 | Keizer, Dykstra, & Poortman (2010) | 2010 | 338 women, 262 men from Netherland Kinship panel | the Netherlands | Time lagged (2 waves)  Single source | Trait-like | No | 11 item De Jong Gierveld and Kamphuis (1985) loneliness scale | Antecedents | - Transition to motherhood did not affect loneliness. - Men who became parents became lonelier. Men who married their cohabiting partner became lonelier, and this was responsible for the men becoming lonelier. - Increase or decrease in work hours were not related   to loneliness. | Journal of Family Psychology | 2.7 |
| 99 | Kloutsiniotis, Mihail, Mylonas, & Pateli (2022) | 2022 | 459 hotel employees in Greece | Greece | Cross sectional Single source | Trait-like | Yes | 2 items from the short version of UCLA-R, adapted to work | Antecedents Outcome | - Transformational leadership was negatively related to workplace loneliness. - Workplace loneliness was positively related to   burnout | International Journal of Hospitality Management | 11.7 |

|  | **Study** | **Year** | **Sample** | **Country** | **Study Design Characteristics** | **Trait or State** | **Working adults (Y/N)** | **Measure of Loneliness** | **Antecedent/ Outcome/ Moderator / Intervention** | **Primary Findings** | **Journal Name** | **Journal Impact Factor (2022)** |
| --- | --- | --- | --- | --- | --- | --- | --- | --- | --- | --- | --- | --- |
| 100 | Kolbaek, Gil, Schmidt, Speed & Ostergaard (2023) | 2023 | 613 adults in Denmark | Denmark | Time lagged (2 waves)  Single source | Trait-like | No | Single item measure asking for the cause of their mental deterioration. | Outcomes | - Participants commonly reported loneliness as the cause for deterioration in mental health during the Covid-19 pandemic. | Nordic Journal of Psychiatry | 2.3 |
| 101 | Kong, Chen, & Chen (2023) | 2023 | 388 migrant workers in China | China | Time lagged (3 waves)  Single source | Trait-like | Yes | Single item “felt lonely?” rarely to most or all the time. | Outcomes | - Loneliness predicted smoking rates | Behavioral Sciences | 2.6 |
| 102 | Korumaz (2016) | 2016 | 7 elementary school principals in Turkey | Turkey | Qualitative Single source | Trait-like | Yes | Qualitative | Antecedents | - Loneliness was prevalent among school principals. - Organizational climate was a key culprit in feelings of loneliness. - One of the themes uncovered in this qualitative paper, “professional effort”, can be seen as lonely principals’ adaptive response to loneliness. The theme of professional effort suggests that principals actively seek ways to overcome loneliness, such as planning social events and fostering closer relationships. This implies that loneliness can serve as a motivator for positive   change in the workplace. | South African Journal of Education | 0.8 |
| 103 | Kotera, Ozaki, Miyatake, Tsunetoshi, Nishikawa, & Tanimoto (2021) | 2021 | 142 medical workers and 138 individuals from general population | Japan | Cross sectional Single source | Trait-like | No | 3 item short form of R-UCLA loneliness scale | Antecedents Outcomes | - Medical workers reported higher loneliness compared to the general population - Loneliness was associated with mental health problems in medical workers and the general   population. | Current Psychology | 2.8 |
| 104 | Kuriakose, Sreejesh, Wilson, & Anusree (2019) | 2019 | 554 IT Employees | India | Cross sectional Single source | Trait-like | Yes | 3 item short form of R-UCLA loneliness scale, adapted to work | Antecedents Outcomes Moderators | - Loneliness was negatively related to well-being and social support - Loneliness was positively related to relationship, process and task-based peer conflict. - Loneliness mediated the relation between conflict and well being. - Perceived social support moderated conflict -   loneliness relationship. | International Journal of Conflict Management | 3.3 |
| 105 | Kuriakose, Tresita, & Bishwas (2023) | 2023 | 243 hotel workers in India | India | Time lagged (2 waves)  Single source | Trait-like | Yes | 3 item short form of R-UCLA loneliness scale, adapted to work | Antecedents Outcomes | - Workplace incivility predicted loneliness - Workplace Loneliness was negatively related to well being - Social support weakened the relation between   incivility and loneliness. | International Journal of Contemporary Hospitality Management | 11.1 |
| 106 | Lalloo, Lewsey, Katikireddi, Macdonald, Campbell, & Demou (2022) | 2022 | 4093 IT workers from the UK biobank study | UK | Cross sectional Single source | Trait-like | Yes | Combined “Do you often feel lonely?” (no = 0, yes = 1) and “How often are you able to confide in someone close to you?” (0 = almost daily to once  every few months; 1 = never or almost never). | Antecedent | - IT professionals and technicians had greater odds of self-reported loneliness compared to IT managers. | Annals of Work Exposures and Health | 2.6 |
| 107 | Lasgaard, M., Friis, K., & Shevlin,  M. (2016) | 2016 | 33,285 Danish individuals  aged 16-102 years | Denmark | Cross sectional Single source | Trait-like | No | 3 item short version of the UCLA loneliness scale | Antecedent | - The relation between loneliness and age had a shallow U-shaped distribution. - Ethnic minority status, living alone, and psychiatric treatment were strongly associated with severe loneliness - Being female, having a low educational level, and living in a deprived area were only associated with loneliness in adolescence/emerging adulthood. - Receiving disability pensions and living alone (i.e., divorced), on the other hand, were strongly associated with loneliness in early and middle adulthood and   young-old age. | Social Psychiatry and Psychiatric Epidemiology: The International Journal for Research in Social and Genetic Epidemiology and Mental Health Services | 4.4 |
| 108 | Lam & Lau (2012) | 2012 | 532 teacher-supervisor dyads in Macao | China | Cross sectional Multi-source | Trait-like | Yes | 4 item short form of R-UCLA loneliness scale, adapted to work | Outcomes | - Workplace loneliness was negatively related to supervisor-rated in-role performance and OCBI, but not OCBO. - LMX was a mediator of this relationship, but the   organization-member exchange was not. | International Journal of Human Resource Management | 5.6 |

|  | **Study** | **Year** | **Sample** | **Country** | **Study Design Characteristics** | **Trait or State** | **Working adults (Y/N)** | **Measure of Loneliness** | **Antecedent/ Outcome/ Moderator / Intervention** | **Primary Findings** | **Journal Name** | **Journal Impact Factor (2022)** |
| --- | --- | --- | --- | --- | --- | --- | --- | --- | --- | --- | --- | --- |
| 109 | Lawrence, Matthieu, & Robertson-Blackmore (2017) | 2017 | 67 veterans with traumatic brain injury and 273 without traumatic brain injury | USA | pre-post design Intervention Single source | State-like | Yes | 20 item R-UCLA, referencing past month | Intervention | - Veterans with traumatic brain injury participated in a volunteering program at a nonprofit of their choosing and received a stipend for 6 months. The pre-post design showed reduction in loneliness. - Pre-post changes did not differ based on whether the   veteran had brain injury | Military Medicine | 1.2 |
| 110 | LeCheminant, Merrill, & Masterson (2017) | 2017 | 1873 employees of a public school district, surveyed 3 times | US | Time lagged (3 waves)  Single source | Trait-like | Yes | Single Item: # of days in the last 3 months employee’s daily routine was disrupted by loneliness | Intervention | - A wellness program implemented in the study had no effects on loneliness | Health Promotion Practice | 1.9 |
| 111 | Lee (2022) | 2022 | 9944 people over 65 in the USA | US | Cross sectional Single source | Trait-like | No | 3 item short form of R-UCLA loneliness scale | Antecedents | - Charity work was negatively related to loneliness - Self-efficacy and perceived self-control were negatively related to loneliness - Charity work was related to perceived control and social self-efficacy, which in turn were related to lower   loneliness. | Activities, Adaptation & Aging | 2.8 |
| 112 | Leeflang, Klein-Hesselink, & Spruit (1992) | 1992 | 298 employed and 298 unemployed male respondents in urban Netherlands, and 100 employed and 100 unemployed in rural Netherlands | the Netherlands | Cross sectional Single source | Trait-like | No | 11 item De Jong Gierveld and Kamphuis (1985) loneliness scale | Antecedents Outcomes | - Loneliness was affected by health, friends and acquaintances, money worries, and disadvantageous consequences of unemployment. - Loneliness predicted depressive symptoms among urban and rural unemployed. | Social Science & Medicine | 5.4 |
| 113 | Li, Cui, Efstathiou, Li, & Guo (2022) | 2022 | 20 redeployed healthcare workers in China during Covid | China | Qualitative Single source | Trait-like | Yes | Qualitative | Antecedents | - Participants redeployed to Wuhan isolation wards during the pandemic experienced loneliness because of being unable to talk to anyone. | Plos One | 3.7 |
| 114 | Lin (2023) | 2023 | 3772 Canadian individuals in a population survey during Covid-19 pandemic | Canada | Cross sectional Single source | Trait-like | No | Pandemic Loneliness - 3 item short form of  R-UCLA (e.g. In  general, how often have you felt the following  since the start of the COVID-19  pandemic?) | Antecedent Outcomes | - Teenagers, women, those who lived alone, with limited social networks, binge drinking, and smoking cannabis were at greater risk of loneliness. - Absence from work due to Covid was associated with severe pandemic loneliness. - Immigrants who experienced job precarity during the pandemic were most at risk of loneliness. - Severe loneliness was independently associated with greater odds of mental health help-seeking and unmet   mental health needs. | Journal of Affective Disorders | 6.6 |
| 115 | Luck, Doucet, & Luke (2022) | 2022 | 23 individuals living with chronic disease in Canada | Canada | Qualitative; Single source | Trait-like | No | Qualitative | Antecedent | - Challenges associated with additional demands on their time, new roles they had to balance, and changed routine contributed to a sense of loneliness | Journal of Occupational Science | 2.4 |
| 116 | Luhmann & Hawkley (2016) | 2016 | 16132 individuals from Germany | Germany | Cross sectional Single source | Trait-like | No | 3 item short form of R-UCLA loneliness scale | Antecedents | - Loneliness was highest among those who do not work at all, and lowest among those with full time jobs. - Once income is controlled for, the relation disappeared. - The relation was reversed after controlling for other   covariates such as gender, education, relationship status. | Developmental Psychology | 4 |
| 117 | Luk et al. (2023) | 2023 | 463 adults enrolled in alcohol abuse study (includes those with and without alcohol abuse) | US | Cross sectional Single source | Trait-like | No | The UCLA  loneliness scale (Russell, 1996) | Antecedent | - During Covid, financial stress (reduced pay, not paid at all), work interruptions (increased or reduced hours, not being able to work) were among the important predictors of loneliness. | American Psychologist | 16.4 |
| 118 | Mann & Holdsworth (2003) | 2003 | Study 1: 12 journalists  Study 2: 62 journalists | UK | Study 1: Qualtiative Single Source Study 2: Quantitative  Single Source | Trait-like | Yes | Qualitative Study | Antecedent | - Results suggest a negative emotional impact of teleworking, particularly in terms of such emotions as loneliness, irritability, worry and guilt | New Technology Work and Employment | None |
| 119 | Mann, Varey & Button (2000) | 2020 | 14 adults in the UK working as tele-workers | UK | Qualtiative Single Source | Trait-like | Yes | Qualitative Study | Antecedent | - Social isolation due to telework is linked to feelings of loneliness | Journal of Managerial Psychology | 3.2 |
| 120 | Martinez, Glover, Ota, Long, & Ureta Viroga (in press) | 2023 | 33 pre-tenure faculty of color in the U.S. | US | Qualitative Single source | Trait-like | Yes | Qualitative Study | Antecedent | - Stress and the consuming nature of academia contribute to feelings of loneliness. - Being a minority woman limited the usefulness of   socializing. | Journal of Diversity in Higher Education | 2.4 |

|  | **Study** | **Year** | **Sample** | **Country** | **Study Design Characteristics** | **Trait or State** | **Working adults (Y/N)** | **Measure of Loneliness** | **Antecedent/ Outcome/ Moderator / Intervention** | **Primary Findings** | **Journal Name** | **Journal Impact Factor (2022)** |
| --- | --- | --- | --- | --- | --- | --- | --- | --- | --- | --- | --- | --- |
| 121 | Matthieu, Lawrence, & Robertson-Blackmore (2017) | 2017 | 346 veterans | USA | pre-post design Intervention Single source | State-like | Yes | 20 item R-UCLA referencing past month | Intervention | - Veterans felt less lonely 6 months after taking part in a volunteering program. - Screening positive for PTSD resulted in greater reductions in loneliness. | Psychiatry research | 11.3 |
| 122 | McDowell, Herring, Lansing, Brower, & Meyer (2021) | 2021 | 2301 US adults during Covid-19 | US | Cross sectional Single source | Trait-like | Yes | 3 item short form of R-UCLA loneliness scale | Antecedents | - Those who switched to work-from-home and those who lost their job did not report higher loneliness relative to those with no job changes. | Frontiers in Psychology | 3.8 |
| 123 | McFadden & Crowley-Henry (2018) | 2018 | 29 LGBT employees in Ireland | Ireland | Qualitative Single source | Trait-like | Yes | Qualitative | Antecedents Moderator | - LGBT employees may experience isolation at work because of their minority status. - LGBT employee networks can give employees voice   and moderate this relationship. | International Journal of Human Resource Management | 5.6 |
| 124 | Meese, Colón-López, Singh, Burkholder, & Rogers (2021) | 2021 | 1130 employees of a healthcare system in the USA | US | Cross sectional Single source | Trait-like | Yes | Single item | Outcomes | - Loneliness was related to distress. | Journal of Healthcare Management | 1.8 |
| 125 | Megalakaki & Kokou-Kpolou (2022) | 2022 | 556 participants in France during Covid-19 | France | Cross sectional Single source | Trait-like | No | 3 item short form of R-UCLA loneliness scale | Outcome Moderator | - Loneliness was associated with anxiety, depression, and insomnia, and these relations were mediated by Covid anxiety, - Loneliness - outcome relation was stronger for those who lost their job during the pandemic, those who lived alone, those with preexisting diseases, and those were   were worried about covid. | Current Psychology | 2.8 |
| 126 | Moens, Baert, Verhofstadt, & Van Ootegem (2021) | 2021 | 1358 employees in Belgium | Belgium | Cross sectional Single source | Trait-like | Yes | 4 item short form of R-UCLA loneliness scale | Antecedent Outcome | - Temporary work was positively related to loneliness, and indirectly related to job satisfaction. - These relations were not moderated by job tenure. | Plos One | 3.7 |
| 127 | Mokros, Świtaj, Bieńkowski, Święcicki, & Sienkiewicz-Jarosz (2022) | 2022 | 1795 working adults in Poland (nationally representative sample) | Poland | Cross sectional Single source | Trait-like | Yes | Single Item: “How often do you feel lonely?” | Outcomes | - Loneliness was related to work inefficiency and absence from work, with effect sizes larger than depression. - After control variables were accounted for, no effects   were found on turnover intentions. | International Archives of Occupational and Environmental Health | 3 |
| 128 | Morris (2020) | 2020 | 10154 working older adults from 14 countries | Global | Time lagged (2 years)  Single source | Trait-like | Yes | 3 item short form of R-UCLA loneliness scale | Outcomes | - Loneliness was predictive of the onset of work disability two years later. - Depression mediated this relationship. | Journal of Aging and Health | 2.8 |
| 129 | Morrish & Medina-Lara (2021) | 2021 | Meta analysis of unemployment – loneliness literature | Global | Meta-analysis | Trait-like | No | Meta analysis | Antecedents | - Unemployment was related to feeling lonely. - The relationship was stronger for those between 30-34 and 50-59 years old. | Social Science & Medicine | 5.4 |
| 130 | Morrish, Mujica-Mota, & Medina-Lara (2022) | 2022 | 19566 adults of a population survey in Wave 1, 18833 in Wave 2 in the UK | UK | Time lagged Single source | Trait-like | No | Single Item: “How often do you feel lonely?” | Antecedents Outcomes Moderator | - Experiencing loneliness increased the risk of unemployment at a later date by 17.5%. - There was also evidence that unemployment x physical health interacted such that the relationship was stronger for those who were permanently sick or disabled. - There was also evidence of a bidirectional relationship such that, unemployment increased the risk   of loneliness at a later date by 7.8%. | BMC Public Health | 4.5 |
| 131 | Mugoya, Hooper, Tomek, Dalmida, Bolland, Ufomadu, & Bolland (2018) | 2018 | 876 adult caregivers of adolescents | US | Cross-Sectional Single source | Trait-like | No | 20-item R-UCLA loneliness scale | Antecedents Moderator | - Employed people were less likely to feel lonely. - Disabling and nondisabling pain were related to loneliness, which in turn was related to depression. - Employment status moderated the relation between disabling pain and loneliness such that, the relation was   more positive for employed people. | Clinical Rehabilitation | 3 |
| 132 | Mullins, Sheppard, & Andersson (1991) | 1991 | 1005 people in Sweden | Sweden | Cross sectional Single source | Trait-like | No | Single Item: Do you feel loneliness is for you personally a very serious problem, a somewhat serious problem, or hardly a problem at all? | Antecedents | - Those 65 and older, and those who were retired were more socially isolated but not necessarily lonely. - Those who had health problems and those whose income was insufficient were lonelier. | Journal of Applied Gerontology | 3 |
| 133 | Negi, Siegel, Sharma, & Fiallos (2021) | 2021 | 37 Immigrant day Laborers | US | Qualitative Single source | Trait-like | Yes | Qualitative | Antecedents Outcomes | - Racism, dehumanization, and structural vulnerability contributed to loneliness among day laborers. - Outcomes included drug use and sexual risk-taking. | Social Science & Medicine | 5.4 |

|  | **Study** | **Year** | **Sample** | **Country** | **Study Design Characteristics** | **Trait or State** | **Working adults (Y/N)** | **Measure of Loneliness** | **Antecedent/ Outcome/ Moderator / Intervention** | **Primary Findings** | **Journal Name** | **Journal Impact Factor (2022)** |
| --- | --- | --- | --- | --- | --- | --- | --- | --- | --- | --- | --- | --- |
| 134 | Neto & Neto (2023) | 2023 | 402 full-time employees in Portugal | Portugal | Quantiative Single source | Trait-like | Yes | 6-item loneliness scale developed for this study | Antecedents Outcomes | - Loneliness demonstrated a negative correlation with self-esteem. - Loneliness demonstrated a significant negative correlation with the will to live, job satisfaction and life   satisfaction. | International Journal of Applied Positive Psychology | NONE |
| 135 | Nichols & McBride (2017) | 2017 | 4 first-year educational leaders | US | Qualitative Single source | Trait-like | Yes | Qualitative | Antecedents | - Examined loneliness experienced when transitioning from teacher to leader roles. - Some new leaders experienced loneliness and accepted this as a part of the job. The ones who did not experience it had faced promotions many times in the past. - Changes in relationships with former peers are a   culprit. | College Student Journal | NONE |
| 136 | Nie, Chen, & Yu (2023) | 2023 | 243 employee-colleague dyads in China | China | Time lagged (2 weeks)  Multi-source | Trait-like | Yes | 4 item short form of R-UCLA loneliness scale, adapted to work | Outcomes Moderator | - Workplace loneliness was related to employee territorial behaviors (peer rated) via self serving cognitions. - Self sacrificial leadership was a moderator. There was a positive relation between WL and SSC when SSL   was low, and no significant relationship when high. | International Journal of Conflict Management | 3.3 |
| 137 | Nilsen & Anderssen (2014) | 2014 | 20 working adults with chronic pain | Norway | Qualitative Single source | Trait-like | Yes | Qualitative | Antecedents Outcomes | - Participants characterized having to stay home because of their pain as lonely. - Pain isolated them from others and contributed to loneliness. Unpredictability made it difficult to keep to agreements. - Paid work helped cope with loneliness. | Work: Journal of Prevention, Assessment & Rehabilitation | 2.3 |
| 138 | Nilvarangkul, Rungreangkulkij, & Wongprom (2010) | 2010 | 70 Laotian migrant workers in Thailand | Laotian workers in Thailand | Qualitative Single source | Trait-like | Yes | Qualitative | Antecedents | - Loneliness was a contributor to stress for migrant workers. - Laotians were able to communicate with Thai citizens and were still lonely. - Living far from home, having no one to trust and talk   to, or share activities with created these conditions. | Journal of Immigrant and Minority Health | 1.9 |
| 139 | Ogueji, Agberotimi, Adesanya, & Gidado (2021) | 2021 | 66 employed and unemployed people in Nigeria | Nigeria | Qualitative Single source | Trait-like | No | Qualitative | Antecedents | - Inability to have colleagues around causes loneliness. | Analyses of Social Issues and Public Policy | 1.5 |
| 140 | Okamura, Tsuda, & Matsuishi (2011) | 2011 | 90 female full-time workers | Japan | Cross sectional Multi source | Trait-like | Yes | 20-item R-UCLA loneliness scale | Outcomes | - Loneliness was related to higher work stress and higher cortisol awakening response on the weekends. - In the high loneliness group, cortisol awakening did not differ between workdays and weekends. There were   differences in the low loneliness group. | Japanese Psychological Research | 0.8 |
| 141 | Olds, Sprod, Ferrar, Burton, Brown, van Uffelen, & Maher (2016) | 2016 | Trait-like | Australia | Time lagged (4 waves)  Single source | Trait-like | Yes | 20-item R-UCLA loneliness scale | Outcomes | - Loneliness was negatively related to enjoyment of life activities among people in pre-retirement. | Age & Ageing | 6.7 |
| 142 | Ong (2022) | 2022 | Study 1: 442 working adults  Study 2: Experiment with 206 participants  Study 3: 269 adults from Prolific | US | S1: Time lagged (2 waves)  Single source  S2: Experiment Single source  S3: Time lagged (2 waves)  Single source | Trait-like | Study 1: Yes Study 2: No Study 3: No | Study 1: 3 item Revised UCLA Study 2: 3 item Revised UCLA referencing the group  Study 3: 3 item Revised UCLA referencing the work | Antecedent | - Women experienced a sense of loneliness after being promoted to management. Men did not. - This effect was because women experienced lower authenticity after being promoted. - Women who are managers experience loneliness, but men did not. | Journal of Applied Psychology | 9.9 |
| 143 | Ozcelik & Barsade (2018) | 2018 | 672 employees, 114 supervisors | US | Time lagged Multi-source | Trait-like | Yes | 20-item R-UCLA loneliness scale adapted to work | Outcomes Moderator | - Loneliness predicted employee approachability (coworker-rated) and lower commitment to the organization, and indirectly related to supervisor-rated job performance. - Moderators included a strong culture of compassionate love (weakened the relation between loneliness and commitment), high coworker loneliness (strengthened the relationship), the strength of culture of   anger (strengthened the relationship). | Academy of Management Journal | 10.5 |

|  | **Study** | **Year** | **Sample** | **Country** | **Study Design Characteristics** | **Trait or State** | **Working adults (Y/N)** | **Measure of Loneliness** | **Antecedent/ Outcome/ Moderator / Intervention** | **Primary Findings** | **Journal Name** | **Journal Impact Factor (2022)** |
| --- | --- | --- | --- | --- | --- | --- | --- | --- | --- | --- | --- | --- |
| 144 | Palacios Picos, Pinedo González, & de la Iglesia Gutiérrez (2018) | 2018 | 146 female sex workers in Spain | Spain | Cross-sectional Single source | Trait-like | Yes | Short version of the Di Tommaso et al. (2004) social and emotional loneliness scale | Outcomes | - Loneliness was negatively related to psychological health. - Psychological health was related to perceived quality of life, biosocial and environmental health, negatively   related to drug use, and negatively related to anxiety. | Health Care for Women International | 1.4 |
| 145 | Peng, Chen, Xia, & Ran (2017) | 2017 | 67 leaders and 261 subordinates in China | China | Cross sectional Multi-source | Trait-like | Yes | The Wright et al. (2006) loneliness at work scale | Outcomes | - Workplace loneliness was negatively related to manager-rated creativity. - LMX partially mediated this relationship. - The Loneliness-LMX relationship was more negative when leader compassion was low, and non-significant when high. | Personality and Individual Differences | 4.3 |
| 146 | Penton, Bowling, Vafeiadou, Hammond, Bird & Banissy (2023) | 2023 | 273 autistic adults and 273 non-autistic adults | United Kingdom | Cross sectional Single source | Trait-like | Yes | 20 item R-UCLA loneliness scale | Antecedents | - For both employed autistic individuals and   non-autistic individuals, negative attitudes toward touch at work were related to higher levels of loneliness.   - For both employed autistic individuals and   non-autistic individuals, attachment-related avoidance (e.g., seeing others as intrusive) and attachment-related anxiety (e.g., feeling you need others and fearing rejection) were related to higher levels of loneliness. | Journal of Autism and Developmental Disorders | 4.2 |
| 147 | Petrovski & Gleeson (1997) | 1997 | 31 workers with mild intellectual disability | US | Cross sectional Single source | Trait-like | Yes | The Chadsey-Rusch et al. (1992) worker loneliness questionnaire; interviews | Antecedents Outcomes | - There was a negative correlation between job satisfaction and loneliness. - Women perceived more loneliness at work than men. | Journal of Intellectual and Developmental Disability | 1.3 |
| 148 | Phillips, Becker, & Gonzalez (2021) | 2021 | 43 oncology nurses in the USA | US | Cross sectional Single source | Trait-like | Yes | The 20 item  R-UCLA loneliness scale | Outcomes | - Loneliness predicted burnout. - Loneliness negatively predicted compassion satisfaction (pleasure derived from doing one’s job well). | Clinical Journal of Oncology Nursing | 1.1 |
| 149 | Pinedo González, Palacios Picos, & de la Iglesia Gutiérrez (2021) | 2021 | 146 sex workers in Spain | Spain | Cross sectional Single source | Trait-like | Yes | 4 item, family loneliness (I feel close to my family etc) | Antecedents Outcomes | - Psychological violence and physical violence were positively related to loneliness - Self-esteem was negatively related to loneliness - Loneliness was related to physical health, psychological health, and drug use. | Journal of Interpersonal Violence | 2.5 |
| 150 | Poulsen & Ipsen (2017) | 2017 | 17 participants from 4 companies engaged in distance work | Denmark | Qualitative Single source | Trait-like | Yes | Qualitative | Antecedents | - The study examined organizations where customers dictated where and where employees worked, and employees did not have access to their own managers due to distance. - A downside of this arrangement emerged as   loneliness experienced by employees. | Safety Science | 6.1 |
| 151 | Psenka et al. (2020) | 2020 | 268 program directors in family medicine programs | US | Cross sectional Single source | Trait-like | Yes | 3 item short form of R-UCLA loneliness scale | Antecedents | - Nearly 1/3 of program directors reported feeling a sense of isolation. | Family Practice | 2.2 |
| 152 | Ramana, Singh, Rupani, Mukherjee, & Mohapatra (2023) | 2023 | 12 migrant construction workers in India during Covid-19 | India | Qualitative Single source | Trait-like | Yes | Qualitative | Antecedents | - Migrant workers reported loneliness during Covid-19 | Work: Journal of Prevention, Assessment & Rehabilitation | 2.3 |
| 153 | Regmi, Aryal, van Teijlingen, Simkhada, & Adhikary (2020) | 2020 | 15 interviews with returnee Nepali migrants and key informants | Nepal | Qualitative Single Source | Trait-like | No | Qualitative | Antecedents Outcomes | - Loneliness was related to mental health problems. - Inability to communicate with others is a common reason for loneliness. | Journal of Immigrant and Minority Health | 1.9 |
| 154 | Rockmann & Pratt (2015) | 2015 | interviews with 29 employees doing distributed work in a tech company | USA | Qualitative Single source | State-like | Yes | Qualitative | Antecedents | - Working in a distributed office resulted in a sense of loneliness because of the absence of social interactions with colleagues. - Even when one was at the office, the "relevant others" or close colleagues were missing. The office became a   lonelier place even if there were people there. | Academy of Management Discoveries | 6.3 |
| 155 | Sapmaz (2023) | 2023 | 204 employees in Turkey | Turkey | Qualitative Single source | Trait-like | Yes | The Wright et al. (2006) loneliness at work scale | Outcomes | - Loneliness at work was significantly correlated with fear of missing out on relationships at work. - Loneliness at work was negatively related to feelings of belongingness, which in turn were related to fear of   missing out. | Journal of Educational Technology and Online Learning | NONE |
| 156 | Sasaki, Kuroda, Tsuno, Imamura, & Kawakami (2021) | 2021 | 875 working adults in Japan | Japan | Time lagged (3 waves)  Single source | Trait-like | Yes | Single item: “I feel lonely” | Outcomes | - Loneliness increased during the pandemic. - Loneliness predicted suicidal ideation. | BJPsych Open | 5.4 |

|  | **Study** | **Year** | **Sample** | **Country** | **Study Design Characteristics** | **Trait or State** | **Working adults (Y/N)** | **Measure of Loneliness** | **Antecedent/ Outcome/ Moderator / Intervention** | **Primary Findings** | **Journal Name** | **Journal Impact Factor (2022)** |
| --- | --- | --- | --- | --- | --- | --- | --- | --- | --- | --- | --- | --- |
| 157 | Satilmis, Oznacar, Uzunboylu, & Yılmaz (2018) | 2018 | 457 teachers in Turkey | Turkey | Qualitative Single source | Trait-like | Yes | The Wright et al. (2006) loneliness at work scale | Antecedents | - Organizational cynicism, life satisfaction (-), and general cynicism were related to workplace loneliness (emotional deprivation dimension). - Life satisfaction and organizational cynicism were related to the loneliness social companionship   dimension. | Quality & Quantity: International Journal of Methodology | NONE |
| 158 | Şentürk, Sağaltıcı, Geniş, & Günday Toker (2021) | 2021 | 459 remote workers in Turkey during Covid 19 | Turkey | Cross sectional Single source | Trait-like | Yes | Single item work loneliness | Outcomes | - Work loneliness was related to stress and depression. | Work: Journal of Prevention, Assessment & Rehabilitation | 2.3 |
| 159 | Shin, Park, Amano, Kwon, & Kim (2020) | 2020 | 2055 participants from the 2014 Health and Retirement survey | US | Cross sectional Single source | Trait-like | No | 11 items from R-UCLA scale | Antecedents | - Involuntary retirement predicted loneliness. - Social support alleviated loneliness. - Among retired individuals, women, married or partnered individuals felt less lonely. - Self-rated health was negatively related to loneliness. | Journal of Applied Gerontology | 3 |
| 160 | Silard & Wright (2022) | 2022 | 28 managers and 235 employees from a horticultural company in Mexico | Mexico | Cross sectional Multi-source | Trait-like | Yes | 20 item R-UCLA loneliness scale adapted to work | Antecedents | - There was no difference between managers and employees in loneliness experienced. - Emotional connection and mutuality predicted loneliness in employees, but not in managers (but also,   manager sample size was small). | Management Research Review | 2.6 |
| 161 | Silman & Dogan (2013) | 2013 | 326 Academics in Turkey and Northern Cyprus | Turkey and Northern Cyprus | Cross sectional Single source | Trait-like | Yes | The Wright et al. (2006) loneliness at work scale | Antecedents | - Social intelligence (social information processing, social skills, and social awareness) were negatively related to the social companionship dimension of loneliness. Social skills and social awareness were negatively related to the emotional deprivation dimension. | Spanish Journal of Psychology | 2.3 |
| 162 | Slepian & Jacoby-Senghor (2021) | 2021 | Study 4: 179 participants from Mturk | US | Cross sectional Single source | Trait-like | Yes | 3 item short form of R-UCLA loneliness scale | Antecedents | - Feeling a lack of belongingness at work predicted loneliness through the feeling of inauthenticity. | Social Psychological and Personality Science | 5.7 |
| 163 | Smith-Appelson, Reynolds, & Grzywacz (2021) | 2021 | 290 immigrant farmworkers | US | Cross sectional Single source | Trait-like | Yes | 5 items from the R-UCLA scale | Antecedents Outcomes | - Older women immigrant workers, those with no formal schooling, and those with food insecurity were especially lonely. - Loneliness did not predict self-reported health. | Sociological Inquiry | 1.9 |
| 164 | Smith, Ford, & Steffen (2019) | 2019 | 78 firefighters in a 21-day diary study | US | ESM 21 Day Daily Diary Measures Single Source | State-like | Yes | Single item: “How much did you feel lonely during this day?” | Antecedents | - Daily stress and partner stress were associated with increased loneliness. - Individuals who were mindful experienced lower   increases in loneliness in response to total stress. | Mindfulness | 3.6 |
| 165 | Soler-Gonzalez, San-Martín, Delgado-Bolton, & Vivanco (2017) | 2017 | 433 physicians and nurses in Spain | Spain | Cross sectional Single source | Trait-like | Yes | DiTommaso et al. (2004) social and emotional loneliness scale (family, romantic, and social loneliness dimensions) | Outcomes | - An absence of positive human connection (=Loneliness) is related to perceptions of occupational well being, consisting of somatization, exhaustion, and alienation dimensions. | Frontiers in Psychology | 3.8 |
| 166 | Solomon, Mikulincer, & Hobfoll (1986) | 1986 | 716 soldiers | Israel | Cross sectional Single source | Trait-like | Yes | Single item: Have you felt lonely during battle | Antecedents | - Lack of social support from officers was found to be related to greater feelings of loneliness and greater likelihood of combat stress in soldiers. - Lack of social support from buddies was found to be related to greater loneliness. - Intensity of battle was also found to be related to   greater feelings of loneliness. | Journal of Personality and Social Psychology | 7.6 |

|  | **Study** | **Year** | **Sample** | **Country** | **Study Design Characteristics** | **Trait or State** | **Working adults (Y/N)** | **Measure of Loneliness** | **Antecedent/ Outcome/ Moderator / Intervention** | **Primary Findings** | **Journal Name** | **Journal Impact Factor (2022)** |
| --- | --- | --- | --- | --- | --- | --- | --- | --- | --- | --- | --- | --- |
| 167 | Spiro et al. (2021) | 2021 | 385 performing art professionals in the UK | UK | Cross sectional Single source | Trait-like | Yes | 3 item short form of R-UCLA loneliness scale | Antecedents | - 63% of participants reported feeling lonelier during the pandemic. - Age and physical activity during the pandemic were negatively related to loneliness, whereas perceived financial hardship and living alone was positively related. - This research examined the impact of the COVID-19 lockdown on performing arts professionals in the UK (63% of the sample reported being lonelier than before the crisis) and found that despite the challenges, some individuals reported positive outcomes, such as having more time for themselves, reduced pressure, and opportunities for new skills and activities (sub-themes 5.1 and 5.2). This indicates loneliness providing mental space by diminishing cognitive distractors, thereby   facilitating growth. | Frontiers in Psychology | 3.8 |
| 168 | Spruijt, Cronin, Udeorji, Nazir, Shehu, Poix, Villanueva, Jansen, Huitema, Suurmond, & Fiekert (2023) | 2023 | 53 healthcare workers from Ireland, Nigeria, Netherlands, Pakistan, and the Philippines | Ireland, Nigeria, Netherlands, Pakistan, and the Philippines | Qualitative Single source | Trait-like | Yes | Qualitative | Antecedents | - Caring for covid-19 patients resulted in loneliness due to stigmatization | Plos One | 3.7 |
| 169 | Stefanowski, Mokros, Sienkiewicz-Jarosz, Baka, Bugajska, & Switaj (2023) | 2023 | 460 firefighters from Poland | Poland | Cross sectional Single source | Trait-like | Yes | 11 item De Jong Gierveld and Kamphuis (1985) loneliness scale | Antecedents Outcomes | - Loneliness was positively related to emotional exhaustion - Loneliness was positively related to disengagement - Loneliness was positively related to work ability - Loneliness mediated the relation between exhaustion, disengagement, and work ability. | Advances in Psychiatry and Neurology | NONE |
| 170 | Surkalim, Clare, Eres, Gebel, Bauman, & Ding (2023) | 2023 | 18,000+ participants in a Health and Retirement Survey in the U.S. | US | Cross sectional Single source | State-like | No | Much of the time during the past week…) You felt lonely: (1) Yes; (2) No.” | Antecedents | - Working participants reported lowest loneliness compared to unemployed and retired. Unemployed had the highest. - Retired and unemployed participants reported a 41% and 48% higher risk for episodic loneliness and an 88% and 96% higher risk for sustained loneliness compared   to their working counterparts, respectively. | Journals of Gerontology: Social Sciences | 6.2 |
| 171 | Sylvia et al. (2021) | 2021 | Clinicians in Massachusetts health care system | US |  | Trait-like | Yes | 2 items from the R- UCLA scale | Moderator Intervention | - Participants who were lonely benefited more from a resiliency intervention in the form of stress coping. | Journal of Affective Disorders | 6.6 |
| 172 | Tam, Zhou, Qiao, Li, & Shen (2022) | 2022 | 410 sex workers in China | China | Cross sectional Single source | Trait-like | Yes | 8 items from the  R-UCLA loneliness scale | Antecedents Outcomes | - Loneliness was treated as an indicator of psychological distress. - Mindfulness was negatively correlated with loneliness - Somatic symptoms (pain, cardiopulmonary symptoms, gastrointestinal symptoms) were positively   correlated with loneliness. | Applied Psychology: Health and Well Being | 6.9 |
| 173 | Tang et al. (2023) | 2023 | Study 1 = 166 engineers, their coworkers, and family members, in a biomedical firm in Taiwan  Study 2= 120 real estate consultants in Indonesia Study 3 = 214 adults from the US (Prolific sample)  Study 4 = 294 employees from a Malaysian company | Taiwan, Malaysia, Indonesia, and US | S1: Time lagged (3 waves, separated by 1 week)  Multi-source.  S2: Experiment Single source  S3: Experiment Single source  S4: Experiment Single source | State-like | Yes | Studies 1-4: 3 item short form of  R-UCLA loneliness scale adapted to work | Antecedent Outcome Moderator | - Interaction frequency with AI was related to loneliness. - Loneliness predicted after-work alcohol consumption and after work insomnia. - Attachment anxiety acted as a moderator for some of the relations such that the AI manipulation had stronger effects on loneliness for those with attachment anxiety. | Journal of Applied Psychology | 9.9 |
| 174 | Taser, Aydin, Torgaloz, & Rofcanin (2022) | 2022 | 202 finance professionals in Turkey | Turkey | Cross sectional Single source | Trait-like | Yes | The 20-item  R-UCLA loneliness scale | Antecedents | - Technostress was positively related to loneliness. - The lonelier employees felt, the less flow they experienced. - Remote e-work was indirectly and serially related to   flow via technostress and loneliness. | Computers in Human Behavior | 9.9 |

|  | **Study** | **Year** | **Sample** | **Country** | **Study Design Characteristics** | **Trait or State** | **Working adults (Y/N)** | **Measure of Loneliness** | **Antecedent/ Outcome/ Moderator / Intervention** | **Primary Findings** | **Journal Name** | **Journal Impact Factor (2022)** |
| --- | --- | --- | --- | --- | --- | --- | --- | --- | --- | --- | --- | --- |
| 175 | Tian, Liu, & Yang (2023) | 2023 | 332 workers from various industries in China | China | Time lagged (1 month)  Single source | Trait-like | Yes | The Wright et al. (2006) loneliness at work scale | Antecedents Outcomes Moderators | - Perceived organizational support was negatively related to loneliness. - Loneliness was negatively related to self-rated job performance. - Extraversion moderated the negative relation between loneliness and job performance, with stronger negative   relations for those high in extraversion. | Frontiers in Psychology | 3.8 |
| 176 | Tian, Pu, & Ren (2021) | 2021 | 290 employees in China | China | Time lagged (2 waves)  Single source | Trait-like | Yes | The Wright et al. (2006) loneliness at work scale | Outcomes Moderators | - Work engagement mediated the effects of workplace loneliness on OCBs. - Gender was a moderator such that the indirect effects of loneliness on OCB via engagement were not   significant for men. | Psychology Research and Behavior Management | 4.3 |
| 177 | Torrès, Benzari, Fisch, Mukerjee, Swalhi, & Thurik (2022) | 2022 | Study 1: 2297 entrepreneurs in France, during Covid-19  Study 2: 372 entrepreneurs in France during Covid-19 | France | Studies 1 and 2: Cross sectional Single source | Trait-like | Yes | Studies 1 and 2: Single item: scale that ranged from [1] “very surrounded” to [5] “very lonely. | Outcome | - Loneliness increased the risk of burnout. | Small Business Economics | 6.4 |
| 178 | Trachik et al. (2022) | 2022 | 1160 active duty soldiers in US army | US | Cross sectional Single source | Trait-like | Yes | 3 item short form of R-UCLA loneliness scale | Antecedent Outcome | - Perceived ethical leadership indirectly affected loneliness via team cohesion. - Ethical leadership negatively affected suicidal   ideation via reduced loneliness. | Military Psychology | 1.1 |
| 179 | Tsfati & Segal-Engelchin (2022) | 2022 | 15 Israeli gay single fathers | Israel | Qualitative Single source | Trait-like | Yes | Qualitative | Antecedents | - Covid-19-related lockdowns resulted in feelings of loneliness due to lack of contact with elderly parents and social distancing from other parents. - Instrumental and emotional support from their   community networks helped. | Child & Family Social Work | 1.7 |
| 180 | Umucu, Reyes, Nay, Elbogen, & Tsai (2022) | 2022 | Over 6,000 veterans and civilians from Mturk | US | Cross sectional Single source | Trait-like | No | 4 item short form of R-UCLA loneliness scale | Antecedents | - Veterans felt lonelier. - Job loss predicted loneliness. | Stress & Health | 4.1 |
| 181 | Uslu (2021) | 2021 | 349 employees in Turkey | Turkey | Cross sectional Single source | Trait-like | Yes | The Wright et al. (2006) loneliness at work scale | Antecedents Outcomes Loneliness as moderator | - Workplace ostracism was positively related to loneliness. - Workplace loneliness was negatively related to self-rated performance. - Loneliness did not moderate the effects of workplace   ostracism. | Central European Business Review | 1.3 |
| 182 | Üstün & Üstün (2023) | 2023 | 272 music  teachers in Turkey | Turkey | Cross sectional Single source | Trait-like | Yes | The Wright et al. (2006) loneliness at work scale | Antecedents Moderators | - Employee levels of perceived overqualification were significantly related to workplace loneliness. - Perceived supervisor support moderated this relation, with a weaker association between overqualification and loneliness when supervisors were viewed as   providing strong support. | South African Journal of Education | 0.7 |
| 183 | Uysal & Kılıçkaya (2023) | 2023 | 532 private sector employees in Turkey | Turkey | Cross sectional Single source | Trait-like | Yes | The Wright et al. (2006) loneliness at work scale | Outcomes | - Workplace loneliness was significantly and negatively related to organizational identification. | Pacific Business Review | NONE |
| 184 | Varshney (2021) | 2021 | 22 mid level Indian workers | India | Qualitative Single source | Trait-like | Yes | Qualitative | Antecedents | - Covid-19 related isolation resulted in loneliness. | Psychological Studies | 1.3 |
| 185 | Voermans, den Boer, Wilthagen, & Embregts (2023) | 2022 | 8 working adults with intellectual disabilities in the Netherlands | the Netherlands | Qualitative Single source | Trait-like | Yes | Qualitative | Antecedents | - Limited social connections during Covid 19, especially as a result of lack of access to work activities contributed to loneliness. | Disability and Rehabilitation | 2.2 |
| 186 | Walz, Kensbock, de Jong, & Kunze (in press) | 2023 | 232 employees working from home during Covid in Germany | Germany | Time lagged (1 week)  Single source | Trait-like | Yes | 8 items from the R-UCLA scale adapted to work | Antecedents Moderators | - Job demands increased workplace loneliness by increasing work to home interference. - This relation was buffered by job support. - Home demands increased workplace loneliness through heightened home to work interference. - This relation was not buffered by home support. | European Management Journal | 7.5 |
| 187 | Wanberg, Csillag, Douglass, Zhou, and Pollard (2020) | 2020 | 1143 adults from Rand Corp survey | US | Time lagged (2 waves)  Single source | Trait-like | No | 3 item short form of R-UCLA loneliness scale | Antecedents Outcomes | - During Covid, loneliness was lower among   non-Hispanic Blacks, older individuals, those in good health, and those with high income.   - Loneliness was positively related to depressive symptoms and negatively related to life satisfaction - Increase in loneliness during Covid predicted   increases in depressive symptoms. | Journal of Applied Psychology | 9.9 |

|  | **Study** | **Year** | **Sample** | **Country** | **Study Design Characteristics** | **Trait or State** | **Working adults (Y/N)** | **Measure of Loneliness** | **Antecedent/ Outcome/ Moderator / Intervention** | **Primary Findings** | **Journal Name** | **Journal Impact Factor (2022)** |
| --- | --- | --- | --- | --- | --- | --- | --- | --- | --- | --- | --- | --- |
| 188 | Wang & Liu (2021) | 2021 | 430 knowledge workers in 17 companies in China | China | Time lagged (1 month)  Single source | Trait-like | Yes | The Wright et al. (2006) loneliness at work scale | Antecedents | - Job autonomy was related to workplace loneliness via perceived insider status. - The relation between perceived insider status and loneliness was moderated by perceived clan culture   (stronger relation when clan culture was high). | Chinese Management Studies | 2.2 |
| 189 | Wang, Liu, Qian, & Parker (2021) | 2021 | S1: 39 full-time employees in China  S2: 522 employees from China working from home | China | S1: Qualitative Single source  S2: Cross sectional; Single source | Trait-like | Yes | S1: Qualitative S2: 3 item short form of R-UCLA loneliness scale | Antecedents | - Participants expressed loneliness because of working from home and noted that the internet did not replicate intimacy. - In the empirical study, social support, job autonomy, and self-discipline negatively predicted loneliness. - Loneliness was positively related to emotional   exhaustion, and negatively related to life satisfaction | Applied Psychology: An International Review | 7.2 |
| 190 | Waytz, Chou, Magee, & Galinsky (2015) | 2015 | Study 1 = 309 people from MTurk  Study 2a = 56 US residents MTurk  Study 2b = 202 US residents MTurk  Study 2c = 82 US residents Mturk  Study 3 = 124 undergraduates  Study 4 = 653 people Mturk  Study 5 = 285 US residents Mturk  Study 6 = 607 US residents, Mturk | US | S1 = Cross sectional, Single source  S2a = experiment S2b = experiment S2c = experiment S3 = experiment S4 = experiment S5 = experiment S6 = experiment | Trait- and state-like | No | Study 1 = 20 item R-UCLA  Study 2a, 2b, 2c, 3, 4, 5 = 3 item  R-UCLA, adapted to “right now, do you feel…”  Study 6 = 8 items from R-UCLA | Antecedents | - Debunked the assumption that “it is lonely at the top” by showing that it is lonely at the bottom. Power and loneliness were negatively related. Low power and increase loneliness and high power can reduce loneliness. - Reduced need to belong explains power’s negative effect on loneliness. | Organizational Behavior and Human Decision Processes | 4.6 |
| 191 | Wels et al. (2022) | 2022 | 25,670 respondents in the UK | UK | Time lagged (3 waves)  Single source | Trait-like | No | 3 item short form of the revised UCLA loneliness scale | Antecedent | - Those who were furloughed during the Covid-19 pandemic expressed greater loneliness relative to those steadily employed, but lower levels of loneliness relative to those unemployed. | Social Science & Medicine | 5.4 |
| 192 | Wels et al. (2023) | 2023 | Based on data from 9 longitudinal UK studies | UK | Meta-Analysis | Trait-like | Yes | Meta-Analysis: Single item, non-work | Antecedent | - Early on, there was little evidence for a relation between working from home and feeling lonely. In later periods, working fully or partially from home increased the risk of loneliness. - The 16-29 age group and women were more prone to   loneliness when working from home. | Plos medicine | 15.8 |
| 193 | Weziak-Bialowolska & Bialowolski (2022) | 2022 | 5048 middle aged and older working adults in Europe (14 countries) | Europe | Cross sectional Single source | Trait-like | Yes | 3 item short form of R-UCLA loneliness scale | Antecedents | - Receiving recognition at work was not related to loneliness. | International Archives of Occupational and Environmental Health | 3 |
| 194 | Wickens et al. (2021) | 2021 | 3012 Canadians | Canada | Time lagged (3 waves)  Single source | State-like | No | Single item: “In the last 7 days, how often have you felt lonely?” | Antecedents | - During Covid-19, loneliness was highest among young adults (18-29), among women, and among those currently not working or laid off (compared to those who returned to work, and those whose work had not been affected). | Journal of Psychiatric Research | 4.8 |
| 195 | Williams, Hagerty, Yousha, Horrocks, Hoyle, & Liu (2004) | 2004 | 801 recruits (at risk for depression) in basic training, undergoing BOOT STRAP intervention | USA | pre-post with control Intervention Single source | State-like | Yes | 20 item R-UCLA | Intervention | - Navy recruits who were identified as at risk for depression took part in a stress management intervention. Compared to the control group who was not depressed at T1, and the at risk group who did not undergo the intervention, the intervention group showed greatest reductions in loneliness 9 weeks later. | Military Medicine | 1.2 |
| 196 | Williams, Thomas, & Liao-Troth (2017) | 2017 | 61 truck drivers | US | Qualitative Single source | Trait-like | Yes | Qualitative | Outcomes | - Loneliness emerged as a main reason for stress among truck drivers, with potential implications for turnover. | Transportation Journal | 2.3 |

|  | **Study** | **Year** | **Sample** | **Country** | **Study Design Characteristics** | **Trait or State** | **Working adults (Y/N)** | **Measure of Loneliness** | **Antecedent/ Outcome/ Moderator / Intervention** | **Primary Findings** | **Journal Name** | **Journal Impact Factor (2022)** |
| --- | --- | --- | --- | --- | --- | --- | --- | --- | --- | --- | --- | --- |
| 197 | Wilson-Forsberg & Sethi (2015 | 2015 | 16 immigrant women | Canada | Qualitative Single source | Trait-like | No | Qualitative | Moderator | - Immigrant women volunteered in order to cope with loneliness. Volunteering was a way of alleviating social loneliness and create a sense of community. | Canadian Ethnic Studies | 0.7 |
| 198 | Winefield, O'Dwyer, & Taylor (2016) | 2016 | 743 older workers in Australia | Australia | Cross sectional Single source | Trait-like | Yes | 3 item short form of R-UCLA loneliness scale | Outcomes | - Loneliness was negatively related to job satisfaction, life satisfaction, and positively related to work-life interference. | Australasian Journal on Ageing | 1.6 |
| 199 | Wood, Michaelides, Inceoglu, Niven, Kelleher, Hurren, & Daniels (2023) | 2023 | 753 and 471 university employees during twp phases of Covid-19 in UK | UK | Time lagged (1 week)  Single source | State-like | Yes | Single Item: ‘Please rate the extent to which you felt lonely in the last 7 days’ | Outcomes | - Loneliness negatively predicted homeworking satisfaction and had indirect effects on job satisfaction via homeworking satisfaction. | Applied Psychology: An International Review | 7.2 |
| 200 | Wright (2012) | 2012 | Study 1: 360 employees from  6 companies.  Studies 2 and 3: 225 and 188 employees from a government organization. | New Zealand | Studies 1-3: Cross sectional  Single source | Trait-like | Yes | Studies 1-3: The Wright et al. (2006) workplace loneliness scale | Antecedents | - Across three studies, the article showed that managers were not significantly lonelier compared to   non-managers. | Journal of Psychology | 2.7 |
| 201 | Wright & Silard (2021) | 2021 | Conceptual | NA | Cross sectional Single source | Trait-like | No | Theoretical | Antecedents Moderator | - Proposes that workplace loneliness arises from the mismatch between desire for social relations and actual social relations at work. The mismatch results in distress, and thereby loneliness. Moderators of the process are discussed. | Human Relations | 5.7 |
| 202 | Wright, Burt, & Strongman (2006) | 2006 | S1: 537 participants in New Zealand  S2: 363 participants in New Zealand | New Zealand | Studies 1-2: Cross sectional  Single source | Trait-like | Yes | Studies 1-2: The Wright et al. (2006) loneliness at work scale | Antecedents Outcomes | - Developed a self-report measure of loneliness at work with emotional deprivation and social deprivation dimensions. - There were relations to perceived work stress, lower job satisfaction, organizational commitment turnover intentions and perceived inadequate coworker and supervisor support. - Greater support from family and friends showed modest correlations with LAWS. There was also a   modest correlation to satisfaction with life. | New Zealand Journal of Psychology | 0.5 |
| 203 | Xu, Xiong, Zhang, & Chen (2023) | 2023 | 15,123 full time employees in China | China | Cross sectional Single source | Trait-like | Yes | 20 item R-UCLA loneliness scale | Outcomes | - Used latent profile analysis to create four mental health profiles, wherein one of the main indicators was loneliness. | Journal of Happiness Studies | 4.5 |
| 204 | Yang & Wen (2021) | 2021 | S1: 164 teams (leaders and members) working in different organisations in China  S2: 71 teams (leaders and members) working in a bank in China | China | S1: Time lagged (3 waves)  Multi-source (employees and team leaders)  S2: Time lagged (2 waves)  Multi-source (employees and team leaders) | Trait-like | Yes | Studies 1 -2: Workplace loneliness climate (6 items adapted from Ozcelik & Barsade, 2018); team as the referent | Antecedents Outcomes | - Leader humor predicted team loneliness climate and team performance. - Loneliness climate predicted team performance - The relationship between leader humor and loneliness climate was more negative when the team was low in centralization, and nonsignificant when the team was highly centralized. - Study 2 replicated these results, and also showed that leader humor effects on loneliness climate were most negative when team formalization was low. - Leader humor effects on team loneliness climate were more positive when leader affiliative humor was low, and more negative when affiliative humor was high. - Leader humor effects on team loneliness climate were more negative when leader aggressive humor was low, and positive when leader aggressive humor was   high. | Journal of Business Research | 11.3 |
| 205 | Yang, Lin, Chen, & Peng (2023) | 2023 | 219 employee-peer dyads in China | China | Time lagged (3 waves, one month apart)  Multi-source | Trait-like | Yes | 4 item short form of R-UCLA loneliness scale adapted to work | Outcomes | - Workplace Loneliness predicted cyberloafing. - Ego depletion mediated this relation - Leaders’ problem focused emotion management weakened WL - ego depletion relationship. | Internet research | 5.9 |
| 206 | Yang, Murad, Mirza, Chaudhary, & Saeed (2022) | 2022 | 303 employees in Pakistan during Covid lockdowns | Pakistan | Cross sectional Single source | Trait-like | Yes | 4 item short form of R-UCLA loneliness scale adapted to working from home | Antecedents Outcomes | - Cyber ostracism was significantly related to workplace loneliness - Loneliness mediated the relation between cyber ostracism and work engagement, as well as employee   mental well being | Acta Psychologica | 1.8 |

|  | **Study** | **Year** | **Sample** | **Country** | **Study Design Characteristics** | **Trait or State** | **Working adults (Y/N)** | **Measure of Loneliness** | **Antecedent/ Outcome/ Moderator / Intervention** | **Primary Findings** | **Journal Name** | **Journal Impact Factor (2022)** |
| --- | --- | --- | --- | --- | --- | --- | --- | --- | --- | --- | --- | --- |
| 207 | Zamanzadeh, Jasemi, Valizadeh, Keogh, & Taleghani (2015) | 2015 | 14 new nurses | Iran | Qualitative Single source | Trait-like | Yes | Qualitative | Antecedents | - New nurses felt lonely when embarking on a new career in an unfamiliar environment. - Lack of support from coworkers contributes to a   sense of loneliness. | Journal of Professional Nursing | 2.5 |
| 208 | Zhang (2023) | 2023 | 477 employees in China | China | Cross sectional Single source | Trait-like | Yes | The Wright et al. (2006) loneliness at work scale | Outcomes | - Workplace loneliness was significantly and positively related to emotional exhaustion, involvement in work and knowledge hiding at work. | Frontiers in Business, Economics and Management | NONE |
| 209 | Zhang & Chen (2022) | 2022 | 11,197 working respondents over age 50 participating in Survey of Health, aging and retirement in Europe (SHARE) | Europe | Cross sectional Single source | Trait-like | Yes | Single item: “in the last month, how much of the time do you feel lonely?” | Antecedents | - Men reported feeling lonelier than before during the pandemic. - Living alone and having less contact with others contributed to loneliness. - No effects of working from home or work. | Journal of Affective Disorders | 6.6 |
| 210 | Zhou, Li, Zhou, Tao, & Bouckenooghe (2023) | 2023 | 1805 employees from various organizations during Covid-19 in China | China | Cross sectional Single source | Trait-like | Yes | Lockdown loneliness (e.g., during the Covid-19 lockdown I often felt that I lacked companionship) | Antecedents Outcomes | - Perceived Organizational Support (POS) and perceived control over the virus interacted to affect lockdown loneliness such that, when control was low, employees felt lonely regardless of POS. - Lockdown loneliness predicted job insecurity. | Asian Business & Management | 3.9 |
| 211 | Zoch, Bächmann, & Vicari (2022) | 2022 | 3870 working adults in Germany surveyed during Covid-19 | Germany | Cross sectional Single source | Trait-like | Yes | Single Item: “how often have you missed the company of others during these months?” | Outcome | - During the study, there was a decline in all study participants’ life satisfaction, especially among women. This was not explained by changes in working conditions. Instead, increases in loneliness were the partial explanation. | Gender, Work, and Organization | 5.8 |
| 212 | Zumaeta (2019) | 2019 | 14 managers from Argentina and Chile | Argentina and Chile | Qualitative Single source | Trait-like | Yes | Qualitative | Antecedents | - Managers listed pressures (increased social distance, limited social support at work, exhaustion related to work), conflicts (role vs person, distance vs closeness) as reason for loneliness. - Strategies to cope included influencing others and fulfilling personal passion, support from one’s network,   healthy lifestyle, mental and physical disconnection. | Journal of Leadership & Organizational Studies | 4.8 |
| 213 | Zürcher et al. (2021) | 2021 | 238 vocational, academic, and career counsellors in Switzerland | Switzerland | Cross sectional Single source | Trait-like | Yes | Single item: “Sometimes I feel lonely when working from home” | Antecedents | - Only ¼ of participants agreed that sometimes they felt lonely when working from home. - Sharing the office with many coworkers contributed to feelings of loneliness when working from home. | Frontiers in Psychology | 3.8 |

**Table S3. Definitions of Loneliness**

Authors Description

Baumeister & Leary (1995)

Bonsaksen et al. (2023)

Loneliness reflects “an individuals’ subjective perception of deficiencies in his or her social relationships” (Russel et al., 1984)**.**p**.** 507 Consequently, at its core, loneliness emerges when a person feels a lack of relationships with others, and it highlights the emotional distress present due to this lack of connection with others. p.1

Buecker et al. (2020) People feel lonely when they perceive a discrepancy between their desired and actual quality or quantity of their social relations. p.669

Cacioppo et al. (2006) A complex set of feelings that occurs when intimate and social needs are not adequately met. p.1055

Cardon & Arwine (2022)

As a subjective construct, loneliness is a self-perceived relational deficiency (Cacioppo et al., 2006; Perlman & Peplau, 1982), that occurs when individuals feel they are deprived of desired interpersonal relationships and social interactions. p.5

Chen et al. (2016) Unpleasant feeling resulting from a person’s perception of lacking satisfactory social relationships in the workplace. p. 864

Cheng et al. (2023) Workplace loneliness refers to employees' subjective affective evaluations and feelings of whether their affiliation needs are met by their peers and their organizations. p.2

De Jong-Gierveld (1978)

De Jong-Gierveld & Kamphuis (1985)

Di Tommaso & Spinner (1993)

Dor-Halm & Olatka (2021)

The experience of a lag between realized and desired interpersonal relationships as disagreeable or unacceptable, particularly when the person perceives a personal inability to realize the desired interpersonal relationships within a reasonable period of time. p.221

Loneliness involves situations in which the number of achieved relationships is smaller than desired, or when the existing relationships fail to attain the desired degree of intimacy. p.289

Don’t define it explicitly. Adopt multidimensional approach with romance, family and social subscales.

A discrepancy between an individual’s preferred relations and actual social relations, which may lead to a negative experience of feeling alone as well as to the distress and dysphoria of feeling socially isolated. p. 261

Gabriel et al. (2021) Leader loneliness - feelings of lacking companionship from one’s followers at work. p. 1517

Hawkley & Cacioppo (2010)

Heinrich & Gullone (2006)

A distressing feeling that accompanies the perception that one’s social

needs are not being met by the quantity of especially the quality of one’s social relationships. p. 218

Loneliness is multidimensional (social and emotional components).

Hughes et al. (2004) When one’s intimate and social needs are not adequately met, a complex set of feelings termed loneliness occurs that motivates one to seek the fulfilment of these needs (Baumeister and Leary; 1995). p. 656

| Authors | Description |
| --- | --- |
| Lam & Lau (2012) | It is an aversive psychological state due to a person’s perception of lacking satisfactory social relationships. p. 4266 |
| Morrish & Medina- Laura (2021) | Loneliness is commonly understood as the subjective or perceived experience of isolation or lack of social support. p. 2 |
| Ong (2022) | Loneliness is a subjective, negative affect that arises from an attribution  of deficient social relationships. p. 1181 |
| Ozcelik & Barsade (2018) | Employees’ subjective affective evaluations of, and feelings about, whether their affiliation needs are being met by the people they work with and the organization they work for. p.2345 |
| Peng et al. (2017) | A workplace-specific emotion that coexists with certain characteristics of the working environment such as competitive climate, virtual teams, and  alternative work arrangements. p. 510 |
| Peplau & Perlman (1982) | Loneliness has three characteristics:   1. deficiencies in social relationships 2. subjective experience 3. unpleasant and distressing p. 3 |
| Russell et al. (1978, 1980) | Does not provide definitions |
| Weiss (1973) | Loneliness appears always to be a response to the absence of some particular type of relationship or, more accurately, a response to the absence of some particular relational provision. p. 17 |
| Wright et al. (2006) | Loneliness is a psychological state that results from deficiencies in a  person’s social relationships, either qualitatively or quantitatively. p. 59 |
| Wright & Silard (2021) | Workplace loneliness is the psychological pain of perceived relational deficiencies in the workplace. p. 1064 |

**Table S4. Summary of Scales Used in Loneliness Research.**

**Citation Dimensionality Psychometric Properties Examples of use in Work Contexts**

***The UCLA***

***Loneliness Scale* (Russell, Peplau, & Ferguson, 1978)**

***The Revised R- UCLA Loneliness Scale* (Russell, Peplau, & Cutrona, 1980)**

*Unidimensional* (20 items)

“I am unhappy doing so many things alone.”

*Unidimensional* (20 items) “I lack companionship.”

- Unidimensional
- Alpha: .96
- Concurrent validity: High scorers on the loneliness scale described themselves as more lonely than other people. It was also significantly correlated with self- reported feelings of depression .49 and feelings of anxiety .35
- Discriminant validity: None presented in original article.
- Observations: The Revised UCLA Scale was designed to counter the possible effects of response bias in the original scale. While the Revised UCLA Loneliness Scale is the most commonly used scale, the original scale has not been used frequently in work-related research.
- Unidimensional
- Alphas: Study 1 = .94; Study 2 = .94
- Concurrent validity: Significant correlations between scores on scale and time spent alone and who were not in romantic relationships.
- Discriminant validity: Correlated, but distinct from measures such as negative affect, social risk taking, and affiliative tendencies.
- Observations: This is the most commonly used scale. However, lack of consistency of the specific items being used from the R-UCLA is a concern – there are studies using three, five, eight, nine, or 11 items from the original scale, potentially changing its psychometric properties and making the direct comparison of findings more challenging.
- N/A as this scale was revised by

Russell et al. (1980) before it was used regularly

- Ozcelik & Barsade (2018) – 672 employees, 114 supervisors in the US
- Cacioppo et al. (2015) – 662 soldiers in US military

***de Jong-Gierveld Loneliness Scale* (original; de Jong-Gierveld & Kamphuis, 1985)**

***Social and Emotional Loneliness Scale for Adults (SELSA)***

# (DiTommaso & Spinner, 1993)

***Short UCLA Loneliness Scale* (Hughes, Waite, Hawley, & Cacioppo, 2004).**

*Overall* (11 items):

*Emotional subscale* (6 items):

“I miss having a really close friend.”

*Social subscale* (5 items): “There is always someone I can talk to about my day-to- day problems.” (reverse scored)

*Overall* (37 items)

*Social subscale* (14 items) “I’m not part of a group of friends and I wish I were.” *Emotional- Romantic subscale* (12 items)

“I have an unmet need for a close romantic relationship.” *Emotional- Family subscale* (11 items)

“I feel alone when I’m with my family.”

*Overall* (3 items):

“First, how often do you feel that you lack companionship:

Hardly ever, some of the time, or often?”

- Multidimensional
- Alphas: not reported in original article
- Concurrent validity: not reported
- Discriminant validity: not reported
- Observations: Alsubheen et al. (2023) noted moderate to high evidence for bifactor structure of the scale with internal validity but concerns regarding low content validity evidence.
- Multidimensional, correlations among subscales range from

.21 to .50

- Alphas: Romantic = .93; Family = .89; Social = .91
- Concurrent validity: The romantic scale of the SELSA was correlated at .69 with the UCLA scale but only weakly associated with social loneliness at .14
- Discriminant validity: Correlations for the social subscale of the SELSA showed a reverse pattern compared to the UCLA scale to those of the romantic scale.
- Observations: This scale has been used infrequently. We found only two articles in our sample which had used this scale. Thus, it seems most useful for those specifically interested in romantic or family loneliness given the specific subscales.
- Unidimensional
- Alpha: Study 1 = .72; Study 2 = .72
- Concurrent validity: Correlated with the R-UCLA at .82 and significantly related with depressive symptoms and stress scales
- Discriminant validity: Scores were only weakly associated with emotions not linked to loneliness such as enjoyment, energy, and motivation.
- Observations: This scale has been used infrequently. We found only two articles in our sample which had used this scale. Thus, it seems most useful for those specifically interested in romantic or family loneliness given the specific subscales.
  - Stefanowski et al. (2023) – 460 firefighters in Poland
  - Bazemore et al. (2010) – 162 college professors in USA
  - Creed & Reynolds (2021) – 148 youths in Australia
  - Soler-Gonzalez et al. (2017) – 433 physicians and nurses in Spain
  - Deutrom et al. (2022) – 299 workers in the UK
  - Kuriakose et al. (2019) – 554 IT employees in India

***de Jong-Gierveld Loneliness Scale (short)***

# (de Jong- Gierveld & van Tilburg, 2006).

***Loneliness at Work Scale* (Wright, Burt, & Strongman, 2006).**

*Overall* (6 items):

*Emotional subscale* (3 items):

“I experience a general sense of emptiness.”

*Social subscale* (3 items): “There are plenty of people I can rely on when I have problems.” (reverse scored) *Overall Loneliness at Work* (16 items)

*Emotional Deprivation* (9 items)

“I often feel abandoned by my coworkers when I am under pressure at work.” *Social Companionship* (7 items)

“I have social companionship/fellowship at

- - Multidimensional
  - Alphas: .70-.76
  - Concurrent validity: Correlated with the longer version of the scale at .93 to .95.
  - Discriminant validity: None reported.
  - Observations: This scale has similar psychometrics to the longer scale but is much shorter making it attractive to those interested in using the scale but having limited room to do so.
    - Multidimensional
    - Alphas: Emotional deprivation = .93; Social companionship = .87
    - Concurrent validity: Correlated with higher work stress and lower levels of job satisfaction, organizational commitment, and lower intentions to turnover.
    - Discriminant validity: None reported.
    - Observations: This scale was designed to specifically measure loneliness at work which is unique to the UCLA and de Jong-Gierveld scales.
  - Bonsaksen et al. (2023) – 1,649 Adults in Norway, USA, UK, and Australia
  - Bower et al. (2023) – 1,523 adults in Australia
  - Arslan et al. (2020) – 864 nurses in Turkey
  - Firoz & Chaudhary (2022) – 934 employees in India

work.” (reverse scored)

***Note****.* Psychometric information was taken from the original scale development articles themselves.

30

Records (213) included in the review (236 studies represented) PsycInfo (*n* = 108)

EbscoHost (*n* = 79)

Added from GoogleScholar, and cited references of other articles (*n* = 26)

Records excluded:

PsycInfo: Did not focus on work and loneliness (*n* = 57) EbscoHost: Duplicates (*n* = 47)

EbscoHost: Did not focus on work and loneliness (*n* = 45)

Records assessed for eligibility PsycInfo: (*n* = 165)

EbscoHost (*n* = 171)

Records not retrieved PsycInfo: (*n* = 14) EbscoHost (*n* = 4)

Records excluded

(*n* = 1,511 PsycInfo, *n* = 2,405 EbscoHost)

Records screened

(*n* = 1,690 PsycInfo, *n* = 2,580 EbscoHost)

**Screening**

**Included**

**Figure F1**

**Prisma Figure of Literature Identification**

Records removed *before screening*:

Not journal articles OR not adults *n* = 1,377 PsycInfo

*n* = 807 EbscoHost

**Identification**

**Identification of articles via databases and registers**

Records published until December 31, 2023, identified from:

PsycInfo Database (*n* = 3,067)

EbscoHost (*n* = 3,387)

Records sought for retrieval PsycInfo: *n* = 179 EbscoHost *n* = 175
